# Comparative single-cell transcriptional and proteomic atlas of clinical-grade injectable mesenchymal source tissues

**DOI:** 10.1126/sciadv.adn2831

**Published:** 2024-07-12

**Authors:** Severin Ruoss, Chanond A. Nasamran, Scott T. Ball, Jeffrey L. Chen, Kenneth N. Halter, Kelly A. Bruno, Thomas C. Whisenant, Jesal N. Parekh, Shanelle N. Dorn, Mary C. Esparza, Shannon N. Bremner, Kathleen M. Fisch, Adam J. Engler, Samuel R. Ward

**Affiliations:** ^1^ Department of Orthopaedic Surgery, UC San Diego, La Jolla, CA, USA.; ^2^ Center for Computational Biology and Bioinformatics, UC San Diego, La Jolla, CA, USA.; ^3^ Department of Anesthesiology, Center for Pain, UC San Diego, La Jolla, CA, USA.; ^4^ Department of Obstetrics, Gynecology & Reproductive Sciences, UC San Diego, La Jolla, CA, USA.; ^5^ Chien-Lay Department of Bioengineering, UC San Diego, La Jolla, CA, USA.; ^6^ Sanford Consortium for Regenerative Medicine, La Jolla, CA, USA.; ^7^ Department of Radiology, UC San Diego, La Jolla, CA, USA.

## Abstract

Bone marrow aspirate concentrate (BMAC) and adipose-derived stromal vascular fraction (ADSVF) are the most marketed stem cell therapies to treat a variety of conditions in the general population and elite athletes. Both tissues have been used interchangeably clinically even though their detailed composition, heterogeneity, and mechanisms of action have neither been rigorously inventoried nor compared. This lack of information has prevented investigations into ideal dosages and has facilitated anecdata and misinformation. Here, we analyzed single-cell transcriptomes, proteomes, and flow cytometry profiles from paired clinical-grade BMAC and ADSVF. This comparative transcriptional atlas challenges the prevalent notion that there is one therapeutic cell type present in both tissues. We also provide data of surface markers that may enable isolation and investigation of cell (sub)populations. Furthermore, the proteome atlas highlights intertissue and interpatient heterogeneity of injected proteins with potentially regenerative or immunomodulatory capacities. An interactive webtool is available online.

## Teaser

A comparative analysis of the two most used musculoskeletal “stem cell” therapies highlights their biological differences.

## INTRODUCTION

Autologous bone marrow aspirate concentrate (BMAC)–and adipose-derived stromal vascular fraction (ADSVF)–based tissue transplantations are the most marketed cell-based interventions to treat a wide range of orthopedic conditions: pain, inflammation, and neurological, immunological, and respiratory diseases as well as other conditions worldwide (*1*–*3*). The mesenchymal stromal cell (MSC) has been proposed as the key active ingredient of both BMAC and ADSVF cell preparations and has been, maybe misleadingly, marketed as a “stem cell” (*4*–*9*). This stem cell market was valued 11.9 billion USD globally in 2021 and was predicted to grow by 11.4% annually between 2022 and 2030 (*10*). Paralleling the growing market, a large body of clinical trials has emerged to investigate the clinical benefits of autologous, minimally manipulated BMAC (*11*–*21*) and ADSVF transplantations (*13*, *15*, *22*–*30*). Outcomes have been very controversial as some studies suggest promising outcomes while others show no effect. Evidence has been strong enough on both sides so that current consensus statements have not picked a side but say that more studies are needed, and a large body of publications criticize the lack of in-depth characterization of the injected tissue (*4*, *9*, *31*–*42*). This characterization has, at least theoretically, been made possible when reporting standards for BMAC and adipose preparations were agreed upon in the clinical-translational field (*31*, *37*, *43*), but given the unknown mechanism of action, it has remained challenging to identify a parameter or active ingredient that is predictive of the quality/therapeutic potential of the injectable (*34*, *42*). Lacking more informed alternatives, many trials and recommendations have evolved around the concept of MSC plastic adherence and in vitro differentiation capacity (*44*). These trials then have incorporated measurements of cell yield, such as mononuclear cell counts, CD34^+^ hematopoietic cells, colony-forming units (CFUs), and in vitro differentiation potential, to define the quality of the injectable (*12*, *19*, *29*, *31*, *37*, *45*). Many clinical experts believe that CFUs and in vitro differentiation are important characteristics of clinical-grade MSCs (*31*, *36*, *37*, *46*–*49*), and both BMAC and adipose (along with skeletal muscle, cord blood, placenta, periosteum, pancreas, and other tissues) contain cells with those in vitro features (*5*, *7*, *50*–*54*). As a result, they have become some of the most used “stem cell therapies” in patients and athletes (*1*, *2*, *32*, *36*, *40*, *55*) and have even been advertised and used interchangeably (*2*, *3*, *15*, *56*–*58*) even though there is no rigorous and comprehensive head-to-head comparison of what is in either of them. Knowing this information would allow scientists and clinicians to have some reference standards to start characterizing samples on the basis of ingredients that they think are important.

In summary, detailed analysis and clarification of cell and protein doses in these widely used injectables are urgent and critical to advance and to justify precision regenerative medicine and to combat current anecdata and misinformation (*4*). Therefore, we inventoried and compared composition and heterogeneity of the two most popular “stem cell therapeutics,” BMAC and ADSVF, obtained from the same individuals and constructed a single-cell transcriptional and proteomic atlas. This will serve as a resource that helps tailor long-needed clinical studies and helps clarify the source of efficacy, if any (*4*). The in-depth surface marker analysis may direct future isolation and investigation of specific cell (sub)populations and thereby offer novel cell engineering opportunities even outside the current regenerative medicine environment. The datasets analyzed in this work can interactively be explored on http://muscle.ucsd.edu/BMACandADSVF.

## RESULTS

### Current guidelines to quantify MSCs do not work in clinical-grade source tissues

**Fig. 1.**
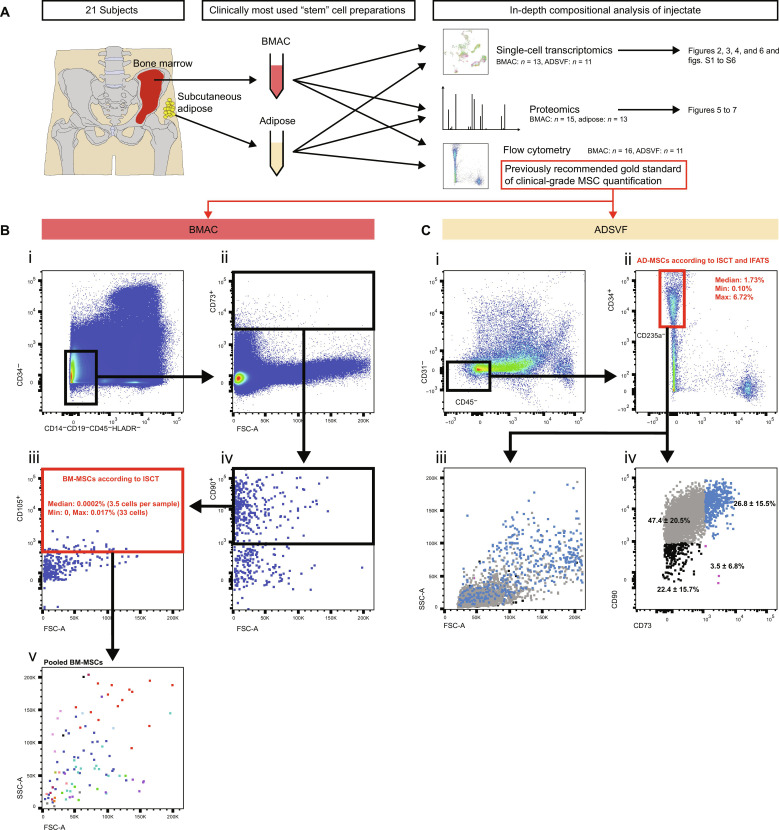
Study overview and previous flow cytometry standard to quantify MSCs in therapeutic BMAC and adipose preparations. (**A**) Study design and overview. (**B**) Flow cytometry quantification of MSCs in BMAC, using the current clinical gold-standard surface marker strategy. The red box indicates ostensible MSCs based on ISCT/IFATS marker criteria. Cells fulfilling these criteria were too rare to be reliably detected in by flow cytometry. Colors in (v) indicate different participants. *n* = 16. (**C**) Flow cytometry quantification of MSCs in ADSVF, using the current clinical gold-standard surface marker strategy. The red box indicates ostensible MSCs based on ISCT/IFATS marker criteria. Cells were too heterogeneous in size and granularity to be confidently identified and quantified as one defined population. Furthermore, the addition of more positive IFATS markers did not improve their forward- and side-scatter clustering profile. Colors in (iii) represent distinct expression of CD73 and CD90 as defined in (iv). *n* = 11. FSC-A, forward scatter–area; SSC-A, side scatter–area; BM-MSCs, bone marrow–derived mesenchymal stromal cells; AD-MSCs, adipose-derived mesenchymal stromal cells.

Bone marrow and adipose were harvested from the same 21 participants. Bone marrow was spun to clinical-grade and ready-to-inject BMAC, and ADSVF was enzymatically released from adipose. Both tissues were fractionated for flow cytometry, single-cell RNA sequencing (scRNA-seq), and proteomics analysis (Fig. 1A). First, we attempted to quantify MSC dose in these therapeutic tissues, using the previously recommended standards for clinical practice (*43*). These are flow cytometry–based CD73^+^CD90^+^CD105^+^CD14^−^CD19^−^CD34^−^CD45^−^HLA-DR^−^ for BMAC-MSCs (*44*) and CD34^+^CD31^−^CD45^−^CD235a^−^ (optional marker additions include CD13^+^CD73^+^CD90^+^CD105^+^) for ADSVF-MSCs (*59*) as defined by the International Society for Cellular Therapy (ISCT) and the International Federation for Adipose Therapeutics (IFATS). In BMAC (Fig. 1B), only 3.5 cells per patient sample (0.0002%) were defined as MSCs using these flow cytometry marker definitions. This frequency was too low to be a reliably detectable population by flow cytometry (*60*). Pooling and displaying these “MSCs” on forward and side scatter suggested that this population contains cells of various sizes and granularity; thus, we concluded that these cells are unlikely to all be MSCs but false positives (Fig. 1Bv). In ADSVF (Fig. 1C), a robust population of 1.73% was detected. Similarly, this population contained cells of various sizes and granularity, and the recommendation that CD73, CD90, and CD105 add value to the MSC profile/purity (*59*) could not be validated as these additional markers did not characterize a more distinct population by cell size or granularity (Fig. 1C, iii and iv). We concluded that these ISCT standards to quantify MSCs fail, although for two different reasons, in therapeutically relevant source tissues.

### BMAC and ADSVF stem cells are not the same

**Fig. 2.**
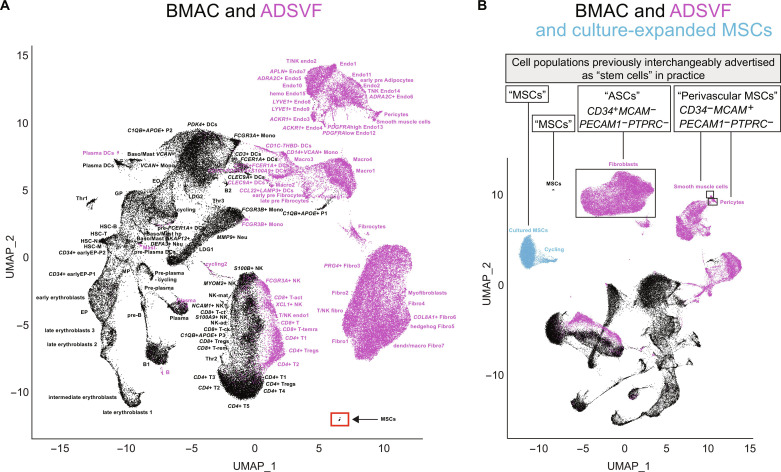
Cellular comparison of BMAC and ADSVF using scRNA-seq. (**A**) UMAP plot showing minimal overlap between the 62 BMAC and 57 ADSVF cell (sub)populations. An MSC cluster was found in BMAC, but not ADSVF (red box). BMAC-MSCs accounted for 0.22 ± 0.22% of RBC-digested BMAC cells and each sample contributed between 1 and 44 cells to this cluster. (**B**) Recalculated UMAP plot after addition of cultured MSCs (blue) and indicating different populations that are interchangeably used under the “stem cell” umbrella in previous clinical practice. Fibroblasts, pericytes, and smooth muscle cells accounted for 53.46 ± 12.74%, 1.11 ± 0.75%, and 0.22 ± 0.27%, respectively, of ADSVF cells. *n* = 13 BMAC, 11 ADSVF, and 1 cultured MSCs samples. B, B cell; Baso/Mast, basophil/mast cells; ck, cytokine; ct, cytotoxic; DCs, dendritic cells; Endo, endothelial cells; EO, eosinophils; EP, erythroblast progenitor; Fibro, fibroblasts; GP, granulocyte progenitors; HSC-N/B/M/T, hematopoietic stem cells-native/B−/monocyte/T cell lineage; LDG, low-density granulocytes; Macro, macrophages; Mono, monocytes; MP, megakaryocyte progenitor; Neu, neutrophils; NKad, adaptive natural killer cells; NKmat, mature natural killer cells; P1–3, population 1–3; Plasma, plasma cells; T, T cell; Temra, terminally differentiated effector T cells; Thr, thrombocytes; Trem, resting effector memory T cells.

Next, we compared BMAC and ADSVF transcriptomes. Red blood cell (RBC)–depleted fractions of BMAC and ADSVF were subjected to scRNA-seq using the 10x Genomics V3 platform. Each sample was sequenced individually, and datasets were then pooled for visualization, unbiased clustering, and annotation. A total of 67,841 BMAC and 56,168 ADSVF cells were sequenced at a mean read depth of 25,936 and 30,002 reads per cell, respectively (table S1). A total of 50,836 BMAC and 40,129 ADSVF cells passed quality filtering (*61*) and were visualized using uniform manifold approximation and projection (UMAP) (Fig. 2A and tables S2 and S3). A total of 62 BMAC and 57 ADSVF cell populations and subpopulations were identified (Fig. 2A, tables S2 and S3, and figs. S1 and S2) by manual annotation using previously published marker genes and single--cell transcriptome datasets (see Materials and Methods and notes S1 and S2). While BMAC and ADSVF are used as cell therapies interchangeably, their overlap in cell populations was in fact minimal. Even similar cell subpopulations found in both tissues, for example, regulatory T cells (T_regs_), were transcriptionally distinct enough to only cluster near each other based on the first two UMAP dimensions (Fig. 2A) and were transcriptionally more related to other *CD4*^+^ T cell populations from the same tissue rather than to their counterparts from the other tissue (fig. S3).

Unlike flow cytometry, the scRNA-seq omics approach allowed the detection of MSCs based on unbiased detection of marker genes, which included *PDGFRA*, *THY1*, and *NGFR*. MSCs accounted for 0.22 ± 0.22% (mean ± SD; coefficient of variation 99%) of RBC-depleted BMAC, and they clustered uniquely (Fig. 2A, table S2, and note S1). This MSC population was likely different from previously described skeletal stem cells (SSCs; CD164^+^PDPN^+^CD73^+^CD146^−^), and bone, cartilage, and stromal progenitors (PDPN^+^CD146^+^) (*62*) as *PDPN* was detected in only 0.7% of MSCs and they did not conform with the remaining marker profiles. Furthermore, *CD164*, a receptor purported to mark SSCs, was expressed in all 62 cell populations and thus was not specific to BMAC-MSCs (table S4).

In ADSVF, the *MCAM* (CD146)–expressing pericytes (1.11 ± 0.75% of ADSVF cells, coefficient of variation 67%) have been regarded as the MSCs (*53*, *63*). Our transcriptional data revealed a contradiction to the guidelines by ISCT/IFATS, who along with others, have defined the adipose-derived MSC [often termed adipose-derived stem cells (ASCs)] as CD34^+^ CD31^−^CD45^−^CD235a^−^ (*59*, *64*–*69*). This definition marked fibroblastic populations (53.46 ± 12.74% of ADSVF cells, coefficient of variation 24%) but excluded most pericytes as *CD34* was detected in only 23.6% of them (Fig. 2B, table S5, and note S2). Conversely, these *CD34*^+^ fibroblastic populations lacked *MCAM* expression (table S5), suggesting that two different cell types have been regarded as MSCs, depending on the marker strategy used. Furthermore, the *MCAM*-based MSC definition did not distinguish between pericytes and smooth muscle cells (Fig. 2B and table S5). A substantial body of literature has focused on cultured MSCs (*5*, *7*, *22*, *44*, *50*, *51*, *54*, *59*, *70*–*72*), so we then compared all BMAC- and ADSVF-derived populations marketed as stem cells with commercially available, marrow-derived cultured passage 3 MSCs that express the ISCT markers (Fig. 2B). BMAC-MSCs were the population most closely related to cultured MSC, but this relation appeared inconsiderable given that cell types with known differences, such as mast cells, T cells, and natural killer (NK) cells were transcriptionally more closely related than fresh with cultured MSCs (fig. S3). Similarly, the transcriptional relation between ADSVF-fibroblastic populations, pericytes, BMAC-MSCs, or culture-expanded MSCs was negligible (Fig. 2B and fig. S3). On the basis of these data, the practice of advertising and clinically using minimally manipulated MSCs, pericytes, fibroblasts/ASCs, and cultured MSCs interchangeably as stem cells appears to be counterintuitive because: (i) they were negligibly related transcriptionally and (ii) BMAC-MSCs and ADSVF-pericytes accounted for 0.22 and 1.11% of cells even after RBC digestion, a dose that appears negligible given the other >98% cells being injected as BMAC and ADSVF. To address the first point, minimally manipulated noncultured BMAC-MSCs, ADSVF-pericytes, and ADSVF-fibroblasts/ASCs must be pulled out of clinical-grade biologics to prove whether (i) their in vivo function upon transplantation is equivalent and (ii) whether these populations are identical to the colony-founding cells described previously (*47*–*49*). On the basis of Fig. 1 (B and C) and previous data (*34*), this may require a new fluorescence-activated cell sorting (FACS) strategy. The second point requires thorough identification of all cell populations/subpopulations injected as biologics into patients. The translational scientific community would benefit from detailed transcriptional information and FACS strategies to isolate specific subpopulations. This information would enable cell subpopulation-specific investigations, engineering approaches, and individual transplantations to test in vivo efficacies. Therefore, we next created a transcriptional atlas of these clinical-grade cell preparations including a detailed differentially expressed gene (DEG) analysis and, importantly, a separate DEG analysis for surface markers only to facilitate specific cell isolations in future studies investigating human bone marrow and adipose cells.

### The transcriptional atlas shows minimal overlap between clinical-grade BMAC and ADSVF cells

**Fig. 3.**
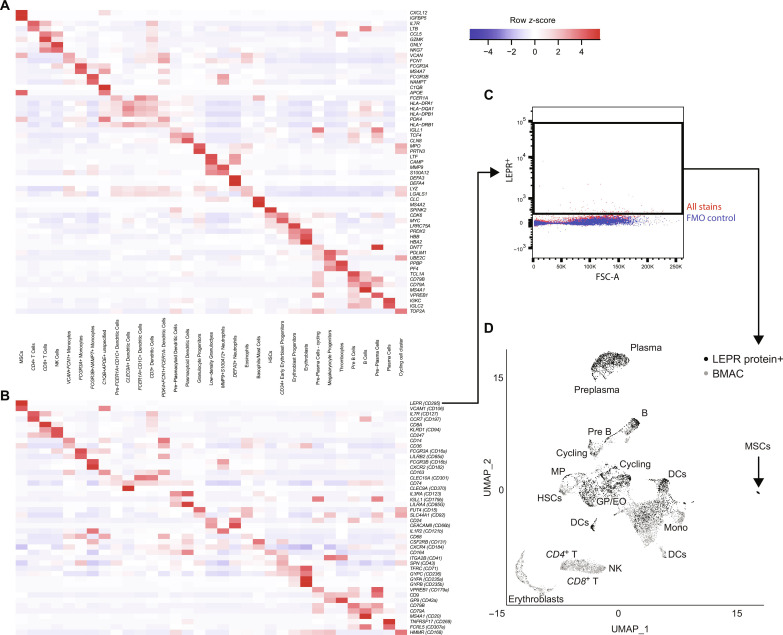
DEGs in BMAC cells identified after unbiased clustering. (**A**) Top two DEGs across BMAC cell families. (**B**) Top two differentially expressed surface marker genes across BMAC cell families. (**C**) FACS-gating for LEPR protein expression. (**D**) UMAP plot comparing cell populations before (gray) and after (black) sorting of LEPR-expressing cells. *n* = 13 [(A) and (B)], *n* = 2 [(C) and (D)]. B, B cell; GP, granulocyte progenitors; HSCs, hematopoietic stem cells; Mono, monocytes; MP, megakaryocyte progenitor; Plasma, plasma cells; T, T cell.

**Fig. 4.**
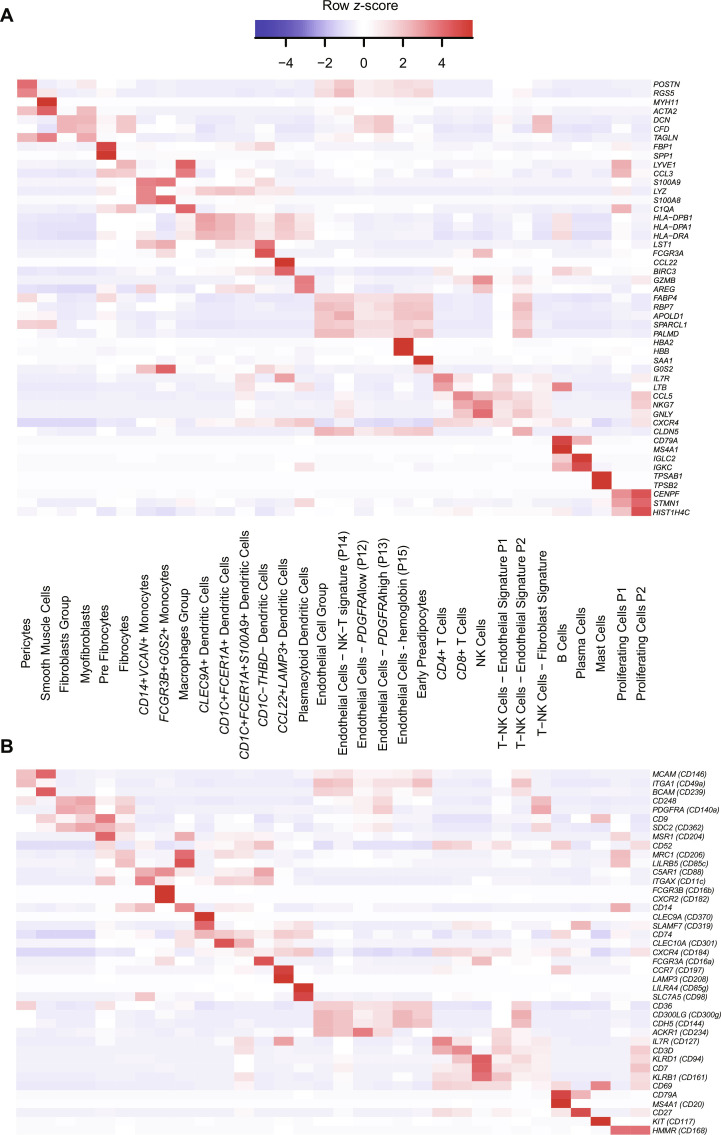
DEGs in ADSVF cells identified after unbiased clustering. (**A**) Top two DEGs across ADSVF cell families. (**B**) Top two differentially expressed surface marker genes across ADSVF cell families. *n* = 11.

In BMAC, DEGs between subpopulations (e.g., *CD4*^+^ regulatory versus other *CD4*^+^ T cell subpopulations) were too subtle to be captured with other cells present; therefore, we pooled these subpopulations into a general population first (e.g., *CD4*^+^ T cells) and later explored them separately to suggest subpopulation-specific gene and surface markers. Using this approach, we characterized 33 populations that were directly identifiable in BMAC using positive DEGs (Fig. 3A). Two clusters of proliferative cells were driven by high expression of cell cycle markers *TOP2A*, *MKI67*, *CENPF*, and *ASPM*. After separate subclustering of the pooled populations, we annotated a total of 62 cell (sub)populations in BMAC (fig. S1 and table S2). Conversely in ADSVF, UMAP clustering revealed four broad cluster groups: fibroblast-like, endothelial-like, T/NK, and monocyte/macrophage/dendritic cells (fig. S2). These groups were further subclustered into 57 more detailed cell (sub)populations (table S3), of which 32 can directly be identified in ADSVF using DEGs (Fig. 4A). We found two clusters driven by proliferating/cycling cell genes including *CENPF*, *TOP2A*, *MKI67*, *ASPM*, *NUSAP1*, and *TYMS*, which accounted for 0.41 ± 0.34% and 0.12 ± 0.10% of ADSVF cells, respectively (Fig. 4A and table S3).

With the goal to find better surface markers to isolate MSCs, we first tested gene expression of the traditional MSC markers *PDGFRA* (CD140A), *ENG* (CD105), *THY1* (CD90), *NGFR* (CD271), *NT5E* (CD73), and *MCAM* (CD146) (*53*, *73*, *74*), which were detected in 48.1, 42.2, 31.1, 25.9, 14.1, and only 4.4% of BMAC-MSCs (table S4). Of those, only *PDGFRA* was among the top 100 most DEGs (ranked 79; table S6). Another commonly reported MSC marker, *CD44*, was detected in all 62 BMAC populations, making it appear nonspecific. The top transcriptional MSC markers included *CXCL12*, *IGFBP5*, *APOE*, *FABP4*, and *LEPR* (Fig. 3A, fig. S4, table S6, and note S1). Conversely, the established negative MSC markers brought forward by the ISCT and used to exclude antigen-presenting cells, hematopoietic cells, monocytes/macrophages, thrombocytes, and endothelial cells, i.e., *HLA-DRA*, *PTPRC* (CD45), *CD14*, and *PECAM1* (CD31), were actually detected in between 16 and 50% of BMAC-MSCs (table S4). This poor marker stratification may explain why current flow cytometry quantification for MSCs fail in these tissues (Fig. 1B). To combat this issue, we added a secondary analysis of DEGs of surface proteins to develop efficient strategies to isolate fresh MSCs and other cell populations found in this atlas (Figs. 3B and 4B and table S7). A gene list encompassing cluster of differentiation 1A (*CD1A*) through CD371 (*CLEC12A*) was used for this analysis with the best surface markers to isolate fresh MSCs from BMAC being *LEPR* (CD295), *VCAM1* (CD106), *DDR2* (CD167b), *TNC* (CD175), and *LIFR* (CD118) (Fig. 3B, fig. S4, and table S7). As a proof of concept, we isolated live single cells expressing leptin receptor (LEPR) protein from BMAC of two individuals using FACS, performed scRNA-seq, and compared it to the unsorted data of the same individuals (Fig. 3, C and D); the LEPR^+^ sorting criterion increased MSC concentration 21-fold (Fig. 3D and table S8). While LEPR as a single positive marker depleted T cells and NK cells (table S8), our data suggest that the next step to improve this sorting strategy may be to add a negative marker which actively depletes B and plasma cells (such as CD19) because they were enriched by LEPR^+^ sorting along with the MSCs (table S8). In future, such purified cells of interest will also have to undergo functional testing to establish clinical relevance. Conversely, LEPR was less specific in ADSVF and predominantly expressed in endothelial cell and fibroblast clusters (fig. S4). Another small BMAC-specific population expressed MSC marker genes *CXCL12*, *APOE*, *VCAM1*, and *SELENOP*. This population accounted for 0.57 ± 0.64% of cells and was further characterized by unique *C1QB* expression (Fig. 3, A and B, fig. S1, table S2, and note S1). We note that this population has not been described in previous detailed transcriptional analysis of human bone marrow cells (*34*, *75*, *76*).

The largest comparable populations in BMAC and ADSVF were *CD4*^+^ T cells (18.69 ± 10.60% versus 4.73 ± 3.67%), *CD8*^+^ T cells (5.37 ± 3.44% versus 2.80 ± 2.74%), and NK cells (8.83 ± 6.49% and versus 2.59 ± 1.66%) but looking closer, there were still differences within these cell types between the two injectables. BMAC presented with more heterogeneity within these cells: six versus three *CD4*^+^ T subpopulations, four versus three *CD8*^+^ T subpopulations, and six versus two NK subpopulations in ADSVF. *FOXP3*^+^*CTLA*^+^ regulatory T cells were the only *CD4*^+^ population in both tissues that could be named by their transcriptional profile (figs. S5 and S6, tables S2 and S3, and notes S1 and S2). In BMAC, we found “cytotoxic”, “cytokine” and “resting effector memory *CD8*+ T cells” in accordance with Szabo and colleagues (*77*), but we also found an human leukocyte antigen (HLA)–expressing *CD8*^+^ regulatory T cell subpopulation (fig. S5, table S2, and note S1). In ADSVF, activated and effector memory *CD8*^+^ T cells were found along with a third subpopulation that remained unnamed on the basis of gene expression (fig. S6, table S3, and note S2). Compared with ADSVF, there was an impressive heterogeneity of NK cells in BMAC, including a *S100B*^+^ subpopulation (figs. S5 and S6, tables S2 and S3, and notes S1 and S2) that has not been described in previous detailed analysis of human marrow-derived NK cells (*75*, *78*).

While monocytes are a major cell population in BMAC (19.06 ± 7.93%), they accounted for only 0.68 ± 1.02% in ADSVF and were possibly blood derived as some vasculature is typically harvested in adipose. *VCAN*^+^ and *FCGR3B*^+^ subpopulations were found in both tissues, and an *FCGR3A*^+^ subpopulation was detected in BMAC only (Figs. 3 and 4, tables S2 and S3, and notes S1 and S2). The dendritic cell population was highly heterogeneous, composed of seven and six subpopulations and cumulatively accounting for 6.53 ± 2.89% of BMAC and 3.43 ± 1.28% of ADSVF cells, respectively. Most subpopulations corresponded to the detailed definitions in (*79*), but in addition, a *CD3*^+^ population, a pre-*FCER1A*^+^*CD1C*^+^, and a preplasmacytoid population were detected in BMAC (Fig. 3, fig. S1, and note S1). In ADSVF, in addition to Villani and colleagues’ DC1, DC2, DC3, DC4, and DC6 populations (*79*), a *CCL22*^+^*LAMP3*^+^ subpopulation was found. This small subpopulation accounted for only 0.25 ± 0.15% ADSVF cells (Fig. 4, fig. S2, and note S2).

Some similarities were found in B, plasma, and mast cells detected in both tissues. Specifically, plasma cells were the only populations transcriptionally identical enough to cluster on top of one another in UMAP (Fig. 2A), but these *MZB1*-expressing cells accounted for only 0.13 ± 0.14% ADSVF and 2.11 ± 2.02% BMAC cells (tables S2 and S3). Two *MS4A1*-expressing B cell populations accounted for 4.57 ± 3.38% in BMAC, one of which was similar to the 0.16 ± 0.22% small population in ADSVF (tables S2 and S3 and notes S1 and S2).

Apart from distinct transcriptional profiles of the cell types detected in both tissues, there were global differences between BMAC and ADSVF. For instance, more than half of the cells transplanted as ADSVF represent seven transcriptionally distinct fibroblasts/fibroadipogenic progenitors (53.46 ± 12.74%), and these subpopulations were not present in BMAC. Another 16.35 ± 8.91% included a total of 15 transcriptionally distinguishable endothelial cell clusters that were not present in BMAC, and 4 clusters of macrophages were yet another major population characteristic of ADSVF (10.81 ± 4.66%) but not BMAC (Fig. 2A and tables S2 and S3). Detailed cell frequencies and transcriptional profiles of these and other cells found in ADSVF are described in the note S2. Conversely, many populations including hematopoietic stem cells (HSCs), granulocyte progenitors, eosinophils, basophils, neutrophils, thrombocytes, megakaryocyte progenitors, and erythroblasts were unique to BMAC. Their detailed frequencies, subpopulations, and DEG patterns are described in note S1. It was interesting that fibroblasts and endothelial cells were not detected in BMAC. We speculate that they may have gotten removed before centrifugation as the marrow aspirate is passed through a filter to remove bone spicules to avoid clogging of the centrifuge tubing.

### Proteins administered as therapeutic BMAC and adipose are different

**Fig. 5.**
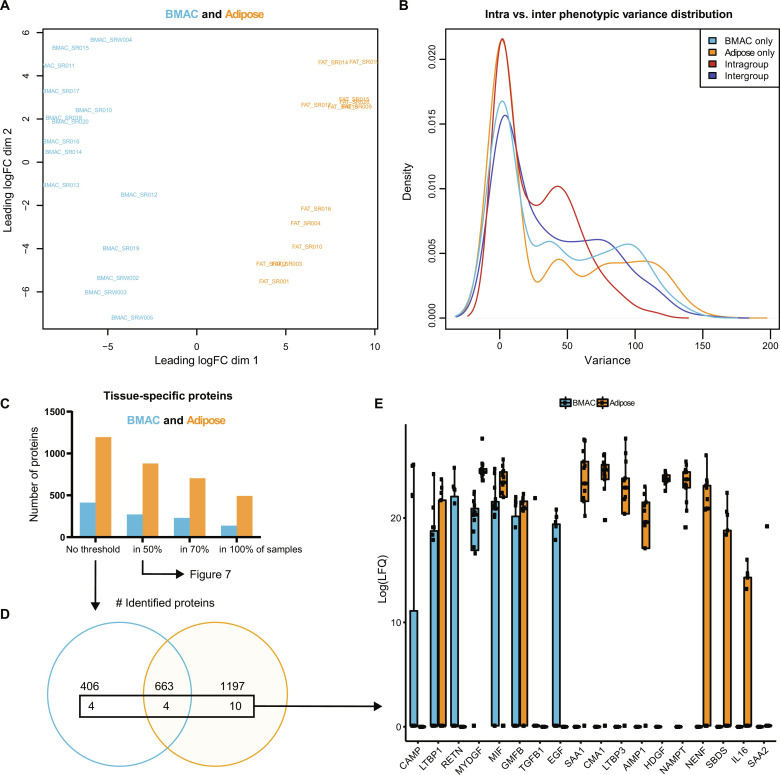
Proteomics analysis of BMAC and adipose. (**A**) Multidimensional scaling (MDS) plot. (**B**) Variance distribution plot. (**C**) Number of proteins identified in BMAC and adipose. (**D**) Venn diagram illustrating the overlap between tissue types. The black box indicates the number of detected cytokines, growth factors, and other proteins with immunomodulatory function. (**E**) Relative abundance of these proteins. Values are medians, boxes are first and third quartiles, bars are 95% confidence intervals. *n* = 15 BMAC and 13 adipose preparations. FC, fold change; LFQ, label-free quantification intensity.

**Fig. 6.**
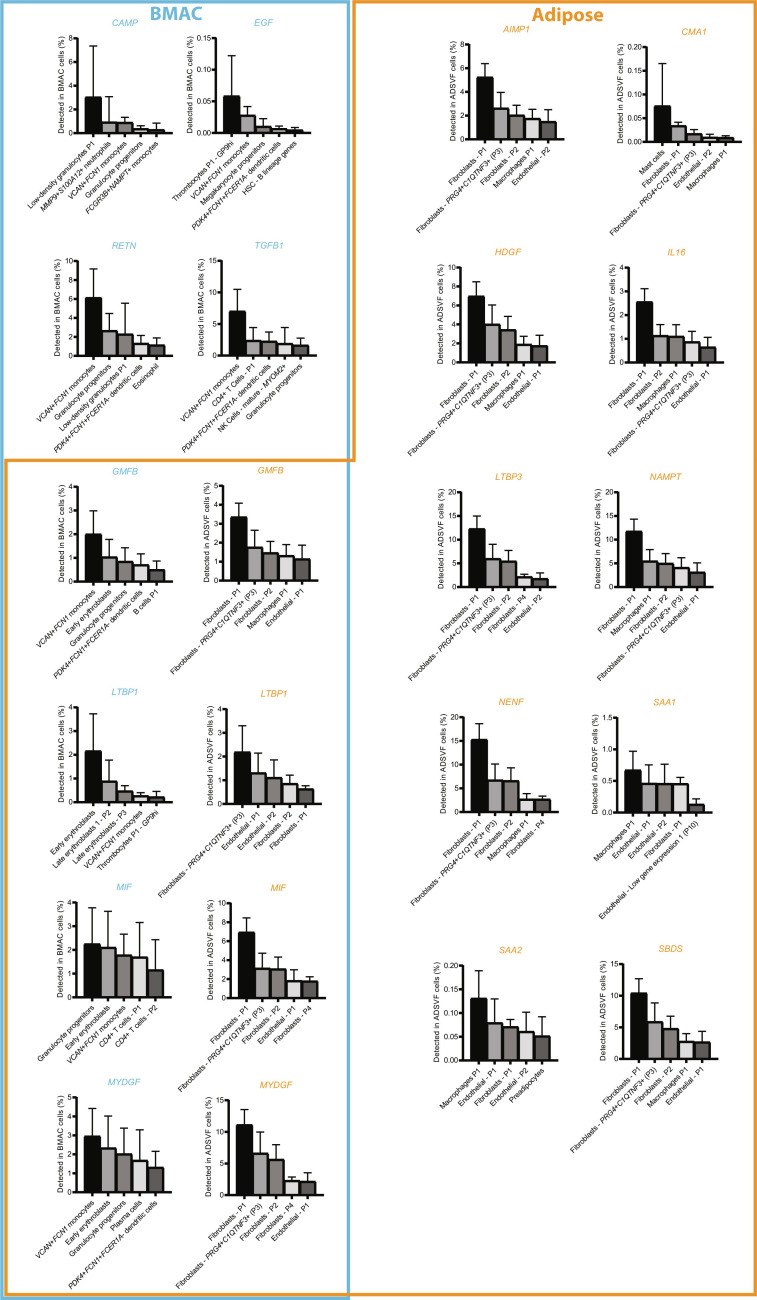
Cell fractions expressing the transcripts for immunomodulatory proteins from Fig. 5D. Values are means ± SD of % BMAC and ADSVF cell, respectively. The top 5 populations are shown per transcript. The full hierarchical list of populations can be found in table S11. *n* = 13 BMAC, 11 ADSVF.

In addition to regenerative cell populations, part of the therapeutic potential has been attributed to growth factors, cytokines, and other proteins present in BMAC and adipose preparations (*15*, *43*, *80*, *81*). Thus, unbiased label-free mass spectrometry was performed to compare the proteins between these tissues, and notable heterogeneity was found (Fig. 5 and tables S9 and S10). Unbiased multidimensional scaling resulted in a concentration of intertissue differences in dimension 1 and intratissue heterogeneity in dimension 2 (Fig. 5A). Intertissue variance exceeded intratissue variance and adipose preparations were more heterogeneous than BMAC (Fig. 5B). Four hundred ten of 1077 (38.1%) proteins were exclusively detected in BMAC, and 1207 of 1874 proteins (64.4%) were specific to adipose (Fig. 5, C and D). To identify potentially regenerative proteins, these results were compared with the curated list of 452 growth factors, cytokines, and other proteins with documented immunomodulatory function from the Molecular Signatures Database MSigDB (*82*). Of those proteins, CAMP, RETN, TGFB1, and EGF were detected in BMAC only, LTBP1, MYDGF, MIF, GMFB were detected in both tissues, and SAA1, CMA1, LTBP3, AIMP1, HDGF, NAMPT, NENF, SBDS, IL-16, and SAA2 were detected in adipose only (Fig. 5E). To identify the cell populations transcribing—and thus potentially secreting—these immunomodulatory proteins upon transplantation, corresponding transcripts were located in the single-cell atlas, and the percentage of positive cells per all BMAC and ADSVF cells was fractioned per cell population (Fig. 6). Expectedly, the top five cell populations contributing to each sample’s proteome were large populations, such as monocytes, granulocyte, and erythrocyte lineage cells in BMAC and fibroblasts, macrophages, and endothelial cells in ADSVF (Fig. 6 and table S11). BMAC-MSCs and ADSVF-pericytes played a very minor role, in part because their populations were so much smaller. Interleukin 6 (IL-6), tumor necrosis factor–α (TNFA), vascular endothelial growth factor (VEGF), hepatocyte growth factor (HGF), leukemia inhibitory factor (LIF), KIT, insulin-like growth factor 1 (IGF-1), and other growth factors and cytokines typically associated with the regenerative secretome of BMAC, ADSVF, and MSCs specifically (*65*, *80*, *83*, *84*) were either not present or not concentrated enough to be detected by unbiased label-free mass spectrometry.

**Fig. 7.**
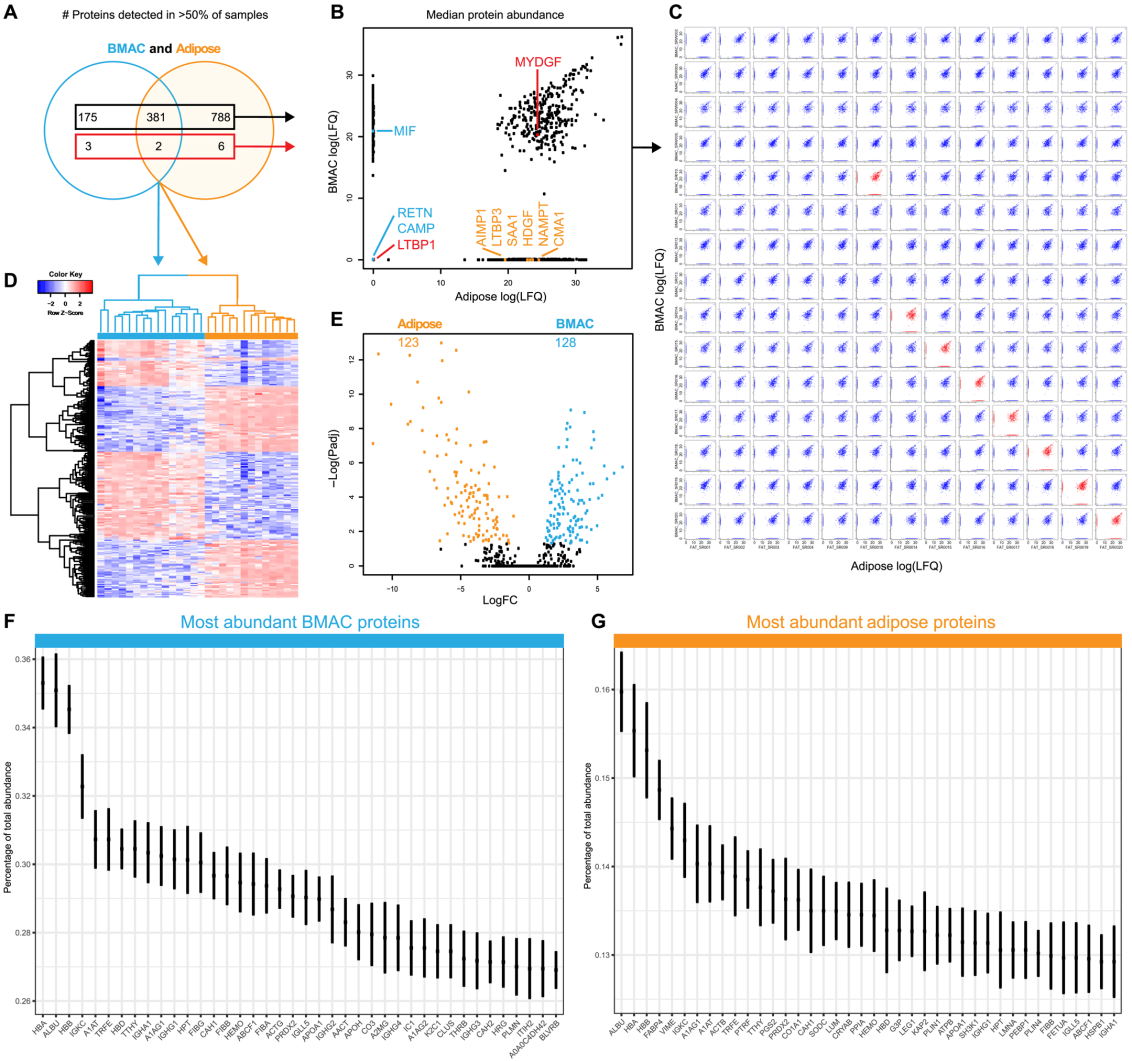
Quantitative comparison of proteins detected in at least 50% of BMAC and adipose preparations. (**A**) Venn diagram showing the overlap of cytokines, growth factors, and other proteins with immunomodulatory function (red box) and all other proteins (black box). (**B**) Scatter plot comparing relative protein quantities from (A). Values are medians. (**C**) Pairwise quantitative comparison from (B). Red scatter plots compare preparations from the same participants. (**D**) Heatmap of differentially detected proteins. (**E**) Volcano plot showing medians of proteins quantified in both BMAC and adipose. (**F** and **G**) Top 40 most abundant proteins in clinical-grade BMAC and adipose. Values are medians and 95% confidence intervals. *n* = 15 BMAC and 13 adipose preparations. FC, fold change; Padj, adjusted *P* value.

For a quantitative comparison between tissues, low-abundance proteins (detected in less than 50% of samples per tissue) were removed, which decreased the proteins unique to BMAC to 31.7% (178 of 561 proteins) and increased the unique proteins in ADSVF to 67.5% (794 of 1177 proteins) (Fig. 7A). Of the potentially regenerative proteins identified from MSigDB above, MYDGF was the only one detected at a high frequency in both tissues (Fig. 7B). Quantitative comparison of proteins between tissues from the same individuals did not result in a different scatter profile compared with random pairwise comparison (Fig. 7C). Of the 383 quantified proteins present in both sample types, 251 were present at different concentrations and separated the tissue types in an unbiased heatmap (Fig. 7D), 128 (33%) were more abundant in BMAC, and 123 (32%) were higher concentrated in ADSVF (Fig. 7E). Many of the most abundant proteins appeared to be blood derived and included albumin, hemoglobin, immunoglobulins, and others. Adipose-related proteins like fatty acid–binding protein and perilipin 1 (PLIN1) were highly abundant in adipose (Fig. 7, F and G). In summary, there is very little overlap between cell populations and proteins injected as BMAC or adipose preparations, and 66% of the proteins detected in both tissues are injected at notably different doses.

## DISCUSSION

We compared the two most common clinical cell preparations obtained from the same individuals to define and contrast their cellular and protein compositions. This dataset provides detailed quantification of 62 BMAC and 57 ADSVF cell (sub)populations, their individual single-cell transcriptomes, and the proteome of clinical-grade BMAC and adipose preparations. Furthermore, we report surface marker genes per population with the intention to provide the scientific community with a guide of potential surface markers to improve the isolation of a specific subpopulation for detailed downstream analysis and to create engineering opportunities. We then used this new atlas to investigate MSCs along with secreted cytokines and growth factors. Last, a *C1QB*^+^ population that shares a unique transcriptional signature with MSCs was found in BMAC. This atlas further revealed substantial heterogeneity within cell populations and between tissues, which can be interactively explored on http://muscle.ucsd.edu/BMACandADSVF.

Both BMAC and ADSVF have been interchangeably marketed as stem cell–based treatments for a variety of diseases (*2*, *13*, *15*, *81*, *85*, *86*). This new atlas challenges this one-cell-cures-all-diseases paradigm because: First, the cell populations marketed under the stem cell umbrella were transcriptionally unrelated with one another; i.e., there was no “one therapeutic cell” present in both tissues. This does not preclude one or more populations (or their interaction) from having regenerative potential in certain patient populations, but it motivates careful use of the stem cell terminology and marketing across different transplantable tissues and conditions. Second, MSC concentration in BMAC (even after complete RBC digestion) was so low that it first must be proven to be a therapeutically relevant dose, especially with regard to the >99% other cells transplanted as BMAC. In ADSVF, we identified controversy over *MCAM*^+^ pericytes versus *CD34*^+^ fibroblastic/fibroadipogenic cells as both of them have been marketed as therapeutic cells, possibly because both populations exhibit equivalent “MSC characteristics” in culture differentiation assays (*53*, *59*, *87*, *88*). To address this issue, cell fate and functional data upon transplantation of noncultured cells are needed to define these populations by their in vivo therapeutic function rather than in vitro phenotype. Overall, these data reinforced a previous note that therapeutic function of BMAC and ADSVF remains to be established after transplantation in human, along with mechanisms of action and dose-response curves (*4*, *34*, *89*). Given the little overlap between cells administered, the mechanism of action must be assumed to be entirely different between BMAC and adipose, should there be clinical benefit in any setting. Currently, 61% of clinical experts recommend the rigorous use of the ISCT guidelines (*44*) as a standard to define MSCs in clinical tissues (*43*). Contrasting this recommendation, our current data show that the “standard flow cytometry markers” fail to quantify fresh MSCs in the clinically most used cell preparations. This suggests that these markers are either not cell identity defining and change depending on the culture assay or the cell identity changes. In either case, our data suggest that ISCT guidelines are limited to culture-expanded cells, even though this was never specified by ISCT/IFATS (*44*, *90*). Guidelines for ADSVF cells, specifically, were explicitly intended for use in both uncultured and cultured populations (*59*). These results should also be considered in the ongoing discussion related to nomenclature as it appears incorrect to collectively name cultured and noncultured cells derived from different tissues as stem cells or MSCs. Furthermore, the lack of marker retention before/after culture (or a culture-induced change of cell identity) supports the current regulatory environment in the United States, where the Food and Drug Administration (FDA) restricts transplantations to minimally manipulated tissues (i.e., fresh, uncultured). Culture-expanded MSCs or other more than minimally manipulated cells and procedures (e.g., enzymatically released from solid tissues, FACS-sorted) may only be permitted under an investigational new drug application as an FDA-approved clinical trial (*91*). Many MSC quantification strategies established in cultured cells appear to fail to identify fresh MSCs (*34*), suggesting that a new clinically applicable quantification strategy is needed to enable dose-response relationships. This atlas suggested LEPR to be a reliable transcriptional and surface protein marker for BMAC-MSCs which can also be used for FACS-sorting these cells. In mice, LEPR^+^ stromal cells have already been shown to form CFUs in culture, to be able to differentiate into adipocytes, chondrocytes, and osteoblasts in vitro, and to form bone and adipocytes in vivo after irradiation or fracture (*92*). Furthermore, LEPR-expressing cells have been shown to be involved in marrow homeostasis as well as neural and vascular regeneration after myeloablation (*93*), and they promote myeloid and erythroid progenitor maintenance during early postnatal development (*94*). Conversely, in ADSVF, a similar MSC did not appear to exist. The current single-cell analysis suggested that the *CD34^+^* ASC and fibroblasts are in fact the same cell. This finding is supported by previous studies showing that these cell types appear to be indistinguishable by flow cytometry, transcriptional analysis, and functional characterization (*95*–*97*), but this still needs clarification through thorough in vivo transplantation assays, as done previously for stem cells in bone (*62*, *98*).

The therapeutic potential of BMAC and ADSVF has further been attributed to cytokines, growth factors, and other immunomodulatory proteins already present or secreted by MSCs after injection (*8*, *80*, *99*). From a clinical point of view, it is still unknown which BMAC/ADSVF proteins potentially exert a therapeutic effect and what the minimally required dose would be to induce disease-modifying activity. The current dataset demonstrated the proteome of these cell preparations to be very different, and many proteins that have historically been associated with a regenerative function, such as TNFA, VEGF, IGF-1, or IL-6 (*65*, *83*, *84*), were either absent or in such low concentration that they could not be detected by unbiased mass spectrometry. Thus, the following clinical questions remain regarding therapeutic proteins: (i) Is a potentially regenerative protein at such a low dose exerting a clinically relevant effect, especially in the face of the >1000 other proteins being transplanted at a much higher concentration? (ii) If there are improvements exceeding the MCID (minimal clinically important difference) (*100*), were the therapeutic effects in fact induced by a different, more abundant protein? (iii) Should the translational field move away from BMAC and ADSVF to a drug that presents with a known/higher therapeutic concentration of the proposed active ingredient? The latter appears urgent to address because patients, conditions, and severities treated with biologics are very heterogeneous, i.e., the underlying interindividual (in)ability of a tissue to respond to a nonstandardized therapy is naturally leading to heterogeneous outcomes that are challenging to decipher scientifically (*42*). Understanding the biological “state” of a tissue, i.e., its capacity to respond to a well-defined therapeutic stimulus may offer a more personalized medicine approach or at least a more rigorous scientific approach to establish mechanisms of action and investigate dose-response curves. To do so, the current resource provides tools to standardize and calculate doses of specific BMAC and ADSVF ingredients.

This atlas of paired clinical BMAC and ADSVF preparations challenged the current one-stem-cell-cures-all-diseases paradigm and is a rich comparative resource for future discovery, especially in light of these two clinical tissues that are administered to patients frequently and for the exact same purpose. Its in-depth classification of transcriptionally different subpopulations and cell states, and the discovery tool for novel surface marker genes to isolate a specific population allows detailed functional characterization and offers engineering opportunities. This is demonstrated by the transcriptional analysis and comparison of BMAC-MSCs with ASCs/fibroblasts, and the unbiased discovery of LEPR as a reliable marker for BMAC-MSCs, which poses a new translational and preclinical opportunity because LEPR has previously been identified in murine MSCs, too (*92*).

This study has several limitations. It was performed in a specific patient demographic for which cell injections are often considered; however, it is possible that a different target demographic, e.g., athletes, presents with different cellular and protein composition. It is also possible that a different harvesting technique, harvesting location, and tissue processing technique would yield different results. In this regard, it may also be interesting to compare results to tissues before concentration as well as after culture expansion. Cluster annotation was based on an extensive and detailed review of previously published transcriptional datasets of human noncultured cells and some of these annotations conflicted with the physical distances on the UMAP plots and/or with the proposed transcriptional relation of clusters in the unbiased dendrograms. This was due to displaying a high-dimensional dataset in only two dimensions and examples include the following: (i), in Fig. 2B where a BMAC-derived granulocyte started to cluster crosses the T/NK cell cluster upon adding more heterogeneity (here, cultured MSCs), (ii), populations that the algorithm put in one cluster (for example the *C1QB*^+^*APOE*^+^ population) but upon subclustering them into three subpopulations, each of the three subpopulations was transcriptionally more related to other populations rather than to the two other subpopulations from the original cluster they were derived (fig. S3), and (iii), in some occasions, subpopulations were related such as ADSVF-endothelial cells P15 with P11 (fig. S3) but upon removal of the BMAC dataset, the endothelial cell P15 population was largely unrelated to any other population in ADSVF (fig. S2). A second limitation was that populations were split into subpopulations based on transcriptional differences but for some of these subpopulations it remains to be proven whether their subclassification is indeed biologically relevant rather than presenting a transient transcriptional state at the time of measurement. Both limitations require functional verification of the proposed 62 and 57 transcriptionally identified cell populations in the context of potentially therapeutic activity. Similar functional profiling is needed for populations that have been less well characterized in situ, for example the BMAC cluster annotated as MSCs and the undefined *C1QB*^+^ cluster, or the adipose-derived fibroblasts/ASCs/pericytes. We aimed to facilitate this in future studies by the transcriptional analysis of differentially expressed surface markers. Furthermore, our analysis revealed several small subpopulations and rare cell types, which have an increased risk of being undersampled for detailed analysis. When rare populations are investigated in more detail in future, the current data should be considered for power analysis to avoid undersampling. Another limitation was the requirement of enzymatic digestion to create cell suspensions required for flow cytometry and scRNA-seq. Enzymatic digestion is “more than minimal manipulation” as regulated by the FDA. However, even though no adipose-derived cell suspensions are currently legal to be directly used clinically (unless under an investigational new drug application), these are the cells that are contained within a minimally manipulated microfat transplantation. Conversely, the proteomics data were derived from a minimally manipulated fraction of the sample. Last, our mass spectrometry data may present with a false-negative rate for low abundant proteins since their detection may have been outcompeted by high abundant proteins, such as albumin. We consciously decided to accept this limitation and not remove albumin because a critical goal of this study was to quantify what is being injected into patients; thus, it was essential to measure these preparations in the form they are used clinically.

In conclusion, these data suggest that cell preparations derived from the most popular mesenchymal depots, BMAC and ADSVF, are predominantly a T cell/monocyte/erythroblast and a fibroblastic/endothelial cell injection, respectively. Furthermore, there are no transcriptionally comparable stem cells present in both clinically prepared injectates, nor do they appear to be related to culture-expanded MSCs, meaning that new guidelines and clinical gold standards are needed to define and quantify clinical-grade MSCs, ASCs, and other potentially therapeutic cells. Last, more human data are needed to establish in vivo function upon injection and to elucidate which patient population or tissue state would benefit from transplantation.

## MATERIALS AND METHODS

### Experimental model and participant details

This study was approved by the Institutional Review Board of the University of California San Diego. Informed consent was obtained from all participants. Nine female (mean ± SD; age: 64.2 ± 10.4 years; body mass: 69.6 ± 7.5 kg; height: 166.5 ± 6.9 cm; body mass index: 25.2 ± 2.7 kg m^−2^) and 12 male participants (57.3 ± 19.9 years; 92.5 ± 19.4 kg; 180.3 ± 7.5 cm; 28.3 ± 4.3 kg m^−2^) over the age of 18 and free of any hematologic disease agreed to donate both bone marrow and subcutaneous adipose tissue for this study. Two female participants self-identified as “non-white/mixed” race, one male participant self-identified as “American Indian/Alaskan Native”, all other participants self-identified as “white” (Table 1). Study participants were recruited from STB’s practice in the Department of Orthopaedic Surgery at University of California, San Diego (UCSD) and from J.L.C.’s practice in the Department of Anesthesiology at UCSD, and tissues were harvested during standard-of-care autologous cell injections or total hip arthroplasty. Not all analyses could always be run for all 21 participants because it was a tremendous logistical effort to consent patients, collect the tissues during surgery, simultaneously prepare tissues for flow cytometry and scRNA-seq and run those assays, and conserve the tissue for mass spectrometry; everything on the same day so that no cells had to be cultured/preserved with the goal to measure these tissues “as similar to injected” as possible. The datasets per participant are summarized in Table 2.

**Table 1.** Participant characteristics. m, male; f, female.

Sample ID	Gender	Age at surgery date	Body mass (kg)	Height (cm)	BMI(kg m^−2^)	Race	Ethnicity	Comments
SR001	m	76	103.9	175.3	33.8	White	Non-Hispanic	Total hip arthroplasty
SR002	m	57	95.3	188	27.0	White	Non-Hispanic	Total hip arthroplasty
SR008	m	57	94.8	185.4	27.6	White	Non-Hispanic	Total hip arthroplasty
SR010	m	67	98.9	177.8	31.3	White	Non-Hispanic	Total hip arthroplasty
SR013	m	71	95.3	182.9	28.5	White	Non-Hispanic	Total hip arthroplasty
SR014	m	80	103.4	180.3	31.8	White	Non-Hispanic	Total hip arthroplasty
SR016	m	41	84.1	170.2	29.0	American Indian/Alaskan Native	Non-Hispanic	Total hip arthroplasty
SR018	m	59	72.8	177.8	23.0	White	Non-Hispanic	Total hip arthroplasty
SRW002	m	82	80.7	177.8	25.5	White	Non-Hispanic	BMAC injection to left sacroiliac joint, left supraspinatus tendon, and left glenohumeral joint
SRW003	m	20	61.2	172.7	20.5	White	Non-Hispanic	BMAC injection to right supraspinatus tendon, infraspinatus tendon, subscapularis muscle, and long head biceps tendon
SRW004	m	28	138.5	198.1	35.3	White	Non-Hispanic	BMAC injection to both knee joints, right LCL tear, right patellar tendon
SRW005	m	49	81.6	177.8	25.8	White	Non-Hispanic	BMAC injection to left knee joint
Count	12							
Mean		57.3	92.5	180.3	28.3			
SD		19.9	19.4	7.5	4.3			
SR003	f	69	69.4	170.2	24.0	White	Non-Hispanic	Total hip arthroplasty
SR004	f	77	65.8	160	25.7	White	Non-Hispanic	Total hip arthroplasty
SR009	f	72	85.7	167.6	30.5	White	Non-Hispanic	Total hip arthroplasty
SR011	f	66	58.3	159.6	22.9	Other/Mixed	Non-Hispanic	Total hip arthroplasty
SR012	f	58	71	172.7	23.8	White	Non-Hispanic	Total hip arthroplasty
SR015	f	50	74.4	172.7	24.9	White	Non-Hispanic	Total hip arthroplasty
SR017	f	66	68.3	162	26.0	White	Non-Hispanic	Total hip arthroplasty
SR019	f	47	65.8	176.5	21.1	White	Non-Hispanic	Total hip arthroplasty. Acetabular cyst found, which did not affect marrow aspiration
SR020	f	73	68	157.5	27.4	Other/Mixed	Non-Hispanic	Total hip arthroplasty
Count	9							
Mean		64.2	69.6	166.5	25.2			
SD		10.4	7.5	6.9	2.7			

**Table 2.** Overview of BMAC and adipose datasets. A, Arthrex; E, EmCyte.

Sample ID	BMAC					Adipose			
	**Concentration system**	**Flow cytometry**	**scRNA-seq**	**LEPR+ FACS and scRNA-seq**	**Proteomics**	**Flow cytometry**	**scRNA-seq**	**LEPR+ FACS and scRNA-seq**	**Proteomics**
SR001									✓
SR002									✓
SR003	A		✓						✓
SR004									✓
SR008	A	✓	✓				✓		
SR009	A	✓	✓				✓		✓
SR010	A	✓	✓		✓	✓	✓		✓
SR011	A	✓	✓		✓	✓	✓		
SR012	A	✓	✓		✓	✓	✓		
SR013	A	✓	✓		✓	✓	✓		
SR014	A	✓	✓		✓	✓	✓		✓
SR015	A	✓	✓		✓	✓			✓
SR016	A				✓	✓			✓
SR017	A	✓	✓		✓	✓	✓		✓
SR018	A	✓	✓	✓	✓	✓	✓		✓
SR019	A	✓	✓	✓	✓	✓	✓		✓
SR020	A	✓	✓		✓	✓	✓		✓
SRW002	E	✓			✓				
SRW003	E	✓			✓				
SRW004	E	✓			✓				
SRW005	E	✓			✓				
Count		16	13	2	15	11	11		13

### Method details

#### Surgical procedure and sample preparation

After skin incision for total hip arthroplasty using the posterior approach, a subcutaneous adipose biopsy of approximately 5 g was taken from the proximal posterolateral thigh region using a scalpel. After removal of the femoral head and exposure of the acetabulum, a pilot hole was drilled into the superior dome of the acetabulum using a 2.0 Steinmann pin as established previously (*101*). A trocar with needle (Arthrex, Angel BMC ABS-10062) was inserted and forwarded to a depth of 1.5 cm using a mallet. After removing the needle, 2× 30-ml syringes preloaded with 5 ml of Anticoagulant Citrate Dextrose solution A (Arthrex, Angel BMC ABS-10062) were attached to the trocar, and bone marrow was aspirated from a single aspiration hole to a total volume of 60 ml. The entire volume was loaded onto the Angel centrifuge (Arthrex, ABS-10060) and spun at a hematocrit setting of 7% which resulted in approximately 2 to 3 ml of clinical-grade, ready-to-inject BMAC. In patients qualifying for minimally invasive cell therapy, the same volume of bone marrow was aspirated from the posterior iliac crest using the Jamshidi device needle (Ranfac Corporation, Avon, MA, USA) and concentrated to BMAC using the EmCyte PureBMC 60-ml system (EmCyte Corp, Fort Myers, FL, USA) according to the manufacturer’s protocol. One milliliter was used for research, the remaining volume was used for patient care. To generate ADSVF, adipose was rinsed in Dulbecco’s phosphate-buffered saline (DPBS), minced, and incubated in 45 ml of enzyme mix containing low-glucose Dulbecco’s modified Eagle’s medium (DMEM), 10% fetal bovine serum (FBS), 1% penicillin/streptomycin, 90 U of dispase, and 2250 SI at 37°C under rotation for 1 hour. Then, remaining debris was removed using a 70-μm cell strainer, and the enzyme mix was diluted 1:1 with DMEM containing 10% FBS and 1% penicillin/streptomycin, and aspirated after centrifugation (400*g* for 5 min). To remove RBCs for flow cytometry and scRNA-seq, pellets of both ADSVF and BMAC were gently resuspended in ACK buffer (Thermo Fisher Scientific, A1049201) and incubated for 5 min at room temperature. Passage 2 cultured MSCs were purchased from Lonza as “marrow-derived mesenchymal stem cells” (#PT-2501). The donor was 24 years old and identified as a black female. The donor tested negative for HIV, HBV, and HCV and cells were free of contamination including mycoplasma. Cells passed adipogenic, osteogenic, and chondrogenic differentiation and presented with the following flow cytometry profile: 100% CD105^+^, 96% CD166^+^, 100% CD44^+^, 100% CD90^+^, 96% CD73^+^, and no detection of CD14, CD34, CD45, HLA-DR, and CD19. Cells were thawed and cultured in low-glucose DMEM, 10% FBS, and 1% penicillin/streptomycin. At 70% confluency, MSCs were trypsinized and subjected to scRNA-seq.

#### Flow cytometry

Cells were incubated in DPBS containing 2.5% FBS and the following antibodies for 30 min on ice: BMAC: CD14-FITC (BD Bio 561712), CD19-FITC (BD Bio 560994), CD45-FITC (BD Bio 560976), HLA-DR–FITC (BD Bio 560944), CD34-PE-CF594 (BD Bio 562383), CD73-PerCP-e710 (eBioscience 46-0739-41), CD90-PE-Cy7 (BD Bio 561558), CD105-BV421 (BD Bio 566265) (*44*). ADSVF: CD31-APC-Cy7 (BD Bio 563653), CD34-PE-CF594 (BD Bio 562383), CD45-BV711 (BD Bio 564358), CD235a-PE-Cy7 (BD Bio 563666), CD73-FITC (BD Bio 561254), CD90-APC (BD Bio 561971), CD105-BV421 (BD Bio 566265) (*59*). After washing, dead cells were stained using the Fixable viability dye eFluor 506 (eBioscience 50246097) in DPBS for another 30 min on ice. After washing, the pellet was resuspended in pH-adjusted (7.4) DPBS containing 2.5% FBS and 1 mM EDTA, and run through a 40-mm cell strainer cap into a 5-ml tube. Surface protein expression was assessed on a ZE5 flow cytometer (BioRad). Compensation was established using single stain beads and, if necessary, manually adjusted in the FlowJo software (V.10.6.1, BD Biosciences). Gates were set in FlowJo using Fluorescence Minus One (FMO) stains. To isolate LEPR-expressing cells, the protocol was similar, but the antibody was CD295-AF647 (BD Bio 564376), and cells were sorted on a FACSAria II (BD Bio) and gated in FACSDiva v6.1.3 (BD Bio) using an FMO.

#### Single-cell RNA sequencing

BMAC, ADSVF, and cultured MSCs single-cell suspensions in DPBS containing 0.04% bovine serum albumin were prepared at a concentration of 1000 cells μl^−1^, loaded onto the Chromium controller (10x Genomics), and scRNA-seq libriaries were prepared using the Chromium Single Cell 3′ v3 kits (10x Genomics) according to the user manual (10x Genomics, CG000183 Rev. A). Libraries were sequenced using Paired End reads, with a Read 1 of 28 basepairs and a Read 2 of 98 basepairs, on a HiSeq 4000 (Illumina). One ADSVF sample did not pass initial quality screening and was removed from further processing (table S1).

#### Cluster annotation

After unbiased clustering and visualization, cell populations were annotated manually using previously published marker genes and human transcriptional datasets. BMAC cells were identified using the following marker genes: MSCs by *PDGFRA*, *NGFR*, and *THY1* (*74*, *102*–*104*). T cells were subclassified as CD4^+^ and CD8A^+^ populations (*75*–*77*), and subclusters were compared with the detailed subclassification by Szabo and colleagues and, if applicable, named similarly (*77*). *CD4*^+^ T_regs_ were identified by *CTLA4*, *FOXP3*, and *IL2RA* (*77*, *105*). *CD8*^+^*HLA*^+^ T_regs_ were identified based on their expression of *HLA* genes and *HLA*-associated *CD74* (*106*, *107*). *CD8*^+^ “cytotoxic” T cells were identified by high expression of *CCL5*, *NKG7*, *ZEB2*, and *KLRG1*, *CD8*^+^ “cytokine” T cells were identified by high expression of *CCL3*, *CCL4*, and *XCL2*, and CD8^+^ “resting effector memory” T cells were identified by *KLRB1* and *JAML* (*77*). NK cells were identified by strong *GNLY* and *NKG7* expression, and if applicable, subclassifications from Yang and colleagues were adapted (*78*). These included the CD56^+^ population identified by *NCAM1* and *XCL1*, adaptive NK cells by differentially expressed *CD3E*, *CCL5*, *IL32*, and *CD52*, and mature NK cells by pronounced expression of *GZMB* and *PRF1* (*78*). Monocytes were identified as *VCAN*^+^*FCN1*^+^, *FCGR3A*^+^, and *FCGR3B*^+^*NAMPT*^+^ subpopulations in accordance with Villani and colleagues’ Mono1, Mono2, and Mono3 populations (*79*). Dendritic cells were identified by their expression of *HLA* genes and *CLEC9A*^+^, *FCER1A*^+^*CD1C*^+^, and *GZMB*^+^*ILR8*^+^ (plasmacytoid) subpopulations, respectively, which were in accordance with Villani and colleagues’ DC1, DC2, and DC6 populations (*79*). Pre-*FCER1A*^+^*CD1C*^+^ and pre-plasmacytoid subpopulations were annotated on the basis of their UMAP clustering profile creating a fork between their well-defined mature states (fig. S1) and on the basis ofdetection of *SPATS2L* and *CTSV* (*76*). HSCs were identified by *CRHBP* and *AVP* (*76*, *108*) and distinguished into “native” by *CD164*^high^ and absence of lineage genes, into monocyte lineage by *CD14*, into T lineage by *CD3D*, *CD3E*, and *IL7R*, and into B lineage by *IGLL1* and *HELLS* (*75*–*77*, *108*). *CD34*^+^ early erythroblast progenitors clustered separately from HSCs and were identified by *CD34*, *CSF2RB*, *PDZD8*, *AC084033.3*, and *EMID1* (*76*, *108*). Further development into erythroblast progenitors and early/intermediate/late erythroblast stages was determined by loss of *CD34* and gradually increasing expression of *HBA1/2*, *HBB*, *HBD*, *AHSP*, and *GYPA* (*75*, *76*, *108*). Megakaryocyte progenitors were identified using *VWF*, *SELP*, and *ITGA2B* (*76*, *108*, *109*). Granulocyte progenitors were identified using *PRTN3*, *ELANE*, *MPO*, and *AZU1* (*75*, *76*, *108*). The two low-density granulocyte populations were identified using *LTF*, *LCN2*, *OLFM3*, and *CRISP3* (*110*). Two distinct neutrophil populations were identified using *MMP9*, *PGLYRP1*, and *DEFA1/3* (*110*–*112*). Eosinophils were identified using *LYZ*, *LGALS1*, *AC020656.1*, and *RETN* (*76*, *113*). The basophil/mast cell population was identified by their pronounced *TPSAB1* and *TPSB2* expression (*76*, *114*). Plasma cells were identified using *MZB1*, *JCHAIN*, *IGKC*, and *IGHA1* (*75*, *76*, *115*) and preplasma cells using *DNTT* and *VPREB1* (*76*). B cells were annotated using *MS4A1*, *BANK1*, and *CD79A* (*75*, *76*, *116*) and pre B cells were identified using *CD79B*, *TCL1A*, and, *PCDH9* (*76*). Last, thrombocytes were identified using *PPBP*, *GP9*, *PF4*, and *CAVIN2* (*76*, *117*).

The following marker genes were used to identify cells in ADSVF: B cells were annotated using *BANK1*, *MS4A1*, *CD79A*, *CD52*, and *TCF4* (*75*, *76*, *116*). T cells were subclassified as above. In addition, *CD8*^+^ effector memory T cells were annotated based on *PRF1* and *KLRB1* detection and another *CD8*^+^ subpopulation was named “activated” based on *NKG7*, *GNLY*, and *GZMH* (*77*). NK cells were identified as described above. Monocytes were identified as described above and the two subpopulations found corresponded to Villani *et al.*’s Mono1 (*CD14*^+^*VCAN*^+^) and Mono3 (*FCGR3B*^+^*G0S2*^+^) subpopulations (*79*). Similar to BMAC, dendritic cells were identified using the atlas by (*79*) and included *CLEC9A*^+^ (“DC1”), *CD1C*^+^*FCER1A* (“DC2”), *CD1C*^+^*FCER1A*^+^*S100A9*^+^ (“DC3”), and *CD1C*-*THBD*- (“DC4”). Macrophage clusters were identified using *CD14*, *CD68*, *CD163*, *MRC1*, and *LYVE1* (*118*, *119*). Endothelial cells were identified using *PECAM1*, *VWF*, and *CDH5* (*120*–*123*). Pericytes were identified using *MCAM*, *RGS5*, and *STEAP4* while lacking the endothelial markers (*53*, *124*, *125*). Smooth muscle cells were identified using *MYH11*, *MYOCD*, and *ACTA2* (*126*, *127*). Fibroblast clusters were identified using *DCN* and *PDGFRA* while lacking *PECAM1* and *PTPRC* (*124*). One fibroblast subpopulation was termed “hedgehog” on the basis of *HHIP*, *HHIP-AS1*, and *TSPAN8* expression (*128*, *129*). The myofibroblast population was identified by their distinct expression of genes involved in contractile properties including *ACTG2*, *CNN1*, *MYLK*, *TPM* as well as *TAGLN* and *ACTG2* genes which have been associated with fibroblast-myofibroblast trans-differentiation (*124*, *130*). Fibrocytes were identified by their expression of both fibroblastic and hematopoietic/monocyte/macrophage markers including *DCN*, *CD34*, *PDGFRA*, *PTPRC*, *CD14*, *CD68*, and *CD163* (*131*, *132*). The pre adipocytes cluster was named using adipocyte markers *PLIN1*, *CIDEA*, *CIDEC*, and *ADIPOQ* (*133*, *134*). Last, mast cells, erythroblasts, plasma cells, and proliferating cell clusters were identified using the same genes as described for BMAC above.

#### Sample preparation for mass spectrometry

Equal volume of 6 Molar Guanidine solution was added to 100 μl of collected tissue buffer and mixed. The samples were then boiled for 5 min followed by 5-min cooling at room temperature. The boiling and cooling cycle was repeated a total of 3 cycles. The proteins were precipitated with addition of methanol to final volume of 90% followed by vortex and centrifugation at maximum speed on a benchtop microfuge (14,000 rpm) for 10 min. The soluble fraction was removed by flipping the tube onto an absorbent surface and tapping to remove any liquid. The pellet was suspended in 200 μl of 8 M Urea made in 100 mM tris (pH 8.0). Tris (2-carboxyethyl) phosphine (TCEP) was added to final concentration of 10 mM, and chloro-acetamide solution was added to final concentration of 40 mM and vortex for 5 min. Three volumes of 50 mM tris (pH 8.0) was added to the sample to reduce the final urea concentration to 2 M. Trypsin was in 1:50 ratio of trypsin and incubated at 37°C for 12 hours. The solution was then acidified using TFA (0.5% TFA final concentration) and mixed. Samples were desalted using 100 mg of C18-StageTips as described by the manufacturer protocol. The peptide concentration of sample was measured using the bicinchoninic acid (BCA) assay after resuspension in sample loading buffer and the total of 1 μg is injected for each label-free quantification run.

#### Mass spectrometry

Trypsin-digested peptides were analyzed by ultrahigh-pressure liquid chromatography (UPLC) coupled with tandem mass spectroscopy using nano-spray ionization. The nanospray ionization experiments were performed using a Orbitrap fusion Lumos hybrid mass spectrometer (Thermo Fisher Scientific) interfaced with nano-scale reversed-phase UPLC (Thermo Dionex UltiMate 3000 RSLC nano System) using a 25-cm, 75-μm ID glass capillary packed with 1.7-μm C18 (*130*) BEHTM beads (Waters corporation). Peptides were eluted from the C18 column into the mass spectrometer using a linear gradient (5 to 80%) of ACN (acetonitrile) at a flow rate of 375 μl/min for 1 hour. The buffers used to create the ACN gradient were as follows: buffer A (98% H_2_O, 2% ACN, and 0.1% formic acid) and buffer B (100% ACN and 0.1% formic acid). Mass spectrometer parameters are as follows; an MS1 survey scan using the orbitrap detector [mass range (*m*/*z*): 400 to 1500 (using quadrupole isolation), 120,000 resolution setting, spray voltage of 2200 V, ion transfer tube temperature of 275°C, AGC target of 400,000, and maximum injection time of 50 ms] was followed by data dependent scans (top speed for most intense ions, with charge state set to only include +2 to 5 ions, and 5-s exclusion time, while selecting ions with minimal intensities of 50,000 at in which the collision event was carried out in the high-energy collision cell (HCD Collision Energy of 30%), and the fragment masses where analyzed in the ion trap mass analyzer (with ion trap scan rate of turbo, first mass *m*/*z* was 100, AGC Target 5000 and maximum injection time of 35 ms). Protein identification and label-free quantification was carried out using Peaks Studio 8.5 (Bioinformatics solutions Inc.).

### Quantification and statistical analysis

#### Bioinformatics processing and data visualization

The *count* function from Cell Ranger (v3.1.0) (https://support.10xgenomics.com/single-cell-gene-expression/software/pipelines/latest/what-is-cell-ranger) was used to align and quantify reads. Reads were aligned to GRCh38 (3.0.0; Ensembl release 93) provided by 10x Genomics (https://support.10xgenomics.com/single-cell-gene-expression/software/release-notes/build#grch38_3.0.0). The counts matrix was initially filtered to include genes that were expressed in greater than 0.1% of the cells and include cells that expressed at least 200 different genes. Low-quality cells were filtered out before analysis by removing outliers in cell features (<400 and >4000) and cells with high percentages of mitochondrial transcripts (>17%). After removing 13,778 low-quality cells in BMAC, and 11,588 low-quality cells in ADSVF, 50,836 BMAC and 40,129 ADSVF cells remained. The Seurat R package (*135*) was used for preprocessing, normalization, identifying variable features, scaling, linear dimensional reduction, clustering, and visualization of results. The Seurat FindMarkers function (Wilcoxon rank sum test) was used to calculate DEGs that defined each cluster. In BMAC, T and NK cells were further subclustered to investigate subtypes. Subclustering involved subsetting the cells of interest, recalculating variable features, scaling, dimensional reduction, clustering, and calculations of DEGs. In ADSVF, four populations—T/NK, monocytes/macrophages, fibroblasts, and endothelial cells—were subclustered separately to investigate transcriptional heterogeneity. Subclustering of fibroblasts and endothelial cells showed signs of batch effects. Thus, Seurat functions FindIntegrationAnchors and IntegrateData were used to integrate data based on sample ID before repeating the same methods outlined in the processing section.

#### Analysis of proteomic data

Label-free quantification values for each protein in each sample (filtered for log fold change >1 and significance >0) were used as input to the Perseus software package (*136*) which performed the following processing steps: filter out low-abundance proteins (detected in at least 50% of samples in each group), log transform remaining quantification values, impute values for proteins in samples with missing values using gaussian distribution with Perseus default parameters for distribution width and down shift, and perform a width adjustment normalization on each sample. In addition, the Perseus software was used to test for differentially abundant proteins between the two groups with a Student’s *t* test. This statistical test was followed by *P* value adjustment for multiple testing using the Benjamini-Hochberg method (*137*) within the R statistical computing environment (www.cran.org) with adjusted *P* value threshold of 0.05 used to identify significant proteins. Heatmaps were made with the heatmap.2 function from the gplots package for R (https://cran.r-project.org/web/packages/gplots/). Of additional importance in this study were proteins detected in only one sample group (in at least 50% of the samples) and not detected in the other group. To visualize these proteins in a scatter plot, a pseudosignal of 1.1 was assigned to all samples where the protein was undetected, thus producing a value close to 0 upon log transformation. The curated gene family list “cytokines and growth factors” was downloaded from the Molecular Signatures Database MSigDB (www.gsea-msigdb.org/gsea/msigdb/gene_families.jsp?ex=1) (*82*).

### Additional resources

#### Websites

muscle.ucsd.edu/BMACandADSVF.

## Supplementary Materials

### This PDF file includes:

Supplementary Notes S1 and S2 Figs. S1 to S6 Legends for tables S1 to S40 References
