# Supplementary material for: Comparative single-cell transcriptional and proteomic atlas of clinical-grade injectable mesenchymal source tissues
Source: Sci Adv. 2024 Jul 12;10(28):eadn2831. doi: 10.1126/sciadv.adn2831 (PMC11244553; doi:10.1126/sciadv.adn2831)
Supplement: Supplementary file 1 — Supplementary Notes S1 and S2 Figs. S1 to S6 Legends for tables S1 to S40 References [file sciadv.adn2831_sm.pdf]

Science Advances
AAAS

Supplementary Materials for

Comparative single-cell transcriptional and proteomic atlas of clinical-grade injectable mesenchymal source tissues

Severin Ruoss *et al.*

Corresponding author: Samuel R. Ward, s1ward@health.ucsd.edu

Sci. Adv. **10**, eadn2831 (2024)
DOI: 10.1126/sciadv.adn2831

The PDF file includes:

Figs. S1 to S6
Notes S1 and S2
Legends for tables S1 to S40
References

Other Supplementary Material for this manuscript includes the following:

Tables S1 to S40

Supplementary Material

Supplemental figures and legends

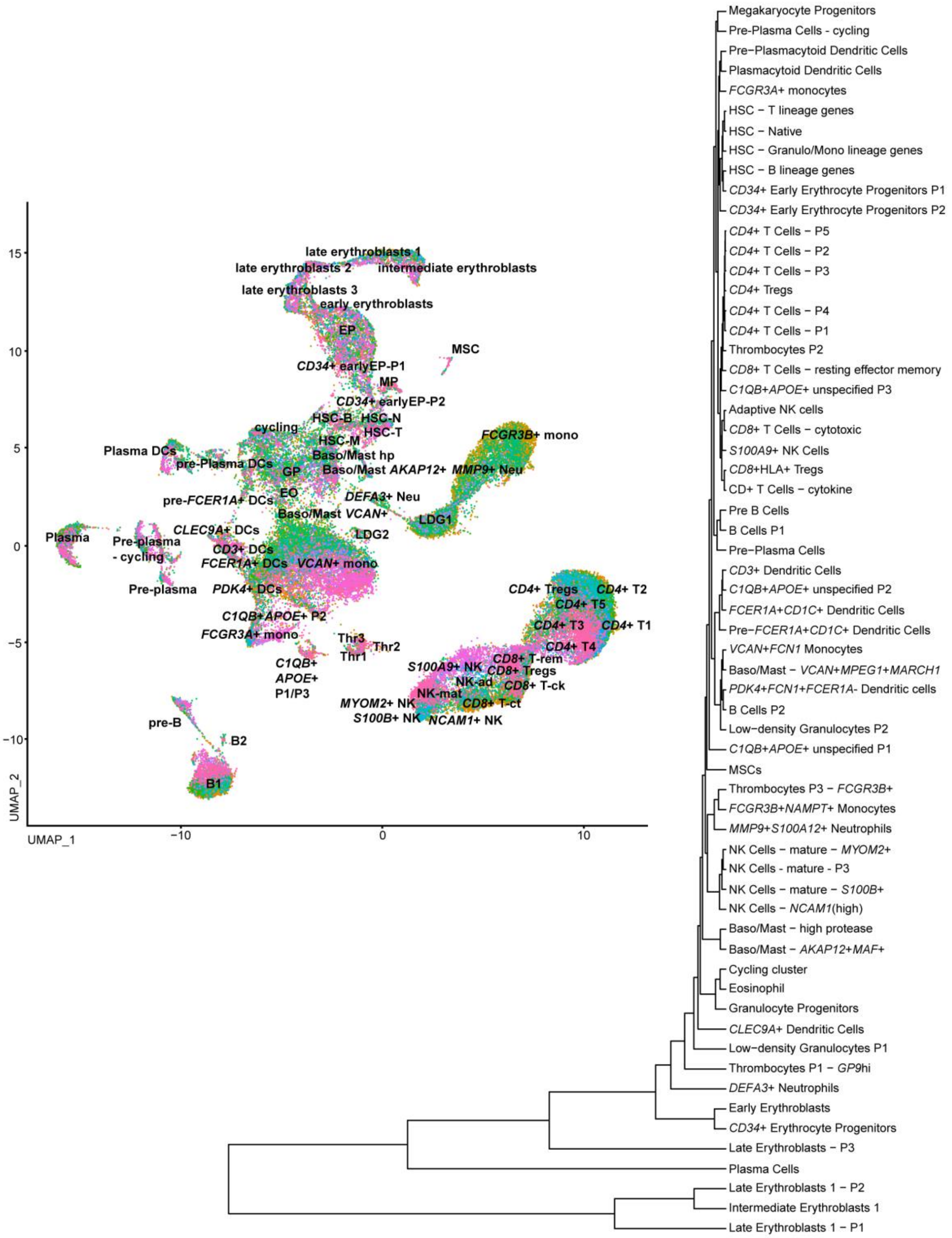

Figure S1

Unbiased BMAC clustering and hierarchical dendrogram. Colors represent different subjects. N = 13.

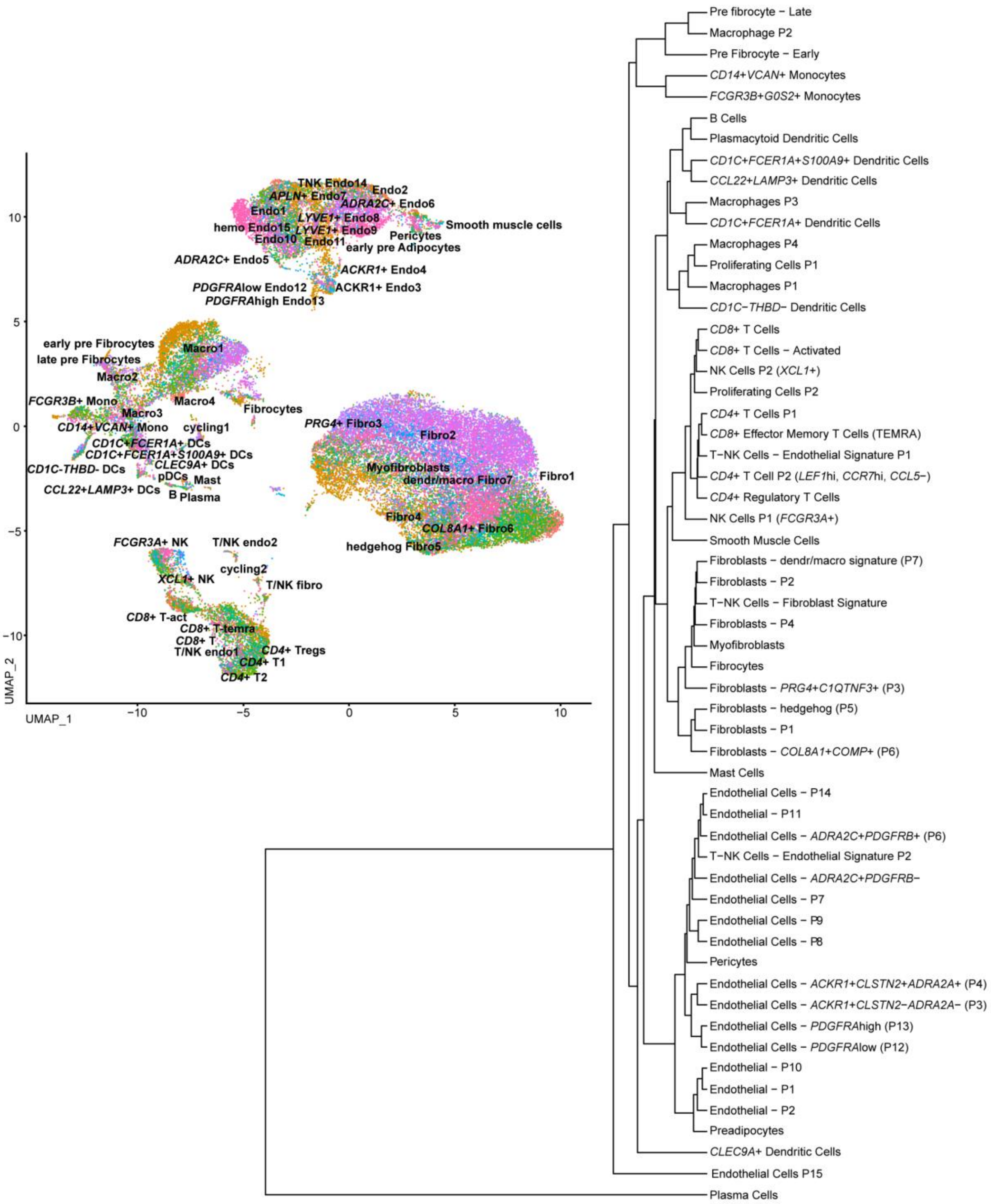
TNK Endo14
APLN+ Endo7
Endo2
ADRA2C+ Endo6
Endo1
LYVE1+ Endo8
Smooth muscle cells
hemo Endo15
LYVE1+ Endo9
Pericytes
Endo10
Endo11
early pre Adipocytes
ADRA2C+ Endo5
ACKR1+ Endo4
PDGFRAlow Endo12
ACKR1+ Endo3
PDGFRAhigh Endo13
early pre Fibrocytes
Macro1
late pre Fibrocytes
Macro2
FCGR3B+ Mono
Macro4
Fibrocytes
Macro3
CD14+VCAN+ Mono
cycling1
PRG4+ Fibro3
Fibro2
CD1C+FCER1A+ DCs
CD1C+FCER1A+S100A9+ DCs
CD1C-THBD- DCs
CLEC9A+ DCs
Myofibroblasts
pDCs
Mast
dendr/macro Fibro7
Fibro1
CCL22+LAMP3+ DCs
B
Plasma
Fibro4
COL8A1+ Fibro6
FCGR3A+ NK
T/NK endo2
hedgehog Fibro5
cycling2
XCL1+ NK
T/NK fibro
CD8+ T-act
CD8+ T-temra
CD8+ T
T/NK endo1
CD4+ Tregs
CD4+ T1
CD4+ T2
UMAP_2
UMAP_1
10
5
0
−5
−10
−10
−5
0
5
10
Pre fibrocyte − Late
Macrophage P2
Pre Fibrocyte − Early
CD14+VCAN+ Monocytes
FCGR3B+G0S2+ Monocytes
B Cells
Plasmacytoid Dendritic Cells
CD1C+FCER1A+S100A9+ Dendritic Cells
CCL22+LAMP3+ Dendritic Cells
Macrophages P3
CD1C+FCER1A+ Dendritic Cells
Macrophages P4
Proliferating Cells P1
Macrophages P1
CD1C−THBD− Dendritic Cells
CD8+ T Cells
CD8+ T Cells − Activated
NK Cells P2 (XCL1+)
Proliferating Cells P2
CD4+ T Cells P1
CD8+ Effector Memory T Cells (TEMRA)
T−NK Cells − Endothelial Signature P1
CD4+ T Cell P2 (LEF1hi, CCR7hi, CCL5−)
CD4+ Regulatory T Cells
NK Cells P1 (FCGR3A+)
Smooth Muscle Cells
Fibroblasts − dendr/macro signature (P7)
Fibroblasts − P2
T−NK Cells − Fibroblast Signature
Fibroblasts − P4
Myofibroblasts
Fibrocytes
Fibroblasts − PRG4+C1QTNF3+ (P3)
Fibroblasts − hedgehog (P5)
Fibroblasts − P1
Fibroblasts − COL8A1+COMP+ (P6)
Mast Cells
Endothelial Cells − P14
Endothelial − P11
Endothelial Cells − ADRA2C+PDGFRB+ (P6)
T−NK Cells − Endothelial Signature P2
Endothelial Cells − ADRA2C+PDGFRB−
Endothelial Cells − P7
Endothelial Cells − P9
Endothelial Cells − P8
Pericytes
Endothelial Cells − ACKR1+CLSTN2+ADRA2A+ (P4)
Endothelial Cells − ACKR1+CLSTN2−ADRA2A− (P3)
Endothelial Cells − PDGFRAhigh (P13)
Endothelial Cells − PDGFRAlow (P12)
Endothelial − P10
Endothelial − P1
Endothelial − P2
Preadipocytes
CLEC9A+ Dendritic Cells
Endothelial Cells P15
Plasma Cells

Figure S2

Unbiased ADSVF clustering and hierarchical dendrogram. Colors represent different subjects. N = 11.

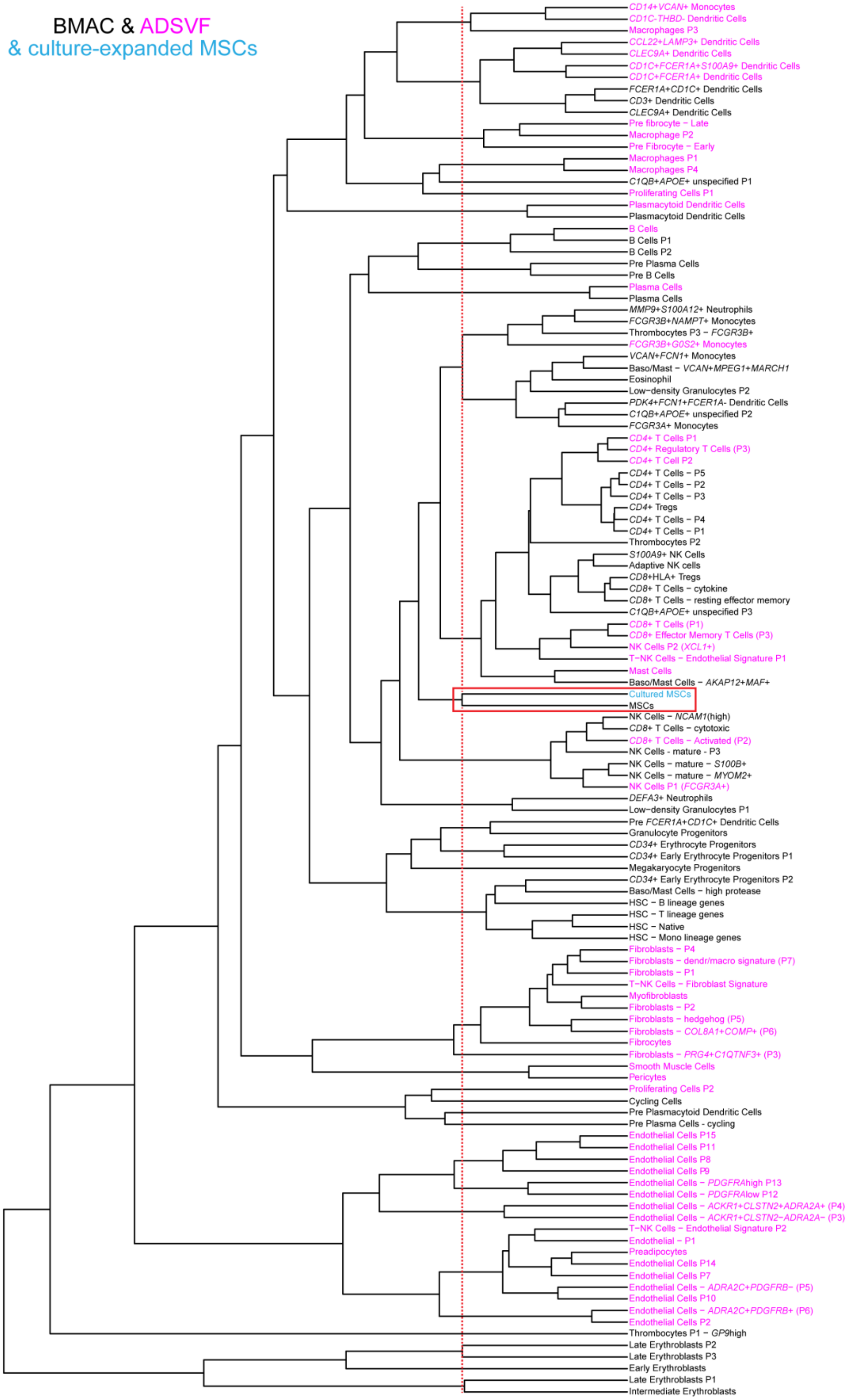

BMAC & ADSVF
& culture-expanded MSCs
CD14+VCAN+ Monocytes
CD1C-THBD- Dendritic Cells
Macrophages P3
CCL22+LAMP3+ Dendritic Cells
CLEC9A+ Dendritic Cells
CD1C+FCER1A+S100A9+ Dendritic Cells
CD1C+FCER1A+ Dendritic Cells
FCER1A+CD1C+ Dendritic Cells
CD3+ Dendritic Cells
CLEC9A+ Dendritic Cells
Pre fibrocyte − Late
Macrophage P2
Pre Fibrocyte − Early
Macrophages P1
Macrophages P4
C1QB+APOE+ unspecified P1
Proliferating Cells P1
Plasmacytoid Dendritic Cells
Plasmacytoid Dendritic Cells
B Cells
B Cells P1
B Cells P2
Pre Plasma Cells
Pre B Cells
Plasma Cells
Plasma Cells
MMP9+S100A12+ Neutrophils
FCGR3B+NAMPT+ Monocytes
Thrombocytes P3 − FCGR3B+
FCGR3B+G0S2+ Monocytes
VCAN+FCN1+ Monocytes
Baso/Mast − VCAN+MPEG1+MARCH1
Eosinophil
Low−density Granulocytes P2
PDK4+FCN1+FCER1A- Dendritic Cells
C1QB+APOE+ unspecified P2
FCGR3A+ Monocytes
CD4+ T Cells P1
CD4+ Regulatory T Cells (P3)
CD4+ T Cell P2
CD4+ T Cells − P5
CD4+ T Cells − P2
CD4+ T Cells − P3
CD4+ Tregs
CD4+ T Cells − P4
CD4+ T Cells − P1
Thrombocytes P2
S100A9+ NK Cells
Adaptive NK cells
CD8+HLA+ Tregs
CD8+ T Cells − cytokine
CD8+ T Cells − resting effector memory
C1QB+APOE+ unspecified P3
CD8+ T Cells (P1)
CD8+ Effector Memory T Cells (P3)
NK Cells P2 (XCL1+)
T−NK Cells − Endothelial Signature P1
Mast Cells
Baso/Mast Cells − AKAP12+MAF+
Cultured MSCs
MSCs
NK Cells − NCAM1(high)
CD8+ T Cells − cytotoxic
CD8+ T Cells − Activated (P2)
NK Cells − mature - P3
NK Cells − mature − S100B+
NK Cells − mature − MYOM2+
NK Cells P1 (FCGR3A+)
DEFA3+ Neutrophils
Low−density Granulocytes P1
Pre FCER1A+CD1C+ Dendritic Cells
Granulocyte Progenitors
CD34+ Erythrocyte Progenitors
CD34+ Early Erythrocyte Progenitors P1
Megakaryocyte Progenitors
CD34+ Early Erythrocyte Progenitors P2
Baso/Mast Cells − high protease
HSC − B lineage genes
HSC − T lineage genes
HSC − Native
HSC − Mono lineage genes
Fibroblasts − P4
Fibroblasts − dendr/macro signature (P7)
Fibroblasts − P1
T−NK Cells − Fibroblast Signature
Myofibroblasts
Fibroblasts − P2
Fibroblasts − hedgehog (P5)
Fibroblasts − COL8A1+COMP+ (P6)
Fibrocytes
Fibroblasts − PRG4+C1QTNF3+ (P3)
Smooth Muscle Cells
Pericytes
Proliferating Cells P2
Cycling Cells
Pre Plasmacytoid Dendritic Cells
Pre Plasma Cells - cycling
Endothelial Cells P15
Endothelial Cells P11
Endothelial Cells P8
Endothelial Cells P9
Endothelial Cells − PDGFRAhigh P13
Endothelial Cells − PDGFRAlow P12
Endothelial Cells − ACKR1+CLSTN2+ADRA2A+ (P4)
Endothelial Cells − ACKR1+CLSTN2−ADRA2A− (P3)
T−NK Cells − Endothelial Signature P2
Endothelial − P1
Preadipocytes
Endothelial Cells P14
Endothelial Cells P7
Endothelial Cells − ADRA2C+PDGFRB− (P5)
Endothelial Cells P10
Endothelial Cells − ADRA2C+PDGFRB+ (P6)
Endothelial Cells P2
Thrombocytes P1 − GP9high
Late Erythroblasts P2
Late Erythroblasts P3
Early Erythroblasts
Late Erythroblasts P1
Intermediate Erythroblasts

Figure S3

Unbiased hierarchical dendrogram of BMAC (black), ADSVF (pink) and cultured MSCs (blue) populations. Cell and cell group relationships to the right of the red dotted line are transcriptionally more related than cultured to non-cultured MSCs and relationships to the left are transcriptionally less related. Most cells with known functional differences are transcriptionally more related than fresh and cultured MSCs. N = 13 BMAC, 11 ADSVF, 1 cultured MSCs samples.

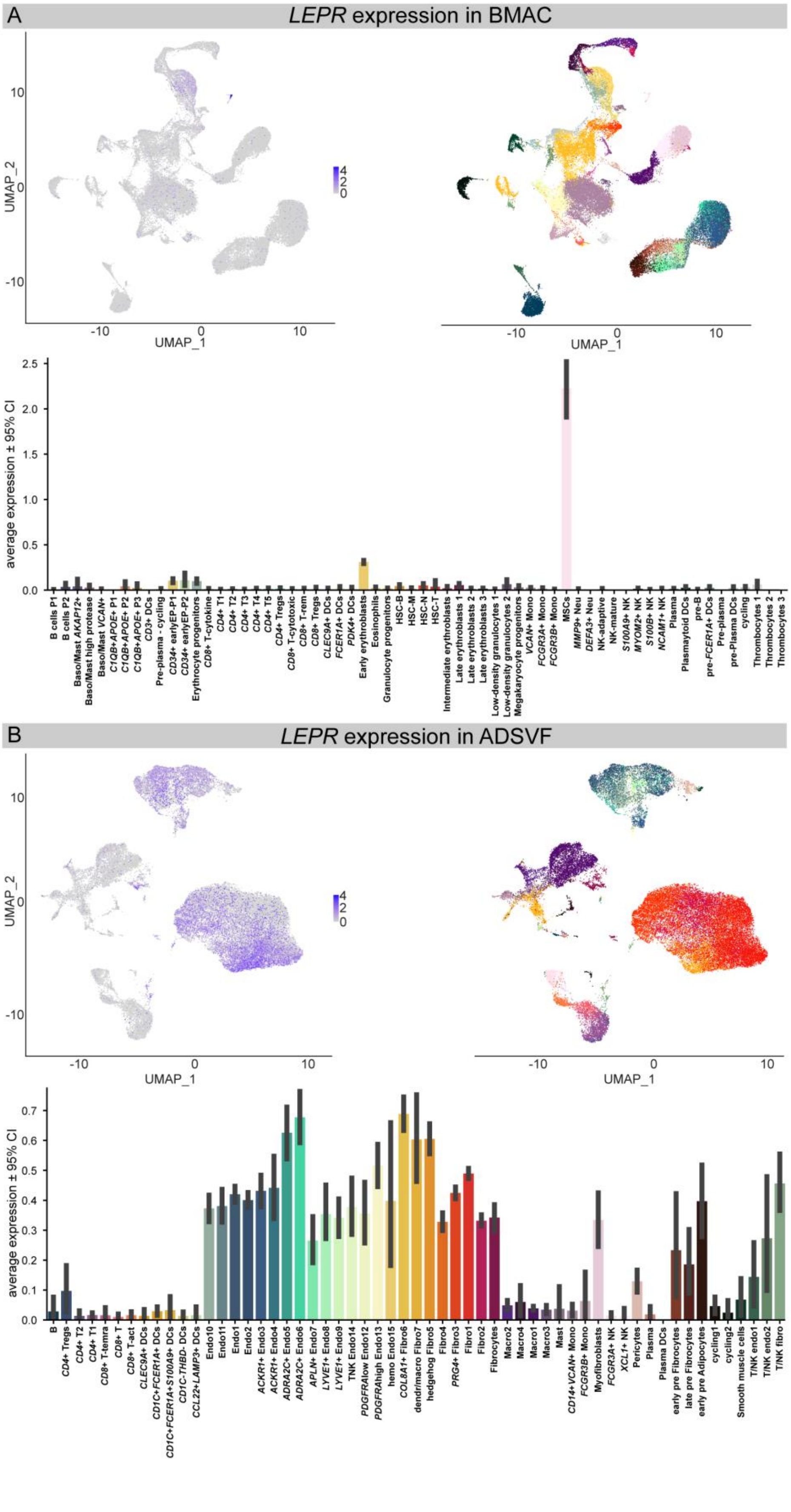

A
LEPR expression in BMAC
UMAP_1
UMAP_2
average expression ± 95% CI
B cells P1
B cells P2
Baso/Mast AKAP12+
Baso/Mast high protease
Baso/Mast VCAN+
C1QB+APOE+ P1
C1QB+APOE+ P2
C1QB+APOE+ P3
CD3+ DCs
Pre-plasma - cycling
CD34+ earlyEP-P1
CD34+ earlyEP-P2
Erythrocyte progenitors
CD8+ T-cytokine
CD4+ T1
CD4+ T2
CD4+ T3
CD4+ T4
CD4+ T5
CD4+ Tregs
CD8+ T-cytotoxic
CD8+ T-rem
CD8+ Tregs
CLEC9A+ DCs
FCER1A+ DCs
PDK4+ DCs
Early erythroblasts
Eosinophils
Granulocyte progenitors
HSC-B
HSC-M
HSC-N
HSC-T
Intermediate erythroblasts
Late erythroblasts 1
Late erythroblasts 2
Late erythroblasts 3
Low-density granulocytes 1
Low-density granulocytes 2
Megakaryocyte progenitors
VCAN+ Mono
FCGR3A+ Mono
FCGR3B+ Mono
MSCs
MMP9+ Neu
DEFA3+ Neu
NK-adaptive
NK-mature
S100A8+ NK
MYOM2+ NK
S100B+ NK
NCAM1+ NK
Plasma
Plasmayoid DCs
pre-B
pre-FCER1A+ DCs
Pre-plasma
pre-Plasma DCs
cycling
Thrombocytes 1
Thrombocytes 2
Thrombocytes 3
B
LEPR expression in ADSVF
UMAP_1
UMAP_2
average expression ± 95% CI
B
CD4+ Tregs
CD4+ T2
CD4+ T1
CD8+ T-temra
CD8+ T
CD8+ T-act
CLEC9A+ DCs
CD1C+FCER1A+ DCs
CD1C+FCER1A+S100A9+ DCs
CD1C-THBD- DCs
CCL22+LAMP3+ DCs
Endo10
Endo11
Endo1
Endo2
ACKR1+ Endo3
ACKR1+ Endo4
ADRA2C+ Endo5
ADRA2C+ Endo6
APLN+ Endo7
LYVE1+ Endo8
LYVE1+ Endo9
TNK Endo14
PDGFRAlow Endo12
PDGFRAhigh Endo13
hemo Endo15
COL8A1+ Fibro6
dendr/macro Fibro7
hedgehog Fibro5
Fibro4
PRG4+ Fibro3
Fibro1
Fibro2
Fibrocytes
Macro2
Macro4
Macro1
Macro3
Mast
CD14+VCAN+ Mono
FCGR3B+ Mono
Myofibroblasts
FCGR3A+ NK
XCL1+ NK
Pericytes
Plasma
Plasma DCs
early pre Fibrocytes
late pre Fibrocytes
early pre Adipocytes
cycling1
cycling2
Smooth muscle cells
T/NK endo1
T/NK endo2
T/NK fibro

Figure S4

Leptin receptor (*LEPR*) gene expression across **A)** bone marrow adipose concentrate (BMAC) and **B)** adipose-derived stromal vascular fraction (ADSVF) cell (sub)populations. The left UMAP (uniform manifold approximation and projection) plots show *LEPR* expression levels per cell. The colors of the right UMAP plots correspond to the cell (sub)populations in the bar plots. N = 13 BMAC, 11 ADSVF.

CI, confidence interval

B, B cell

Baso/Mast, basophil/mast cells

DCs, dendritic cells

Endo, endothelial cells,

EP, erythroblast progenitor

Fibro, fibroblasts

HSC-N/B/M/T, hematopoietic stem cells-native/B-/monocyte/T-cell lineage

Macro, macrophages

Mono, monocytes

MSCs, mesenchymal stromal cells

Neu, neutrophils

NK, natural killer cells

P1-3, population 1-3

Plasma, plasma cells

T, T cell,

Temra, terminally differentiated effector T cells

Tregs, regulatory T cells

Trem, resting effector memory T cells

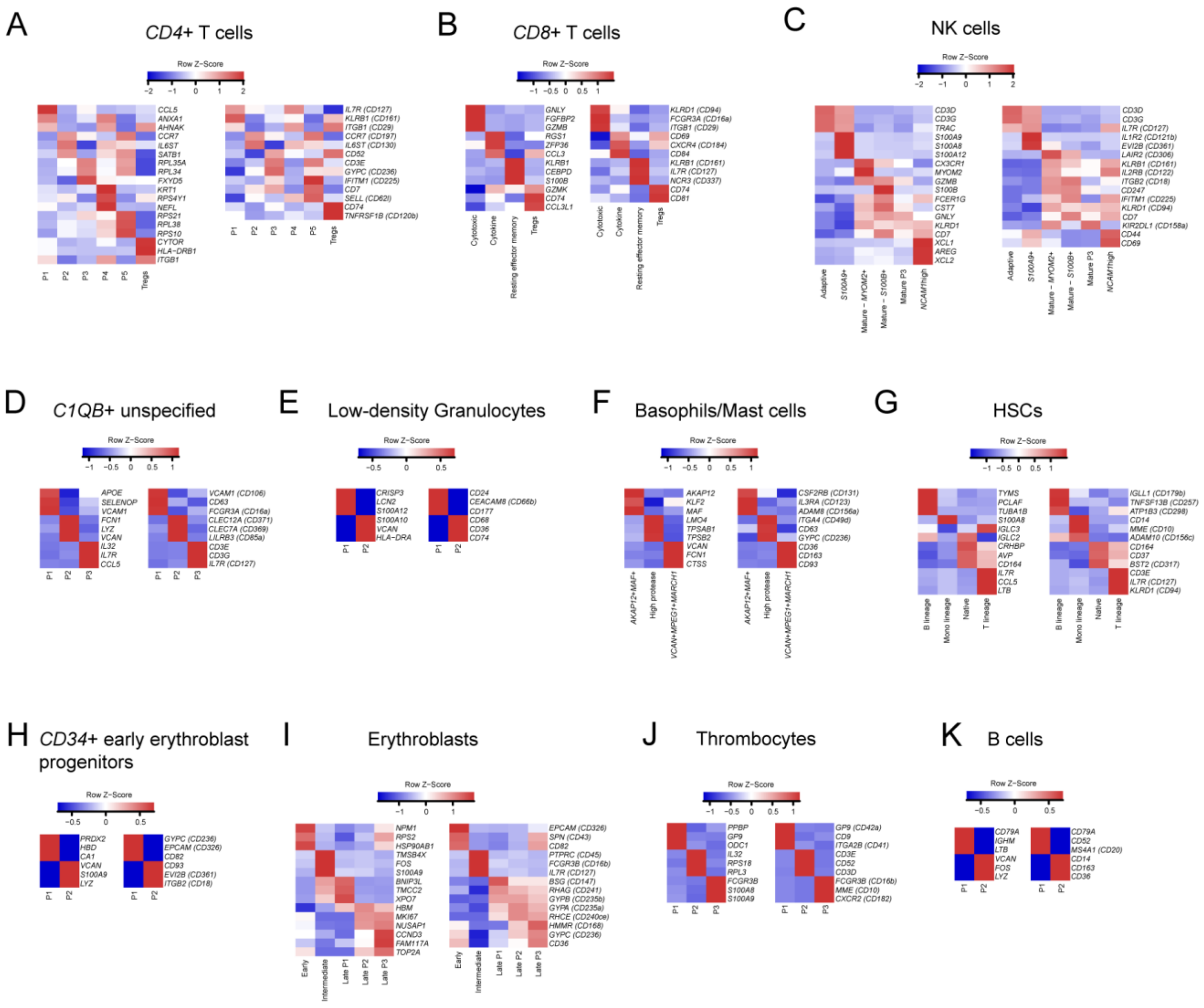

Figure S5

Unbiased detection of marker genes (left heatmap) and surface marker genes only (right heatmap) of BMAC cell subpopulations. N = 13.

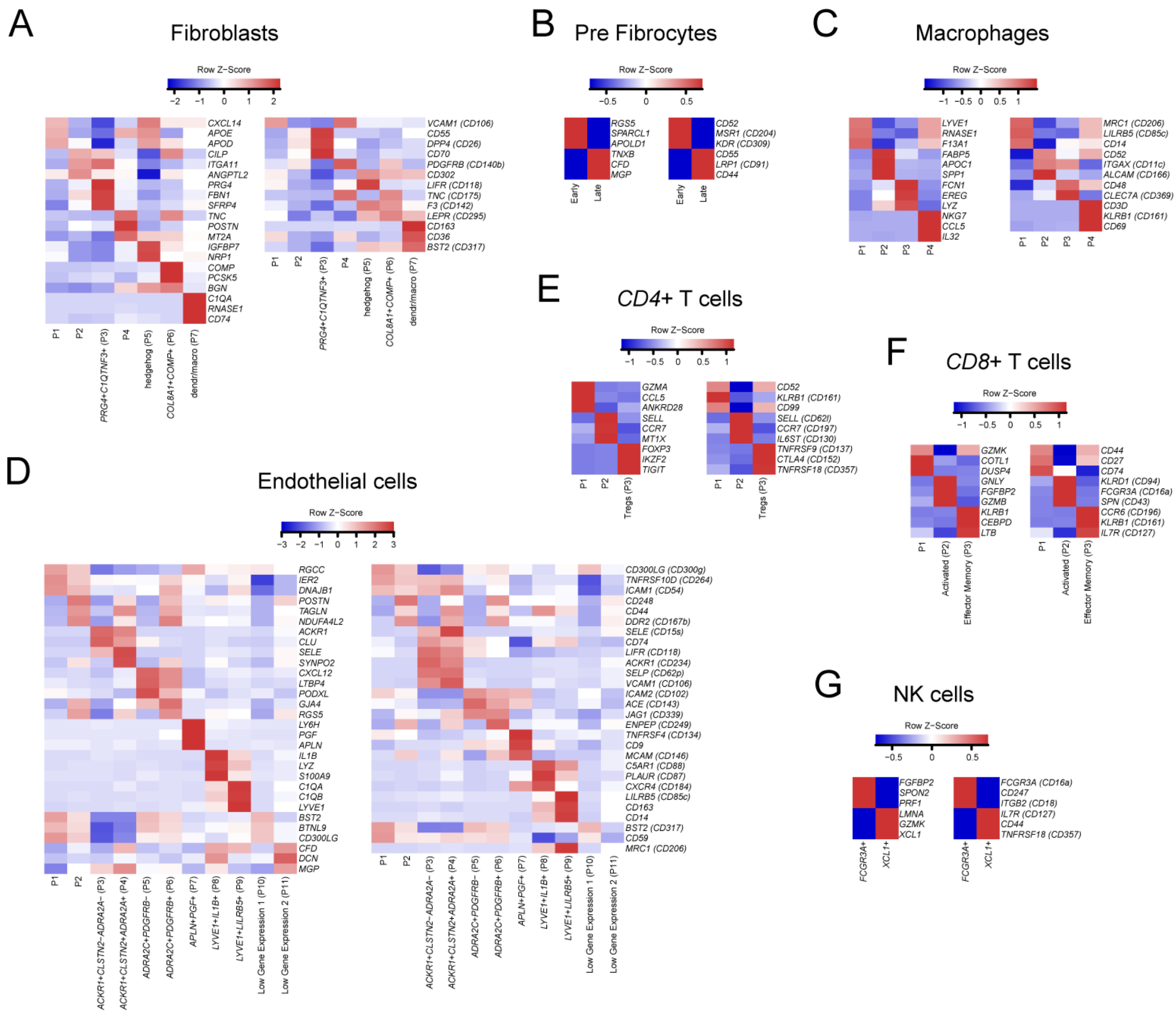

Figure S6

Unbiased detection of marker genes (left heatmap) and surface marker genes only (right heatmap) of ADSVF cell subpopulations. N = 11

Supplemental notes 1:

BMAC cell (sub)population frequencies and landmark gene expression

MSCs accounted for 0.22 ± 0.22% (mean ± SD) of RBC-depleted BMAC cells (Table S2). The top five MSC transcriptional markers were *CXCL12*, *IGFBP5*, *APOE*, *FABP4*, and *LEPR*, both if sorted for average logarithmic fold change (avg_logFC) or adjusted p-value (adj_p) (Fig. 3, Table S6).

The *CD4*+ T cell group (18.69 ± 10.60% of BMAC cells) contained 6 transcriptionally distinct *CD4*-expressing T cell populations. The top five positive DEGs of this group were *IL7R*, *LTB*, *TRAC*, *TXF7*, and *TRBC2* (Fig. 3B, Table S12). *CD4*+ regulatory T cells (*CD4*+ Tregs) accounted for 1.90 ± 1.98% of cells (Table S2) and were identified by their DE genes *TIGIT*, *IL2RA*, *CTLA4*, and *FOXP3* (*77*), which were in the top 17 DE genes if compared with all BMAC cells (Table S12). In our dataset, the top DE genes to distinguish this subpopulation from other *CD4*+ T cells were *CYTOR*, *HLA-DRB1*, *ITGB1*, *CD74*, and *NEAT1* (Fig. S5A, Table S13). Surface markers to efficiently separate BMAC-derived *CD4*+ Tregs from other *CD4*+ cells could include *ITGB1* (CD29), *CD74*, *TNFRSF1B* (CD120b), *IL2RA* (CD25), and *CTLA4* (CD152) (Fig. S5A, Table S13). The other five *CD4*+ T cell populations (P1-P5) were characterized by the following transcriptional features: *CD4*+ T cells P1, accounting for 6.04 ± 5.21% cells, differentially expressed *CCL5*, *ANXA1*, *AHNAK*, *KLRB1* (CD161), and *GNLY*, and additional DE surface marker genes were *IL7R* (CD127), *ITGB1* (CD29), *CD69*, and *KLRD1* (CD94) (Fig. S5A, Tables S13). The unbiased DE genes list of the *CD4*+ T cells P1 compared with all BMAC cells can be found in Table S13. *CD4*+ T cells P2 accounted for 4.47 ± 4.94% of BMAC cells and were characterized by their differential expression of *CCR7* (CD197), *IL6ST* (CD130), *SATB1*, *IGKC*, and *TSHZ2* (Fig. S5A, Table S13). The unbiased DE genes list of the *CD4*+ T cells P2 compared

with all BMAC cells can be found in Table S7. The *CD4*+ T cells P3 accounted for 2.86 ± 6.81% of BMAC cells and were characterized by their differential expression of *RPL35A*, *RPL34*, *FXYD5, GAPDH*, and *CD7*, and by the DE surface marker genes *CD52, CD37*, *CD3E*, and *GYPC* (CD236) (Fig. S5A, Table S13). The unbiased DE genes list of the *CD4*+ T cells P3 compared with all BMAC cells can be found in Table S7. The *CD4*+ T cells P4 accounted for 2.65 ± 6.26% of BMAC cells and were characterized by their distinct expression of *KRT1* and *NEFL*. Further DE genes included *RPS4Y1*, *BIRC3*, and *RPS17*, and the DE surface marker genes *ITGB1* (CD29), and *CD84* (Figure 2A, Table S8). The unbiased DE genes list of the *CD4*+ T cells P4 compared with all BMAC cells can be found in Table S13. Lastly, the smallest *CD4*+ T cell population, P5, encompassing 0.78 ± 2.75%, distinctly expressed the surface markers *CSF3R*, *IFITM1* (CD225), *CD7*, *SELL* (CD62l), and *CCR7* (CD197). The top DE genes included ribosomal proteins (Fig. S5A, Table S13). The unbiased DE genes list of the *CD4*+ T cells P5 compared with all BMAC cells can be found in Table S13.

The *CD8*+ T cells group (5.37 ± 3.44% of BMAC cells) contained four transcriptionally distinct *CD8A*-expressing T cell subpopulations. The top 5 positive DE genes of this group were *CCL5*, *GZMK*, *CCL4*, *IL32*, and *DUSP2* (Fig. 3, Table S12). Differentially expressed surface marker genes included *CD8A*, *KLRD1* (CD94), *CD3D*, *KLRB1* (CD161), and *CD2* (Fig. 3B, Table S7). The largest subpopulation accounted for 2.32 ± 2.93% of BMAC cells and we termed them “cytotoxic” due to their pronounced expression of *CCL5*, *GNLY, NKG7*, and *KLRG1* (Table S14), as suggested by previous detailed single-cell analysis of T cells (*77*). Their top DE genes compared with the other *CD8*+ T cells included *GNLY, FGFBP2, GZMB, GZMH*, and *CX3CR1* (Fig. S5B, Table S14) and the surface markers *KLRD1* (CD94), *FCGR3A* (CD16a), *ITGB1* (CD29), *LILRB1* (CD85j), and *SPN* (CD43) (Fig. S5B, Table S14). The unbiased DE genes list of these cytotoxic

CD8+ T cells compared with all BMAC cells can be found in Table S9. A second *CD8*+ T cell subpopulation strongly expressed *CCL4*, *CCL3*, and *XCL2* (Table S14) and was therefore termed "cytokine" as suggested previously (*77*). This subpopulation accounted for 1.59 ± 1.32% of BMAC cells and highly expressed *RGS1*, *ZFP36*, *CCL3*, *JUNB*, and *CCL4*, and the surface markers *CD69*, *CXCR4* (CD184), *CD84*, *CRTAM* (CD355), and *MS4A1* (CD20), if compared with other *CD8*+ T cells (Fig. S5B, Table S14). The unbiased list of DE genes of this *CD8*+ cytokine T cell subpopulation compared with all BMAC cells can be found in Table S14. The third *CD8*+ T cell subpopulation accounted for 0.95 ± 1.35% of BMAC cells and based on their expression profile including *KLRB1* and *JAML*/*AMICA1* (Fig. S5B, Table S4+S14), they were identified as *CD8*+ resting effector memory T cells (*77*). The top DE genes of this subpopulation compared with all *CD8*+ T cells were *KLRB1* (CD161), *CEBPD*, *S100B*, *IL7R* (CD127), and *LTB*. Additional DE surface marker genes included *NCR3* (CD337), *CCR6* (CD196), and *DPP4* (CD26) (Fig. S5B, Table S14). The unbiased list of DE genes of the *CD8*+ resting effector memory T cells compared with all BMAC cells can be found in Table S14. Lastly, the smallest subpopulation encompassed 0.52 ± 1.41% of all BMAC cells (Table S2) and was termed *CD8*+*HLA*+ Tregs based on their expression of *HLA*-genes and *HLA*-associated *CD74* (*106*, *107*). This subpopulation differentially expressed *GZMK*, *CD74*, *CCL3L1*, *FXYD5*, and *HLA-DRB5* (Fig. S5B, Table S14). Additionally, they could transcriptionally be distinguished from other *CD8*+ T cells by their differential surface marker gene expression of *CD74*, *CD69*, *CD81*, *CD27*, and *CXCR3* (CD183) (Fig. S5B, Table S14). The unbiased list of DE genes of these *CD8*+*HLA*+ Tregs compared with all BMAC cells can be found in Table S14.

The natural killer (NK) cells group (8.83 ± 6.49% of BMAC cells) contained six transcriptionally distinct subpopulations. The top five positive DE genes of this group were *GNLY*, *NKG7*, *PRF1*,

KLRD1, and *FGFBP2* (Figure 3A, Table S12), whereas the top surface marker genes were *KLRD1* (CD94), *CD247*, *KLRB1* (CD161), *CD7*, and *IL2RB* (CD122) (Figure 3B, Table S7). Three of the six subpopulations were classified as “mature” NK cells due to their pronounced expression of *PRF1*, *FCGR3A*, *SPON2*, and *GZMB* (Table S15) (*78*). The largest of these mature populations accounted for 2.84 ± 3.78% of BMAC cells (Table S2) and was further termed “*MYOM2+*” for its DE genes including *MYOM2*, *CX3CR1*, *GZMB*, *HIPK2*, and *LAIR2* (Fig. S5C, Table S15). DE surface marker genes of this population included *CD38, FCGR3A* (CD16a), *LAIR2* (CD306), *KLRB1*, (CD161), and *IL2RB* (CD18) (Fig. S5C, Table S15) compared with other NK subpopulations. The unbiased list of DE genes of these mature *MYOM2*+ NK cells compared with all BMAC cells can be found in Table S15. A second mature NK subpopulation accounted for 1.04 ± 2.93% of BMAC cells (Table S2) and was termed “*S100B*+” according to its distinct expression of *S100B*, *FCER1G*, *CST7*, *CTSW*, and *SPON2* compared with other NK subpopulations (Fig. S5C, Table S15). DE surface marker genes included *CD7, FCGR3A* (CD16a), *ITGB2* (CD18), *CD247,* and *IFITM1* (CD225), which overlapped with the other *MYOM2*+ mature subpopulation (Fig. S5C, Table S15). The unbiased list of DE genes of these mature *S100B*+ NK cells compared with all BMAC cells can be found in Table S15. The third mature NK subpopulation (mature P3) accounted for 1.37 ± 3.08% of BMAC cells and was found at the intersection between mature and adaptive subpopulations (Fig. S1). The DE genes of this population were *GNLY*, *KLRD1*, *CD7*, *HBB*, and *KIR2DL1* and DE surface marker genes included *KLRD1* (CD94), *CD7*, and *KIR2DL1* (158a) (Fig. S5C, Table S15). The unbiased list of DE genes of this third mature NK subpopulation compared with all BMAC cells can be found in Table S15. The second largest NK subpopulation was termed “adaptive” because of their high expression of *CD3D/E/G* and *IL32* (*78*) (Fig. S5C, Tables S15) and it accounted for 2.73 ± 4.55% of all BMAC cells (Table S2). Top DE genes and

surface markers compared with other NK cells were *CD3D*, *CD3G*, *TRAC*, *IL7R* (CD127), *GZMK*, *CD84*, and *CD8A* (Fig. S5C, Table S15). The unbiased list of DE genes of this adaptive NK subpopulation compared with all BMAC cells can be found in Table S15. The fifth NK subpopulation was defined by their high expression of *NCAM1* (CD56) (*78*) and encompassed 0.42 ± 0.28% of BMAC cells (Table S2). Their top DE genes compared with other NK cells included *XCL1*, *AREG*, *XCL2*, *SELL*, and *CCL3* (Fig. S5C, Table S15) and the top DE surface marker genes were *SELL* (CD62l), *KLRC1* (CD159a), *CD44*, *CD69*, and *IL2RB* (CD122) (Fig. S5C, Table S15). The unbiased list of DE genes of this *NCAM1*^{high} NK subpopulation compared with all BMAC cells can be found in Table S15. Lastly, the sixth NK subpopulation accounted for 0.42 ± 0.40% of BMAC cells (Table S2) and the top expressed DE genes were *S100A9*, *S100A8*, *S100A12*, *DUSP1*, and *LYZ*, and the top DE surface marker genes were *FCGR3B* (CD16b), *CSF3R* (CD114), *IL1R2* (CD121b), *IL7R* (CD127), and *EVI2B* (CD361), compared with other NK subpopulations (Fig. S5C, Table S15). The unbiased list of DE genes of this *S100A9*+ NK subpopulation compared with all BMAC cells can be found in Table S15.

The transcriptional profile of three monocyte populations (19.06 ± 7.93% of BMAC cells) allowed their direct identification in BMAC, which we related with the detailed monocyte analysis by (*79*). *VCAN*+*FCN1*+ monocytes accounted for 14.06 ± 7.93% of BMAC cells (Table S2) and the top DE genes were *VCAN*, *FCN1*, *THBS1*, *CTSS*, and *SLC11A1* (Fig. 3A, Tables S12+S16). The top DE surface marker genes of this population were *CD14*, *CD36*, *CD163*, *CD93*, and *CD68* (Fig. 3B, Table S7). *FCGR3A*+ monocytes accounted for 2.00 ± 1.68% of BMAC cells (Table S2), the top DE genes were *FCGR3A*, *MS4A7*, *CDKN1C*, *AIF1*, and *LST1* (Fig. 3A, Tables S12+S16). The top DE surface marker genes were *FCGR3A* (CD16a), *LILRB2* (CD85d), *TNFRSF1B* (CD120b), *CSF1R* (CD115), and *PECAM1* (CD31) (Fig. 3B, Table S7). The third, *FCGR3B*+*NAMPT*+

monocyte population represented 3.00 ± 5.93% of cells (Table S2) and the top DE genes were *FCGR3B*, *NAMPT*, *IFITM2*, *CXCL8*, and *CXCR2* (Fig. 3A, Tables S12+S16). The top DE surface marker genes were *FCGR3B* (CD16b), *CXCR2* (CD182), *MME* (CD10), *CSF3R* (CD114), and *IL1R2* (CD121b) (Figure 3B, Table S7). These populations are in accordance with Villani and colleagues' Mono1, Mono2, and Mono3 monocyte populations (*79*).

A population encompassing 0.57 ± 0.64% of BMAC cells prominently expressed *C1QB*, *APOE*, *C1QA*, *SELENOP*, and *VCAM1* (Fig. 3A, Table S12) and we were unable to directly annotate these cells even though their transcriptional profile appeared to be unique (Table S17). The distinct expression of *C1QA/B* pointed us to the direction of bone marrow monocytes/macrophages and dendritic cells (*139*), but the expression of *APOE*, *SELENOP*, and *VCAM1* was specific to MSCs as opposed to the annotated, aforementioned cell types (Table S4). We then subclustered this population separately and found a total of three transcriptionally distinct subpopulations (P1-P3). The *C1QB+APOE+* P1 accounted for 0.30 ± 0.37% of BMAC cells (Table S2) and the DE genes compared with the other two subpopulations were *APOE*, *SELENOP*, *VCAM1*, *LGMN*, and *HMOX1*, and the top five DE surface marker genes were *VCAM1* (CD106), *CD63*, *FCGR3A* (CD16a), *CD59*, and *ITGAD* (CD11d) (Fig. S5D, Table S17). The second *C1QB+APOE+* subpopulation accounted for 0.20 ± 0.22% of BMAC cells (Table S2) and DE genes included *FCN1*, *LYZ*, *VCAN*, *S100A9*, and *S100A8*, and the surface marker genes *CLEC12A* (CD371), *CLEC7A* (CD369), *LILRB3* (CD85a), *CXCR4* (CD184), and *CD44*, compared with the other two subpopulations (Fig. S5D, Table S17). Lastly, the third *C1QB+APOE+* subpopulation encompassed only 0.07 ± 0.13% of BMAC cells (Table S2) and the DE genes included *IL32*, *IL7R*, *CCL5*, *NKG7*, *GNLY* and the DE surface marker genes *CD3E*, *CD3G*, *IL7R* (CD127), *CD7*, and *KLRB1* (CD161), compared with the *C1QB+APOE+* P1/2 (Fig. S5D, Table S17). The unbiased

list of DE genes of these three populations compared with all BMAC populations can be found in Table S17.

We identified 7 dendritic cell (sub)populations (6.58 ± 2.90% of BMAC cells) and if applicable, we related them to the detailed dendritic cell analysis by Villani and colleagues (*79*). 0.23 ± 0.19% were *CLEC9A*+ dendritic cells (Table S2), termed DC1 in (*79*); their top five positive DE genes were *HLA-DQA1*, *HLA-DPB1*, *HLA-DPA1*, *HLA-DRB1*, and *HLA-DRA* (avg_logFC-sorted) and *DNASE1L3*, *CLEC9A*, *CLNK*, *IDO1*, and *BATF3* (adj_p-sorted) (Fig. 3A, Tables S12 + S18). The top DE surface marker genes compared with all BMAC cells included *CLEC9A* (CD370), *SLAMF8* (CD353), *SLAMF7* (319), *FLT3* (CD135), and *CD83* (Fig. 3B, Table S7). 1.19 ± 0.69% were *FCER1A*+*CD1C*+ dendritic cells, termed DC2 in (*79*), and their top five DE genes were *HLA-DPB1*, *HLA-DPA1*, *HLA-DRA*, *HLA-DQA1*, and *HLA-DRB1* (Fig. 3A, Tables S12 + S18). DE surface marker genes included *CD74*, *CLEC10A* (CD301), *CD1C*, *CD1E*, and *IL13RA1* (CD213a1) compared with all BMAC cells (Fig. 3B, Table S7). A second *FCER1A*+ population was detected, which accounted for 0.51 ± 0.44% of BMAC cells and the top DE genes included *FCER1A*, *HLA-DPA1*, *HLA-DRB1*, *HLA-DRA*, and *CD74* (avg_logFC-sorted) and additionally *CLSPN*, *FAM111B*, *CLEC10A*, and *ENHO* (adj_p-sorted), compared with all BMAC cells (Fig. 3A, Tables S12 + S18). DE surface marker genes included *CLEC10A* (CD301), *FLT3* (CD135), *CD1C, HAVCR2* (CD366), and *ALCAM* (CD166) (Fig. 3B, Tables S7 + S18). Given their UMAP visualization, they could be pre-*FCER1A*+*CD1C*+ dendritic cells (Fig. S1). This is further supported by *CTSV* and *SPATS2L* expression (Table S18), which marked pre-dendritic cells previously (*76*). Another population was very similar to *FCER1A*+*CD1C*+ dendritic cells, but it additionally expressed *CD3* and other genes typically associated with T cells. This population was therefore termed *CD3*+ dendritic cells. It accounted for only 0.05 ± 0.06 % BMAC cells and the

top DE genes included *HLA-DQA1*, *HLA-DPB1*, *HLA-DPA1*, *HLA-DRA*, and *CD74* (avg_logFC-sorted) and *CLEC10A*, *ENHO*, *CCL19*, *FCER1A*, and *CD1C* (adj_p-sorted) (Fig. 3A, Table S12+S18). The list of DE surface marker genes included *CLEC10A* (CD301), *CD1C*, *FLT3* (CD135), *CD86*, and *CSF2RA* (CD116) (Fig. 3B, Table S7). Then, we found a *PDK4+FCN1+FCER1A*- dendritic cell cluster, termed DC4 in Villani et al.'s detailed analysis (*79*). This group was the largest DC population in BMAC (2.86 ± 1.86% of BMAC cells; Table S2). The top DE genes for this DC population were *PDK4*, *HLA-DRB1*, *MAFB*, *CST3*, and *FCN1* (Fig. 3A, Tables S12 + S18) and the top DE surface marker genes included *CD14*, *CD163*, *CD74*, *CD68*, and *CLEC7A* (CD369) (Fig. 3B, Table S7). 1.38 ± 0.96% of BMAC cells were plasmacytoid dendritic cells (Table S2), termed DC6 in (*79*), and their top DE genes were *TCF4*, *CLN8*, *CCDC50*, *GZMB*, and *IRF8* (Fig. 3A, Tables S12 + S18). The top DE surface marker genes were *LILRA4* (CD85g), *IL3RA* (CD123), *CD164*, *CLEC4C* (CD303), *CD74*, and *CXCR3* (CD183) (Fig. 3B, Table S7). We detected a second plasmacytoid DC cluster which accounted for 0.35 ± 0.30% of BMAC cells and distinctly expressed *IGLL1*, *TCF4*, *IRF8*, *STMN1*, and *SOX4* (avg_logFC-sorted) and *PLD4*, *RUNX2*, *SCT*, *JCHAIN*, and *SPIB* (adj_p-sorted) compared with all BMAC cells (Fig. 3A, Tables S12 + S18). DE surface marker genes included *IL3RA* (CD123), *LILRA4* (CD85g), *CLEC4C* (CD303), *TNFRSF21* (CD358), and *NGFR* (CD271) (Fig. 3B, Table S7). Given their UMAP proximity to the pre-classical DC cluster at the fork between classical and plasmacytoid DCs (Fig. S1), this cluster could be a pre-plasmacytoid DC population.

An *MPO+PRTN3+* population was previously defined as granulocyte progenitors (4.89 ± 3.31% of BMAC cells) (*76*). The top positive DEGs were *MPO*, *PRTN3*, *ELANE*, *AZU1*, and *CTSG* (Fig. 3A, Tables S7 + S19) and differentially expressed surface marker genes included *FUT4* (CD15),

SLC44A1 (CD92), *TNFSF13B* (CD257), *LAIR1* (CD305), and *ALCAM* (CD166) (Fig. 3B, Table S7).

Low density granulocytes accounted for 3.78 ± 5.15% of BMAC cells. Using *LTF*, *LCN2*, and *MMP8* as genetic markers (*110*) we found two transcriptionally distinct low density granulocyte subpopulations, P1 and P2, which accounted for 3.62 ± 5.14% and 0.16 ± 0.26% of all BMAC cells, respectively (Table S2). Their DEGs and surface markers compared with BMAC are found in (Fig. 3A+B, Tables S7 + S19) and the DEGs comparing P1 with P2 are found in (Fig. S5E, Table S19).

We identified two neutrophil populations (3.16 ± 6.79% of BMAC cells), a *MMP9+S100A12+* population and a *DEFA3+* population, which accounted for 2.90 ± 6.69% and 0.26 ± 0.23% of cells, respectively (Table S2). The top DEGs to identify the larger population were *MMP9*, *S100A12*, *S100A8*, *CYP4F3*, and *ALOX5AP* (Fig. 3A, Table S7 + S19) and the surface marker genes *FCGR3B* (CD16b), *IL1R2* (CD121b), *CSF3R* (CD114), *MME* (CD10), and *CD177* (Fig. 3B, Table S7). The smaller population is identified by distinct expression of *DEFA3*, *DEFA4*, *CEACAM8*, *BPI*, and *CD24* (avg_logFC-sorted) and additionally *CEACAM6* and *SLPI* (adj_p-sorted) (Fig. 3A, Table S7 + S19). DE surface marker for this *DEFA3+* neutrophils included *CEACAM8* (CD66b), *CEACAM6* (CD66c), *CD24*, *CD63*, and *CEACAM3* (CD66d) (Fig. 3B, Table S7). The transcriptional distinction between these two neutrophil population was based on the expression of *MMP9*-rich gelatinase neutrophil granules and *DEFA*-rich azurophil neutrophil granules (*140*).

Eosinophils (1.56 ± 1.04% of BMAC cells) were identified by their characteristic expression of eosinophil-derived neurotoxin (*RNASE2*) (*113*). The top DE genes were *LYZ*, *LGALS1*, *AC020656.1*, *RNASE2*, and *MPO* (avg_logFC-sorted) and *RETN* (adj_p-sorted) (Fig. 3A, Tables

S7 + S19). Top DE surface marker genes included *SLC44A1* (CD92), *CCR2* (CD192), *CD68*, *TNFSF13B* (CD257), and *CLEC12A* (CD371) (Fig. 3B, Table S7).

Basophils/Mast cells (0.56 ± 0.35% of BMAC cells) were identified by their distinct expression of *CLC* and *HDC* (*76*) and UMAP clustering suggested to perform separate subcluster analysis (Fig. 2A, Fig. S1) which resulted in the following three subpopulations: An *AKAP12+MAF+* subpopulation encompassed 0.10 ± 0.08% of BMAC cells and its DE genes were *AKAP12*, *KLF2*, *MAF*, *AREG*, and *MXD1*, and the surface marker genes *CSF2RB* (CD131), *IL3RA* (CD123), *ADAM8* (CD156a), *CR1* (CD35), and *CD9* (Fig. S5F, Table S20). The second population encompassed 0.26 ± 0.16% of cells (Table S2) and was termed "high protease" for their distinct expression of the tryptase genes *TPSAB1* and *TPSB2*. Other DE genes included *LMO4*, *HIST1H4C*, and *CTSG*, and the top surface marker genes were *ITGA4* (CD49d), *CD63*, *GYPC* (CD236), *KIT* (CD117), and *CD81* (Fig. S5F, Table S20). Lastly, a *VCAN+MPEG1+MARCH1+* subpopulation accounted for 0.20 ± 0.19 of BMAC cells and DE genes included *VCAN*, *FCN1*, *CTSS*, *LYZ*, and *S100A9*, and the top DE surface marker genes *CD36*, *CD163*, *CD93*, *CD74*, and *TNFRSF1B* (CD120b) compared with the other subpopulations (Fig. S5F, Table S20). The unbiased DE genes of these populations compared with all BMAC cells can be found in (Table S20).

Hematopoietic stem cells (HSCs) (1.89 ± 1.33% of BMAC cells) were identified by their distinct expression of *SPINK2* and *AVP* (*76*) and other DE genes included *CDK6*, *FAM30A*, and *MSI2*, and the surface marker genes *IGLL1* (CD179b), *CD34*, *PROM1* (CD133), *FLT3* (CD135), and *KIT* (CD117) compared with all BMAC cells (Figure 3A/B, Table S7). Detailed subcluster analysis revealed four HSC subpopulations: The largest subpopulation encompassed 0.66 ± 0.51% of BMAC cells (Table S2) and we termed it "native" for its downregulated lineage genes compared

with the other subpopulations, and for the upregulated *CD164*, which has been shown to be a marker for the earliest HSC (*108*). Other DE genes included *CRHBP*, *AC011139.1*, and *JUN*, and the surface marker genes *CD37*, *BST2* (CD317), and *CLEC9A* (CD370) (Fig. S5G, Table S21). "B cell lineage" HSCs accounted for 0.57 ± 0.52% of BMAC cells and DE genes included *TYMS*, *PCLAF*, *TUBA1B*, and *MPO*, and the surface marker genes *IGLL1* (CD179b), *TNFSF13B* (CD257), *ATP1B3* (CD298), *GYPC* (CD236), and *CD38* (Fig. S5G, Table S21). The "monocyte lineage" HSCs accounted for 0.58 ± 0.74% of BMAC cells and DE genes included *S100A8*, *IGLC3*, *IGLC2*, *S100A9*, and *IGKC*, and the surface marker genes *CD14*, *MME* (CD10), *ADAM10* (CD156c), *CD163*, and *CD36* compared with the other HSC subpopulations (Fig. S5G, Table S21). Lastly, the "T/NK cell lineage" HSC subpopulation accounted for only 0.08 ± 0.08% of BMAC cells and DE genes included *IL7R*, *CCL5*, *LTB*, *GNLY*, and *NKG7*, and the surface marker genes *CD3D*, *CD3E*, *IL7R* (CD127), *KLRD1* (CD94), and *CD7* (Fig. S5G, Table S21). The unbiased DE genes of these four HSC subpopulations compared with all BMAC cells can be found in (Table S21).

Erythroblast progenitors accounted for 3.09 ± 1.35% of BMAC cells. Two *CD34+* early erythroblast progenitor subpopulations clustered separate from the HSC cluster (Fig. S1). P1 accounted for 0.91 ± 0.64% of BMAC cells and DE genes included *PRDX2*, *HBD*, *CA1*, *HIST1H4C*, and *HBB*, and the surface marker genes *GYPC* (CD236), *EPCAM* (CD326), *CD82*, *IGLL1* (CD179b), and *TFRC* (CD71). P2 encompassed 0.10 ± 0.13% of cells and DE genes included *VCAN*, *S100A9*, *LYZ*, *CTSS*, and *S100A8*, and the DE surface marker genes *CD93*, *EVI2B*, *ITGB2*, *CXCR4*, and *CD74*, compared with P1 (Fig. S5H, Table S22). The unbiased list of DE genes can be found in Table S22. Both of these subpopulations have been described as lineage committed HSC/progenitor cell previously (*108*), along with subpopulations discussed in the HSC

paragraph above. A third, later erythroblast progenitor accounted for 2.08 ± 0.86% of BMAC cells and was characterized by their gradual decrease of *CD34* and increase of *HBB* and *HBA2* expressions (Fig. S1, Table S22).

The developmental stages of erythroblasts (9.70 ± 5.93% of BMAC cells) were split into five subpopulations which can transcriptionally be tracked (Fig. S1+S5I, Table S22).

Megakaryocyte progenitors encompassed 0.27 ± 0.33% of BMAC cells and the DE genes of this population included *PDLIM1*, *UBE2C*, *PF4*, *PPBP*, and *PLEK* (avg_logFC-sorted) and *CMTM5*, *ITGA2B* (CD41), *GP9* (CD42a), *TGFB1I1*, and *CALD1* (adj_p-sorted) (Fig. 4A, Tables S7 + S23). Additional DE surface marker genes included *GP1BA* (CD42b), and *SELP* (CD62p) (Fig. 3B, Table S7). This population has been described as a lineage committed HSC/progenitor cell previously (*108*).

DE genes of thrombocytes (1.05 ± 1.09% of BMAC cells) were *PPBP*, *PF4*, and others (Fig. 3A+B, Table S7). Three subpopulations were identified (P1-P3): P1 encompassed 0.65 ± 0.71% of cells and DE genes compared with the other subpopulations were *PPBP*, *GP9* (CD42a), *ODC1*, *PTCRA*, and *TSC22D1*, and additional DE surface marker genes were *CD9*, *ITGA2B* (CD41), *ITGB3* (CD61), and *ICAM2* (CD102) (Fig. S5J, Table S23). P2 accounted for 0.28 ± 0.33% of BMAC cells and DE genes included *IL32*, *RPS18*, *RPL3*, *RPS12*, and *RPS27*, and the surface marker genes *CD3E*, *CD52*, *CD3D*, *CD48*, and *IL7R* (CD127) (Fig. S5J, Table S23). Lastly, P3 accounted for 0.12 ± 0.25% of cells and DE genes included *FCGR3B* (CD16b), *S100A8*, *S100A9*, *NAMPT*, and *S100A12*, and the additional surface marker genes *MME* (CD10), *CXCR2* (CD182), *CSF3R* (CD114), and *EVI2B* (CD361) compared with the other two subpopulations (Fig. S5J, Table S23). The unbiased list of DE genes comparing these thrombocyte subpopulations with whole BMAC are found in Table S23.

The top DE genes of pre B Cells (1.05 ± 0.72% of BMAC cells) were *TCL1A*, *CD79B*, *IGHM*, *PCDH9*, and *VPREB3*, and additional surface marker genes included *CD79B*, *VPREB1* (CD179a), *CD79A*, *CD38*, and *CD24* (Fig. 3A+B, Table S7+S24).

B cells (4.57 ± 3.38% of BMAC cells) were characterized by their DE genes *CD79A*, *MS4A1* (CD20), *IGHM*, *BANK1*, and *IGHD*, and additional surface marker genes were *CD74*, *CD37*, and *CD79B* (Fig. 3A+B, Table S7). We found the following two subpopulations: P1 encompassed 4.48 ± 3.32% of BMAC cells and the top positive DE genes compared with P2 were *CD79A*, *IGHM*, *LTB*, *IGHD*, and *IGKC*, and additional DE surface marker genes included *CD52*, *MS4A1* (CD20), *CD79B*, and *FCER2* (CD23) (Fig. S5K, Table S24). P2 accounted for 0.10 ± 0.12% of BMAC cells and positive DE genes included *VCAN*, *FOS*, *LYZ*, *S100A8*, and *S100A9*, and the surface marker genes *CD14*, *CD163*, *CD36*, *C5AR1* (CD88), and *TNFSF13B* (CD257) (Fig. S5K, Table S24). The unbiased list of DE genes compared with all BMAC cells can be found in Table S24.

We found two pre plasma cell subpopulations (1.75 ± 1.41% of BMAC cells), the larger of which (1.32 ± 1.10% of cells) was driven by cycling/proliferation marker genes including *TOP2A*, *MKI67*, *CENPF* and others (Figure 3A, Table S24), and thus not further analyzed. The other pre plasma cells encompassed 0.43 ± 0.34% of cells and DE genes included *DNTT*, *VPREB1* (CD179a), *IGLL1* (CD179b), *VPREB3*, and *AKAP12*, and additional surface marker genes including *CD9*, *MME* (CD10), and *CD79B* (Fig. 3A+B, Table S7+S24).

Plasma cells accounted for 2.11 ± 2.02% of BMAC cells. The top DE genes of this *MZB1*+ population were *IGKC*, *IGLC2*, *IGHA1*, *IGLC3*, and *IGHG1* (Fig. 3A, Table S24). DE surface marker genes included *TNFRSF17* (CD269), *FCRL5* (CD307e), *SDC1* (CD138), *CD27*, and *CD38* (Fig. 3B, Table S7).

Lastly, a cluster of cycling cells (1.25 ± 0.94% of BMAC cells) was driven by high expression of *TOP2A*, *MKI67*, and many other proliferation/cell cycle markers (Fig. 3A, Table S25). Thus, this cluster was not further analyzed. However, its transcriptional signature has previously been suggested to have Pro B cell origin (*76*).

Supplemental notes 2:

ADSVF cell (sub)population frequencies and landmark gene expression

A subset of the cell population of perivascular origin, named pericytes (1.11 ± 0.75% of ADSVF), has been regarded a source of colony founding cells that could contribute to a culture expanded MSC population derived from adipose tissue (*53*). The top pericyte transcriptional markers included *POSTN*, *RGS5*, *NDUFA4L2*, *ACTA2*, and *TAGLN* (avg_logFC-sorted) and *STEAP4* and *CARMN* (adj_p-sorted) (Fig. 4A, Table S26). The previously reported pericyte markers *PDGFRB* (CD140b), *MCAM*, and *CSPG4* (NG2) were detected in 83.1%, 65.1%, and 28.6% of the pericyte cluster (Table S5). *MCAM* was also detected in all endothelial cell populations and preadipocytes and was highest expressed in smooth muscle cells (Fig. 4A, Table S5). *PDGFRB* was a very unspecific marker in ADSVF as it was detected in 45.4% of non-pericytes, including high expression in fibroblasts, myofibroblasts, fibrocytes, some endothelial cell populations, preadipocytes, and smooth muscle cells (Table S5+S26). Importantly, current pericyte identification/isolation strategies (*MCAM+PDGFRB+CD34-PTPRC-PECAM1-*) (*53*) fail to distinguish between pericytes and smooth muscle cells, which also have high *MCAM* and *PDGFRB*, and low *CD34*, *PTPRC*, and *PECAM1* expression (Table S5+S26). This suggests that a new, modified strategy may be needed to quantify pericytes histologically or to isolate them by FACS. Potentially useful markers to formulate a new strategy explicitly accounting for the overlap with smooth muscle cells may include *BCAM* (CD239), *CD248*, and *CD36* (Fig. 4B, Table S7).

The top five transcriptional markers of smooth muscle cells (0.22 ± 0.27% of ADSVF) were *MYH11*, *RERGL*, *ACTA2*, *TPM2*, and *TAGLN* (avg_logFC-sorted) and additionally *PLN*, *CNN1* and *ACTG2* (adj_p-sorted) (Fig. 4A, Table S26). DE surface marker genes included *MCAM* (CD146), *BCAM* (CD239), *ITGA3* (CD49c), *TNC* (CD175), and *JAG1* (CD339) (Fig. 4B, Table

S7). The unbiased list of smooth muscle cell DE genes compared with all ADSVF cells can be found in Table S26.

Seven transcriptionally distinct fibroblast subpopulations (P1-P7; 53.46 ± 12.74% of ADSVF) were identified (Table S3). DE genes of the fibroblast group compared with other ADSVF cell types included *DCN*, *CFD*, *APOD*, *CXCL14*, and *COL1A1* (Fig. 4A, Table S27) and DE surface marker genes included *CD248*, *PDGFRA* (CD140a), *SDC2* (CD362), *LRP1* (CD91), and *MRC2* (CD280) (Fig. 4B, Table S7). The largest subpopulation, P1, encompassed 24.30 ± 5.30% of ADSVF cells (Table S3) and the top DE genes compared with other fibroblast subpopulations included *CXCL14*, *APOE*, *APOD*, *CXCL12*, and *SLC5A3*, and the top DE surface markers were *VCAM1* (CD106) and *CD36* (Fig. S6A, Table S28). The second-largest subpopulation, P2, accounted for 10.56 ± 4.35% of ADSVF cells and the top DE genes compared with other fibroblast subpopulations were *CILP*, *ITGA11*, *ANGPTL2*, *PLA2G2A*, and *KLF6*. No DE surface marker genes were found to define this population (Fig. S6A, Table S28). A *PRG4*^{high}*C1QTNF3*+ population (P3) accounted for 10.37 ± 5.26% of ADSVF and top DE genes included *PRG4*, *FBN1*, *SFRP4*, *SEMA3C*, and *CD55*, and additional surface marker genes *CD248*, *PROCR* (CD201), *DPP4* (CD26), and *CD70*, compared with the other subpopulations (Fig. S6A, Table S28). P4 accounted for 3.71 ± 0.93% of ADSVF and DE genes included *TNC* (CD175), *POSTN*, *MT2A*, *MT1X*, *CCL2*, and additional surface marker genes *ICAM1* (CD54), *PDGFRB* (CD140b), and *CD36* (Fig. S6A, Table S28). Then, a subpopulation (P5) with high expression of the hedgehog genes *HHIP*, *TSPAN8*, *HHIP-AS1*, and *PTCH2* accounted for 2.27 ± 0.76% of ADSVF and DE genes included *IGFBP7*, *NRP1* (CD304), *BGN*, *SPARCL1*, and *NR2F2*, and additional DE surface marker genes *THBD* (CD141), *LIFR* (CD118), *F3* (CD142), and *CD302* (Fig. S6A, Table S28). The sixth, *COL8A1*+*COMP*+ population encompassed 2.00 ± 0.75% of ADSVF cells and top DE

genes included *COMP*, *PCSK5*, *BGN*, *MGP*, and *SFRP2*, and surface marker genes *TNC* (CD175), *F3* (CD142), and *LEPR* (CD295), compared with the other subpopulations (Fig. S6A, Table S28). Lastly, a small population of 0.26 ± 0.23% of ADSVF cells expressed genes typically associated with dendritic cells and the monocyte/macrophage lineage. Top DE genes included *C1QA*, *RNASE1*, *CD74*, *HLA-DRA*, and *CCL3*, and the top DE surface marker genes were *MRC1* (CD206), *CD74*, *CD163*, *CD36*, and *BST2* (CD317), compared with the other six subpopulations (Fig. S6A, Table S28). The unbiased lists of DE genes of these fibroblast subpopulations compared with all ADSVF populations can be found in Table S28.

The top expressed DE genes of myofibroblasts (0.41 ± 0.28% of ADSVF) included *TAGLN*, *ACTA2*, *TPM2*, *TPM1*, and *MYL9* (avg_logFC-sorted) and *AC027288.3*, *PRUNE2*, *ACTG2*, *CNN1*, and *ABCB5* (adj_p-sorted) (Fig. 4A, Table S29). DE surface marker genes included *CD9*, *SDC2* (CD362), *CD248*, *ITGAV* (CD51), and *FGFR1* (CD331) (Fig. 4B, Table S7+S29).

We identified two transcriptionally distinct pre fibrocyte subpopulations (0.16 ± 0.19% of ADSVF). The top DE genes of this group were *FBP1*, *SPP1*, *LIPA*, *FABP5*, and *CSTB* (Fig. 4A, Table S27), and DE surface marker genes included *ITGAX* (CD11c), *ALCAM* (CD166), *MSR1* (CD204), *CD52*, and *ADGRE2* (CD312) (Fig. 4B, Table S7). Early pre fibrocytes accounted for 0.07 ± 0.14% of ADSVF cells (Table S3) and DE genes included *RGS5*, *SPARCL1*, *APOLD1*, *GNG11*, and *RBP7*, and DE surface marker genes *CD52*, *MSR1* (CD204), *KDR* (CD309), *CDH5* (CD144), and *ITGA6* (CD49f), compared with late pre fibrocytes (Fig. S6B, Table S30). Late pre fibrocytes accounted for 0.09 ± 0.09% of ADSVF cells and positive DE genes included *TNXB*, *CFD*, *MGP*, *COL6A2*, and *DCN*, and surface marker genes *CD55*, *LRP1* (CD91), *CD44*, *CD74*, and *IL7R* (CD127) (Fig. S6B, Table S30). The unbiased list of these two subpopulations compared with all ADSVF cells can be found in Table S30.

Top DE genes of the fibrocyte population (1.56 ± 1.06% of ADSVF) included *LYVE1*, *C1QA*, *C1QB*, *CCL3*, and *LGMN* (avg_logFC-sorted), and additionally *MRC1* (CD206) and *C1QC* (adj_p-sorted) (Fig. 4A, Table S30). Additional DE surface marker genes included *LILRB5* (CD85c), *CD163*, *FCGR2B* (CD32b), and *CD14* (Fig. 4B, Table S7+30).

We found two monocyte populations (0.68 ± 1.02% of ADSVF). *CD14+VCAN+* monocytes accounted for 0.59 ± 1.00% of ADSVF cells (Table S3) and top five DE genes included *S100A9*, *LYZ*, *S100A8*, *FCN1*, and *CTSS* (avg_logFC-sorted) and additionally *EREG* (adj_p-sorted) (Fig. 4A, Table S31). DE surface marker genes included *C5AR1* (CD88), *ITGAX* (CD11c), *EVI2B* (CD361), *CSF3R* (CD114), and *CD300E* (Fig. 4B, Table S7+31). *FCGR3B+G0S2+* monocytes encompassed 0.09 ± 0.23% of ADSVF cells and top five DE genes were *S100A8*, *FCGR3B* (CD16b), *S100A9*, *MNDA*, and *G0S2* (avg_logFC-sorted), and additionally *CXCR2* (CD182), *CYP4F3*, and *AQP9* (adj_p-sorted) (Fig. 4A, Table S31). Additional DE surface marker genes included *TNFRSF10C* (CD263), *CXCR1* (CD181), and *SELL* (CD62l) (Fig. 4B, Table S7+31). These populations corresponded to mono1 and mono3 by Villani *et al.* (*79*).

We found four transcriptionally distinct macrophage populations (P1-P4; 10.81 ± 4.66% of ADSVF). The top five DE genes of the macrophage group were *CCL3*, *C1QA*, *RNASE1*, *CXCL8*, and *C1QB* (Fig. 4A, Table S27), and DE surface marker genes included *MRC1* (CD206), *CD14*, *CD83*, *CD163*, and *CD68* (Fig. 4B, Table S7). The largest population, P1, encompassed 8.23 ± 3.67% of ADSVF cells (Table S3) and DE genes compared with other macrophage subpopulations included *LYVE1*, *RNASE1*, *F13A1*, *PMP22*, and *JUN*, and DE surface marker genes *MRC1* (CD206), *LILRB5* (CD85c), *CD14*, *GYPC* (CD236), and *CD209* (Fig. S6C, Table S32). The second population accounted for 1.33 ± 0.81% of ADSVF cells and DE genes included *FABP5*, *APOC1*, *SPP1*, *APOE*, and *FABP4*, and surface marker genes *CD52*, *ITGAX* (CD11c), *ALCAM*

(CD166), *CD9*, and *SDC2* (CD362) (Fig. S6C, Table S32). The third subpopulation accounted for 1.16 ± 1.00% and DE genes included *FCN1*, *EREG*, *LYZ*, *AREG*, and *IL1B*, and surface marker genes *CD48*, *CLEC7A* (CD369), *ITGAX* (CD11c), *PLAUR* (CD87), and *CXCR4* (CD184), compared with the other macrophage subpopulations (Fig. S6C, Table S32). Lastly, the smallest population was sized 0.09 ± 0.13% of ADSVF cells (Table S3) and DE included *NKG7*, *CCL5*, *IL32*, *GNLY*, and *IL7R*, and DE surface marker genes *CD3D*, *KLRB1* (CD161), *CD69*, and *CXCR4* (CD184) (Fig. S6C, Table S32). The unbiased lists of DE genes of these four macrophage populations compared with all ADSVF cells can be found in Table S32.

We found six transcriptionally distinct dendritic cell subpopulations (3.43 ± 1.28% of ADSVF cells; Table S3). *CD1C+FCER1A+* dendritic cells accounted for 2.00 ± 1.13% of ADSVF cells (Table S3) and the top DE genes included *HLA-DRA*, *HLA-DPB1*, *HLA-DPA1*, *HLA-DQA1*, and *HLA-DQB1* (Fig. 4A, Table S33) and surface marker genes *CD74*, *CLEC10A* (CD301), *CD1C*, *IL1R2* (CD121b), and *PLAUR* (CD87) (Fig. 4B, Table S7+S33). This subpopulation corresponded to DC2 from (*79*). A second *CD1C+FCER1A+* subpopulation which additionally expressed *S100A9* was termed DC3 in (*79*) and accounted for only 0.08 ± 0.09% cells in ADSVF (Table S3). Top five DE genes included *FCER1A*, *LYZ*, *HLA-DPB1*, *HLA-DPA1*, and *HLA-DRA* (avg_logFC-sorted) and additionally *CD1C*, *PKIB*, *LGALS2*, and *JAML* (adj_p-sorted) (Fig. 4A, Table S33). Additional DE surface marker genes included *CLEC7A* (CD369), *ITGAX* (CD11c), *CD1D*, and *FLT3* (CD135) (Fig. 4B, Table S7+S33). Then, *CD1C-THBD-* dendritic cells, DC4 from (*79*), encompassed 0.77 ± 0.57% of ADSVF cells (Table S3). DE genes of this subpopulation included *LST1*, *FCGR3A* (CD16a), *AIF1*, *CTSS*, and *COTL1* (Fig. 4A, Table S33), and additional surface marker genes *C5AR1* (CD88), *LILRB2* (CD85d), *CD52*, and *SPN* (CD43) (Fig. 4B, Table S7+S33). The fourth, *CLEC9A+* subpopulation, DC1 in (*79*), accounted for 0.25 ± 0.14% of ADSVF cells

and DE genes included *CPVL*, *HLA-DPB1*, *HLA-DPA1*, *HLA-DRA*, and *CD74* (avg_logFC-sorted), and *LGALS2*, *WDFY4*, *S100B*, *CLEC9A* (CD370), and *TACSTD2* (adj_p-sorted) (Fig. 4A, Table S33). Additional DE surface marker genes included *SLAMF7* (CD319), *FLT3* (CD135), *BTLA* (CD272), and *SLAMF8* (CD353) (Fig. 4B, Table S7+S33). Then, a *CCL22+LAMP3+* subpopulation which was not defined by Villani and colleagues, accounted for 0.25 ± 0.15% of ADSVF cells. Top DE genes were *CCL22*, *CCR7* (CD179), *CCL17*, *BIRC3*, and *HLA-DPB1* (Fig. 4A, Table S33), and surface marker genes included *LAMP3* (CD208), *CD1E*, *CD1B*, and *FLT3* (CD135) (Fig. 4B, Table S7+S33). Lastly, the smallest subpopulation of 0.07 ± 0.06% were plasmacytoid dendritic cells, termed DC6 in (*79*). Their top DE genes included *JCHAIN*, *GZMB*, *IRF4*, *GPR183*, and *AREG* (avg_logFC-sorted) and *PLD4*, *BCL11A*, *LILRA4* (CD85g), and *MZB1* (adj_p-sorted) (Fig. 4A, Table S33). Additional DE surface marker genes included *CLEC4C* (CD303), *SEMA7A* (CD108), *CXCR3* (CD183), and *SELL* (CD62l) (Fig. 4B, Table S7+S33).

We detected a total of 15 transcriptionally distinguishable endothelial cell subpopulations or cell states, respectively (16.35 ± 8.91% of ADSVF; Table S3). DE genes of this group compared with other ADSVF cells included *FABP4*, *RBP7*, *CLDN5*, *SPARCL1*, and *VWF* (Fig. 4A, Table S27), and surface marker genes *CD36*, *CD300LG* (CD300g), *BST2* (CD317), *PECAM1* (CD31), and *CDH5* (CD144) (Fig. 4B, Table S7). The largest subpopulation, P1, accounted for 4.22 ± 2.69% of ADSVF cells (Table S3) and top DE genes compared with the other endothelial populations were *RGCC*, *IER2*, *DNAJB1*, *HES1*, and *HSPA1A*, and surface marker genes *CD300LG* (CD300g), *TNFRSF10D* (CD264), *ICAM1* (CD54), and *ICAM4* (CD173) (Fig. S6D, Table S34). An unbiased list of DE genes compared with all ADSVF populations can be found in Table S34. The second population (P2) encompassed 2.93 ± 1.97% of ADSVF cells and the positive DE genes compared with the other endothelial populations were *POSTN*, *TAGLN*, *NDUFA4L2*, *ACTA2*, and *IGFBP5*,

and DE surface marker genes included *PDGFRB* (CD140b), *CD248*, *CD44*, *DDR2* (CD167b), and *ITGB1* (CD29) (Fig. S6D, Table S34). A third population of *ACKR1+CLSTN2-ADRA2A-* endothelial cells accounted for 1.15 ± 1.15% of ADSVF cells and DE genes included *ACKR1* (CD234), *CLU*, *SELE* (CD62e), *AQP1*, and *PLVAP*, and additional surface marker genes were *CD74*, *LIFR* (CD118), and *IL1R1* (CD121a) (Fig. S6D, Table S34). A closely related, *ACKR1+CLSTN2+ADRA2A+* endothelial population (P4) accounted for 0.15 ± 0.13% of ADSVF cells (Fig. S6D, Table S34). DE genes were very similar, too: *SELE*, *ACKR1*, *SYNPO2*, *CLU*, and *TAGLN*, and additional surface marker genes were *SELP* (CD62p), *VCAM1* (CD106), and *CD44* (Fig. S6D, Table S34). The fifth, *ADRA2C+PDGFRB-* population (P5) accounted for 0.80 ± 0.50% and DE genes included *CXCL12*, *LTBP4*, *PODXL*, *FN1*, and *IGFBP3*, and surface marker genes *JAM2* (CD322), *ICAM2* (CD102), *ACE* (CD143), *JAG1* (CD339), and *CD9* (Fig. S6D, Table S34). An *ADRA2C+PDGFRB+* population (P6) accounted for 0.38 ± 0.28% of ADSVF cells and DE genes included *GJA4*, *NDUFA4L2*, *RGS5*, *CXCL12*, and *TAGLN*, and the surface marker genes *PDGFRB* (CD140b), *ENPEP* (CD249), *CD248*, *JAG1* (CD339), and *DDR2* (CD167b) (Fig. S6D, Table S34). *APLN+PGF+* endothelial cells (P7) encompassed 0.31 ± 0.20% of ADSVF cells and the top positive DE genes compared with the other endothelial populations included *LY6H*, *PGF*, *APLN*, *APOD*, and *COL4A1*, and the surface marker genes *CXCR4* (CD184), *TNFRSF4* (CD134), *CD9*, *MCAM* (CD146), and *CD93* (Fig. S6D, Table S34). Then, a *LYVE1+IL1B+* population (P8) accounted for 0.31 ± 0.35% of ADSVF cells and DE genes compared with other endothelial cells included *IL1B*, *LYZ*, *S100A9*, *CXCL8*, and *CCL3*, and surface marker genes *PTPRC* (CD45), *C5AR1* (CD88), *PLAUR* (CD87), *CXCR4* (CD184), and *CD44* (Fig. S6D, Table S34). A related, *LYVE1+LILRB5+* population (P9) accounted for 0.60 ± 0.53% of cells and DE genes included *C1QA*, *C1QB*, *LYVE1*, *CCL3*, and *CXCL8*, and surface marker genes *MRC1* (CD206), *LILRB5*

(CD85c), *CD163*, *CD14*, and *CD68* (Fig. S6D, Table S34). Then, we found two clusters of endothelial cells with lower gene expression compared with the other endothelial populations, which indicated that these may be low quality cells. However, they passed quality control and there was no objective argument to be made to exclude these two populations from the dataset. These low expression clusters 1 and 2 (P10+P11) accounted for 2.82 ± 1.87% and 1.21 ± 1.33% of ADSVF cells, respectively (Table S3, Fig. S6D). DE genes for P10 included *BST2* (CD317), *BTNL9*, *CD300LG* (CD300g), *CA4*, and *GIMAP7*, and additional surface marker genes were *CD36*, *CD59*, and *TNFSF10* (CD253) (Fig. S6D, Table S34). DE genes for P11 included *CFD*, *DCN*, *MGP*, *PRG4*, and *FBN1*, and surface marker genes *SELE* (CD62e), *CD44*, *CD163*, *MRC1* (CD206) (Fig. S6D, Table S34). We further found two subpopulations with a fibroblast-like gene signature: 0.34 ± 0.55% were *PDGFRA*^{low} fibroblast-like endothelial cells (P12) and 0.76 ± 0.44% of cells were classified as *PDGFRA*^{high} fibroblast-like endothelial cells (P13). Both populations stood out by expression of fibroblastic genes including *DCN*, *CFD*, and *CD248* (Fig. 4A+B). Then, 0.27 ± 0.24% of ADSVF endothelial cells had a NK/T cell gene signature (P14) including *CCL5*, *NKG7*, and *GNLY* (Fig. 4A). Lastly, a small hemoglobin gene-expressing population (P15) accounted for 0.09 ± 0.08% of ADSVF cells and top DE genes included *HBA2*, *HBA1*, *HBB*, *BTNL9*, and *EPAS1* (Fig. 4A, Table S34). Based on their concomitant lack of erythrocyte markers (*e.g. GYPA*) and expression of endothelial genes, we concluded that these cells were an endothelial subpopulation rather than erythroblasts (*141*). All lists with unbiased DE genes of these endothelial subpopulations compared with all ADSVF populations can be found in Table S34.

Early preadipocytes (0.21 ± 0.17% of ADSVF) were characterized by their distinct expression of adipogenesis-related genes including *PLIN1*, *ADIPOQ*, *THRSP*, and *PLIN4* (adj_p-sorted), and *RBP4*, *SAA1*, *G0S2*, *CRYAB* (avg_logFC-sorted) (Fig. 4A, Table S35). Interestingly, there was

transcriptional overlap with endothelial cells and pericytes, including the DE surface marker genes *ITGA1* (CD49a), *KDR* (CD309), *CDH5* (CD144), *MCAM* (CD146), and *CD300LG* (CD300g) (Fig. 4B, Table S7). Therefore, this population could as well be another endothelial subpopulation, and more investigations are needed to prove their identity.

We detected three *CD4*+ T cell subpopulations (4.73 ± 3.67% of ADSVF) and their top group DE genes were *IL7R* (CD127), *LTB*, *CXCR4* (CD184), *CD52*, and *IL32* (Fig. 4A, Table S27), and other surface marker genes were *CD69* and *CD2* (Fig. 4B, Table S7). The largest population (P1) accounted for 3.86 ± 2.74% of ADSVF cells (Table S3) and the top DE genes were *GZMA*, *CCL5*, *ANKRD28*, *KLRB1* (CD161), and *GZMK*, and additional DE surface marker genes included *CD52*, *CD99*, *ITGB1* (CD29), and *CD40LG* (CD154), compared with the other two subpopulations (Fig. S6E, Table S36). The second population accounted for 0.55 ± 1.23% of ADSVF cells and top DE genes compared with the other *CD4*+ T cells were *SELL* (CD62l), *CCR7* (CD197), *MT1X*, *MT2A*, *MT1G*, and additional DE surface marker genes included *IL6ST* (CD130), *CD55*, and *CD7* (Fig. S6E, Table S36). Lastly, *CD4*+ Tregs accounted for 0.31 ± 0.40% of ADSVF cells and top DE genes were *FOXP3*, *IKZF2*, *TIGIT*, *CTLA4* (CD152), and *PMAIP1*, and additional DE surface marker genes *TNFRSF9* (CD137), *TNFRSF18* (CD357), *CD27*, and *TNFRSF4* (CD134) (Fig. S6E, Table S36). The unbiased lists of DE genes compared with all ADSVF populations can be found in Table S36.

Three subpopulations of *CD8*+ T cells (2.80 ± 2.74% of ADSVF) were detected and DE genes compared with all ADSVF cells were *CCL5*, *NKG7*, *GNLY*, *GZMH*, and *CXCR4* (CD184) (Fig. 4A, Table S27), and additional DE surface marker genes were *CD3D*, *PTPRC* (CD45), *CD3G*, *CD52*, and *CD8A* (Fig. 4B, Table S7). The largest population encompassed 1.41 ± 1.43% of ADSVF cells (Table S3) and top DE genes compared with the other two *CD8*+ T subpopulations

included *GZMK*, *COTL1*, *DUSP4*, *RGS1*, and *XIST*, and the DE surface marker genes *CD44*, *CD27*, *CD74*, *CD2*, and *CRTAM* (CD355) (Fig. S6F, Table S37). The second subpopulation accounted for 1.07 ± 1.53% and according to (*77*), these are activated *CD8*+ T cells. Top DE genes included *GNLY*, *FGFBP2*, *GZMB*, *KLRD1* (CD94), and *FCGR3A* (CD16a), and additional surface marker genes were *SPN* (CD43), *ITGAL* (CD11a), and *CD247* (Fig. S6F, Table S37). The third and smallest subpopulation accounted for 0.31 ± 0.39% of ADSVF and were termed terminally differentiated effector cells (TEMRA) in accordance with the detailed T cell analysis by (*77*). Top DE genes compared with the other subpopulations included *KLRB1* (CD161), *CEBPD*, *LTB*, *IL7R*, and *FAM43A*, and additional surface marker genes included *CCR6* (CD196), *IL7R* (CD127), *NCR3* (CD337), and *DPP4* (CD26) (Fig. S6F, Table S37). The unbiased lists of DE genes comparing these *CD8*+ populations with all ADSVF cells can be found in Table S37.

Two transcriptionally distinct NK cell populations were detected (2.59 ± 1.66% of ADSVF). DE genes of these group compared with ADSVF included *GNLY*, *NKG7*, *GZMB*, *PRF1*, and *GZMA* (Fig. 4A, Table S27), and the top surface marker genes were *KLRD1* (CD94), *CD7*, *KLRB1* (CD161), *CD247*, and *FCGR3A* (CD16a) (Fig. 4B, Table S7). *FCGR3A*+ NK cells accounted for 2.09 ± 1.52% of ADSVF cells and DE genes compared with the other NK population included *FGFBP2*, *SPON2*, *PRF1*, *FCGR3A* (CD16a), and *ADGRG1*, and additional DE surface marker genes were *CD247*, *ITGB2* (CD18), *LAIR2* (CD306), and *HAVCR2* (CD366) (Fig. S6G, Table S38). *XCL1*+ NK cells accounted for 0.50 ± 0.32% of ADSVF and positive DE genes compared with *FCGR3A*+ NK cells included *LMNA*, *GZMK*, *XCL1*, *IL7R* (CD127), and *VIM*, and more surface marker genes were *CD44*, *TNFRSF18* (CD357), *CD83*, and *CRTAM* (CD355) (Fig. S6G, Table S38). The unbiased lists of DE genes comparing these NK cells with all ADSVF populations can be found in Table S38.

We further detected three small populations which clustered with T and NK cells (0.57 ± 0.53% of ADSVF), but also expressed gene signatures of endothelial cells (P1+P2) and fibroblasts (P3). As doublets were filtered prior to analysis using standardized methods, there was no objective reason to exclude these cells from the dataset. However, their identity will remain to be investigated. 0.15 ± 0.20% of ADSVF cells were T/NK cells with endothelial signature (P1), 0.05 ± 0.05% of ADSVF cells were a second T/NK population with endothelial signature (P2), and 0.37 ± 0.31% were T/NK cells with a fibroblast signature (Table S3, Fig. 4A+B). Their unbiased lists of DE genes compared with all ADSVF populations can be found in Table S38.

The top DE genes of B cells (0.16 ± 0.22% of ADSVF) included *CD79A*, *MS4A1* (CD20), *IGHM*, *IGKC*, and *CD37* (avg_logFC-sorted) and additionally *BANK1* and *TNFRSF13C* (CD268) (adj_p-sorted) (Fig. 4A, Table S39). Additional top DE surface marker genes included *CD22* and *FCRL2* (CD307b) (Fig. 4B, Table S7).

The top DE genes of plasma cells (0.13 ± 0.14% of ADSVF) included *IGLC2*, *JCHAIN*, *IGLC3*, *IGHM*, and *IGKC* (avg_logFC-sorted) and *IGHG1/2/3* (adj_p-sorted) (Fig. 4B, Table S39). Top DE surface marker genes included *CD79A*, *CD27*, *TNFRSF17* (CD269), *FCRL5* (CD307e), and *SDC1* (CD138) (Fig. 4B, Table S7).

The unique signature of mast cells (0.10 ± 0.12% of ADSVF) included the positive DE genes *TPSAB1*, *TPSB2*, *CTSG*, *CPA3*, and *HPGD* (Fig. 4A, Table S39) and the top DE surface marker genes included *SIGLEC8* (CD329), *KIT* (CD117), *IL5RA* (CD125), *SIGLEC6* (CD327), and *L1CAM* (CD171) (Fig. 4B, Table S7).

Lastly, two cycling/proliferating clusters were detected, accounting for 0.41 ± 0.34% and 0.12 ± 0.10% of ADSVF cells, respectively. Among others, the respective marker genes *TOP2A*, *MKI67*,

CENPF, and *STMN1* strongly drove these clusters. Thus, they were not further analyzed. However, their unbiased lists of DE genes can be found in Table S40.

Supplementary Tables

Data File S1: Supplementary Tables S1-11.

Table S1. BMAC+ADSVF sequencing summary

Table S2. BMAC populations

Table S3. ADSVF populations

Table S4. All genes detected in % BMAC cells

Table S5. All genes detected in % ADSVF cells

Table S6. Differentially expressed genes in BMAC-MSCs

Table S7. Surface marker genes

Table S8. Comparison of leptin receptor+ BMAC cells vs. unsorted cells

Table S9. BMAC proteins

Table S10. Adipose proteins

Table S11. Percentage of cells expressing transcripts for potentially therapeutic proteins which were detected by mass spectrometry

Data File S2: Supplementary Tables S12-S25. Marker genes of BMAC cell populations

Table S12. Top 5 differentially expressed genes across BMAC cell populations.

Table S13. *CD4*+ T cells

Table S14. *CD8*+ T cells

Table S15. Natural killer cells

Table S16. Monocytes

Table S17. *C1QB*+*APOE*+ cells

Table S18. Dendritic cells

Table S19. Granulocyte progenitors, low-density granulocytes, neutrophils, and eosinophils

Table S20. Basophils/mast cells

Table S21. Hematopoietic stem cells

Table S22. Erythroblast progenitors

Table S23. Megakaryocyte progenitors and thrombocytes

Table S24. B cells and plasma cells

Table S25. Cycling cell cluster

Data File S3: Supplementary Tables S26-S40.

Table S26. Pericytes and smooth muscle cells

Table S27. Top 5 differentially expressed genes per group.

Table S28. Fibroblasts

Table S29. Myofibroblasts

Table S30. Fibrocytes

Table S31. Monocytes

Table S32. Macrophages

Table S33. Dendritic cells

Table S34. Endothelial cells

Table S35. Early pre-adipocytes

Table S36. *CD4*+ T cells

Table S37. *CD8*+ T cells

Table S38. Natural killer cells

Table S39. B cells, plasma cells, and mast cells

Table S40. Cycling populations

REFERENCES AND NOTES

1. I. Berger, A. Ahmad, A. Bansal, T. Kapoor, D. Sipp, J. E. J. Rasko, Global distribution of businesses marketing stem cell-based interventions. *Cell Stem Cell* **19**, 158–162 (2016).

2. L. Turner, P. Knoepfler, Selling stem cells in the USA: Assessing the direct-to-consumer industry. *Cell Stem Cell* **19**, 154–157 (2016).

3. H. Taylor-Weiner, J. Graff Zivin, Medicine's wild west—Unlicensed stem-cell clinics in the United States. *N. Engl. J. Med.* **373**, 985–987 (2015).

4. D. Sipp, P. G. Robey, L. Turner, Clear up this stem-cell mess. *Nature* **561**, 455–457 (2018).

5. A. I. Caplan, Mesenchymal stem cells. *J. Orthop. Res.* **9**, 641–650 (1991).

6. A. I. Caplan, Adult mesenchymal stem cells: When, where, and how. *Stem Cells Int.* **2015**, 628767 (2015).

7. P. A. Zuk, M. Zhu, H. Mizuno, J. Huang, J. W. Futrell, A. J. Katz, P. Benhaim, H. P. Lorenz, M. H. Hedrick, Multilineage cells from human adipose tissue: Implications for cell-based therapies. *Tissue Eng.* **7**, 211–228 (2001).

8. A. I. Caplan, Mesenchymal stem cells: Time to change the name! *Stem Cells Transl. Med.* **6**, 1445–1451 (2017).

9. I. R. Murray, J. Chahla, S. J. Wordie, S. A. Shapiro, N. S. Piuzzi, R. M. Frank, J. Halbrecht, K. Okada, N. Nakamura, B. Mandelbaum, J. L. Dragoo, A. Biologics, J. Borg-Stein, A. Anz, A. Gobbi, A. H. Gomoll, B. J. Cole, C. Lattermann, C. Chu, D. A. Grande, D. B. F. Saris, D. Flanigan, E. Kon, G. F. Muschler, G. A. Malanga, G. Dummer, J. Farr, J. M. Tokish, K. P. Spindler, K. Horsch, K. Zaslav, L. F. McIntyre, N. A. Sgaglione, S. L. Sherman, S. Rodeo, T. M. Awan, C. T. Vangsness, Regulatory and ethical aspects of orthobiologic therapies. *Orthop. J. Sports Med.* **10**, 23259671221101626 (2022).

10. I. Grand View Research, Stem Cells Market Size, Share and Trends Analysis Report By Product (Adult Stem Cells, Human Embryonic Stem Cells), By Application, By Technology, By

Therapy, By End Use, By Region, And Segment Forecasts, 2022–2030, (978–1–68038-130-6, 2022); www.grandviewresearch.com/industry-analysis/stem-cells-market).

11. S. A. Shapiro, J. R. Arthurs, M. G. Heckman, J. M. Bestic, S. E. Kazmerchak, N. N. Diehl, A. C. Zubair, M. I. O'Connor, Quantitative T2 MRI mapping and 12-month follow-up in a randomized, blinded, placebo controlled trial of bone marrow aspiration and concentration for osteoarthritis of the knees. *Cartilage* **10**, 432–443 (2019).

12. S. A. Shapiro, S. E. Kazmerchak, M. G. Heckman, A. C. Zubair, M. I. O'Connor, A Prospective, A prospective, single-blind, placebo-controlled trial of bone marrow aspirate concentrate for knee osteoarthritis. *Am. J. Sports Med.* **45**, 82–90 (2017).

13. C. Centeno, J. Pitts, H. Al-Sayegh, M. Freeman, Efficacy of autologous bone marrow concentrate for knee osteoarthritis with and without adipose graft. *Biomed. Res. Int.* **2014**, 370621 (2014).

14. C. Centeno, Z. Fausel, I. Stemper, U. Azuike, E. Dodson, A randomized controlled trial of the treatment of rotator cuff tears with bone marrow concentrate and platelet products compared to exercise therapy: A midterm analysis. *Stem Cells Int.* **2020**, 59623 (2020).

15. K. Mautner, R. Bowers, K. Easley, Z. Fausel, R. Robinson, Functional outcomes following microfragmented adipose tissue versus bone marrow aspirate concentrate injections for symptomatic knee osteoarthritis. *Stem Cells Transl. Med.* **8**, 1149–1156 (2019).

16. D. Lu, B. Chen, Z. Liang, W. Deng, Y. Jiang, S. Li, J. Xu, Q. Wu, Z. Zhang, B. Xie, S. Chen, Comparison of bone marrow mesenchymal stem cells with bone marrow-derived mononuclear cells for treatment of diabetic critical limb ischemia and foot ulcer: A double-blind, randomized, controlled trial. *Diabetes Res. Clin. Pract.* **92**, 26–36 (2011).

17. J. W. Belk, J. J. Lim, C. Keeter, P. C. McCulloch, D. A. Houck, E. C. McCarty, R. M. Frank, M. J. Kraeutler, Patients with knee osteoarthritis who receive platelet-rich plasma or bone marrow aspirate concentrate injections have better outcomes than patients who receive hyaluronic acid: Systematic review and meta-analysis. *Art Ther.* **39**, 1714–1734 (2023).

18. A. Gobbi, G. P. Whyte, One-stage cartilage repair using a hyaluronic acid-based scaffold with activated bone marrow-derived mesenchymal stem cells compared with microfracture: Five-year follow-up. *Am. J. Sports Med.* **44**, 2846–2854 (2016).

19. A. W. Anz, R. Hubbard, N. K. Rendos, P. A. Everts, J. R. Andrews, J. G. Hackel, Bone marrow aspirate concentrate is equivalent to platelet-rich plasma for the treatment of knee osteoarthritis at 1 year: A prospective, randomized trial. *Orthop. J. Sports Med.* **8**, 2325967119900958 (2020).

20. A. Vega, M. A. Martin-Ferrero, F. Del Canto, M. Alberca, V. Garcia, A. Munar, L. Orozco, R. Soler, J. J. Fuertes, M. Huguet, A. Sanchez, J. Garcia-Sancho, Treatment of knee osteoarthritis with allogeneic bone marrow mesenchymal stem cells. *Transplantation* **99**, 1681–1690 (2015).

21. S. Janssens, C. Dubois, J. Bogaert, K. Theunissen, C. Deroose, W. Desmet, M. Kalantzi, L. Herbots, P. Sinnaeve, J. Dens, J. Maertens, F. Rademakers, S. Dymarkowski, O. Gheysens, J. Van Cleemput, G. Bormans, J. Nuyts, A. Belmans, L. Mortelmans, M. Boogaerts, F. Van de Werf, Autologous bone marrow-derived stem-cell transfer in patients with ST-segment elevation myocardial infarction: Double-blind, randomised controlled trial. *Lancet* **367**, 113–121 (2006).

22. L. Lu, C. Dai, Z. Zhang, H. Du, S. Li, P. Ye, Q. Fu, L. Zhang, X. Wu, Y. Dong, Y. Song, D. Zhao, Y. Pang, C. Bao, Treatment of knee osteoarthritis with intra-articular injection of autologous adipose-derived mesenchymal progenitor cells: A prospective, randomized, double-blind, active-controlled, phase IIb clinical trial. *Stem Cell. Res. Ther.* **10**, 143 (2019).

23. J. R. Garza, R. E. Campbell, F. P. Tjoumakaris, K. B. Freedman, L. S. Miller, D. Santa Maria, B. S. Tucker, Clinical efficacy of intra-articular mesenchymal stromal cells for the treatment of knee osteoarthritis: A double-blinded prospective randomized controlled clinical trial. *Am. J. Sports Med.* **48**, 588–598 (2020).

24. A. Mattei, J. Magalon, B. Bertrand, F. Grimaud, J. Revis, M. Velier, J. Veran, P. Dessi, F. Sabatier, A. Giovanni, Autologous adipose-derived stromal vascular fraction and scarred vocal folds: First clinical case report. *Stem Cell. Res. Ther.* **9**, 202 (2018).

25. P. Guillaume-Jugnot, A. Daumas, J. Magalon, E. Jouve, P. S. Nguyen, R. Truillet, S. Mallet, D. Casanova, L. Giraudo, J. Veran, F. Dignat-George, F. Sabatier, G. Magalon, B. Granel, Autologous adipose-derived stromal vascular fraction in patients with systemic sclerosis: 12-Month follow-up. *Rheumatology (Oxford)* **55**, 301–306 (2016).

26. P. Guillaume-Jugnot, A. Daumas, J. Magalon, N. Sautereau, J. Veran, G. Magalon, F. Sabatier, B. Granel, State of the art. Autologous fat graft and adipose tissue-derived stromal vascular fraction injection for hand therapy in systemic sclerosis patients. *Curr. Res. Transl. Med.* **64**, 35–42 (2016).

27. C. H. Jo, Y. G. Lee, W. H. Shin, H. Kim, J. W. Chai, E. C. Jeong, J. E. Kim, H. Shim, J. S. Shin, I. S. Shin, J. C. Ra, S. Oh, K. S. Yoon, Intra-articular injection of mesenchymal stem cells for the treatment of osteoarthritis of the knee: A proof-of-concept clinical trial. *Stem Cells* **32**, 1254–1266 (2014).

28. A. Dige, H. T. Hougaard, J. Agnholt, B. G. Pedersen, M. Tencerova, M. Kassem, K. Krogh, L. Lundby, Efficacy of injection of freshly collected autologous adipose tissue into perianal fistulas in patients with Crohn’s disease. *Gastroenterology* **156**, 2208–2216.e1 (2019).

29. E. Buscail, G. Le Cosquer, F. Gross, M. Lebrin, L. Bugarel, C. Deraison, N. Vergnolle, B. Bournet, C. Gilletta, L. Buscail, Adipose-derived stem cells in the treatment of perianal fistulas in Crohn’s disease: Rationale, clinical results and perspectives. *Int. J. Mol. Sci.* **22**, 9967 (2021).

30. Y. Yang, Z. Lan, J. Yan, Z. Tang, L. Zhou, D. Jin, Q. Jin, Effect of intra-knee injection of autologous adipose stem cells or mesenchymal vascular components on short-term outcomes in patients with knee osteoarthritis: An updated meta–analysis of randomized controlled trials. *Arthritis Res. Ther.* **25**, 147 (2023).

31. C. R. Chu, S. Rodeo, N. Bhutani, L. R. Goodrich, J. Huard, J. Irrgang, R. F. LaPrade, C. Lattermann, Y. Lu, B. Mandelbaum, J. Mao, L. McIntyre, A. Mishra, G. F. Muschler, N. S. Piuzzi, H. Potter, K. Spindler, J. M. Tokish, R. Tuan, K. Zaslav, W. Maloney, Optimizing clinical use of biologics in orthopaedic surgery: Consensus recommendations from the 2018 AAOS/NIH U-13 conference. *J. Am. Acad. Orthop. Surg.* **27**, e63–e64 (2019).

32. S. A. Rodeo, A. Bedi, 2019-2020 NFL and NFL physician society orthobiologics consensus statement. *Sports Health* **12**, 58–60 (2020).

33. K. P. Robb, J. Galipeau, Y. Shi, M. Schuster, I. Martin, S. Viswanathan, Failure to launch commercially-approved mesenchymal stromal cell therapies: What's the path forward? Proceedings of the International Society for Cell & Gene Therapy (ISCT) Annual Meeting Roundtable held in May 2023, Palais des Congres de Paris, Organized by the ISCT MSC Scientific Committee. *Cytotherapy* **26**, 413–417 (2023).

34. S. Ruoss, J. T. Walker, C. A. Nasamran, K. M. Fisch, C. J. Paez, J. N. Parekh, S. T. Ball, J. L. Chen, S. S. Ahmed, S. R. Ward, Strategies to identify mesenchymal stromal cells in minimally manipulated human bone marrow aspirate concentrate lack consensus. *Am. J. Sports Med.* **49**, 1313–1322 (2021).

35. A. A. o. O. Surgeons. (American Academy of Orthopaedic Surgeons, (2023), vol. 2023; doi.1001291092102.

36. I. R. Murray, T. R. McAdams, K. E. Hammond, F. S. Haddad, S. A. Rodeo, G. D. Abrams; P. a Group of American Professional Football, L. Bankston, A. Bedi, M. Boublik, M. Bowen, J. P. Bradley, D. E. Cooper, C. Craythorne, L. A. Curl, N. ElAttrache, D. S. Gazzaniga, K. Kaplan, E. E. Khalfayan, C. Larson, M. Pepe, M. D. Price, J. P. Schroeppel, J. Voos, G. Waslewski, R. West, The use of biologics in NFL athletes: An expert consensus of NFL team physicians. *Orthop. J. Sports Med.* **11**, 23259671221143778 (2023).

37. I. R. Murray, A. G. Geeslin, E. B. Goudie, F. A. Petrigliano, R. F. LaPrade, Minimum information for studies evaluating biologics in orthopaedics (MIBO): Platelet-rich plasma and mesenchymal stem cells. *J. Bone Joint Surg. Am.* **99**, 809–819 (2017).

38. R. M. Frank, S. L. Sherman, J. Chahla, J. L. Dragoo, B. Mandelbaum, A. Members of the Biologic, A. W. Anz, J. P. Bradley, C. R. Chu, B. J. Cole, J. Farr, D. C. Flanigan, A. H. Gomoll, J. Halbrecht, K. Horsch, C. Lattermann, P. Leucht, W. J. Maloney, L. F. McIntyre, I. Murray, G. F. Muschler, N. Nakamura, N. S. Piuzzi, S. A. Rodeo, D. B. F. Saris, W. O. Shaffer, S. A. Shapiro, K. P. Spindler, M. Steinwachs, J. M. Tokish, C. T. Vangsness, J. T. Watson, A. B.

Yanke, K. R. Zaslav, Biologic association annual summit: 2020 Report. *Orthop. J. Sports Med.* **9**, 23259671211015667 (2021).

39. A. F. Mavrogenis, V. Karampikas, A. Zikopoulos, S. Sioutis, D. Mastrokalos, D. Koulalis, M. M. Scarlat, P. Hernigou, Orthobiologics: A review. *Int. Orthop.* **47**, 1645–1662 (2023).

40. J. D. Lamplot, S. A. Rodeo, R. H. Brophy, A practical guide for the current use of biologic therapies in sports medicine. *Am. J. Sports Med.* **48**, 488–503 (2020).

41. S. A. Shapiro, Z. Master, J. R. Arthurs, K. Mautner, Tiered approach to considering orthobiologics for patients with musculoskeletal conditions. *Br. J. Sports Med.* **57**, 179–180 (2023).

42. O. Levy, R. Kuai, E. M. J. Siren, D. Bhere, Y. Milton, N. Nissar, M. De Biasio, M. Heinelt, B. Reeve, R. Abdi, M. Alturki, M. Fallatah, A. Almalik, A. H. Alhasan, K. Shah, J. M. Karp, Shattering barriers toward clinically meaningful MSC therapies. *Sci. Adv.* **6**, eaba6884 (2020).

43. I. R. Murray, J. Chahla, M. R. Safran, A. J. Krych, D. B. F. Saris, A. I. Caplan, R. F. LaPrade, G. International expert consensus on a cell therapy communication tool: DOSES. *J. Bone Joint Surg. Am.* **101**, 904–911 (2019).

44. M. Dominici, K. Le Blanc, I. Mueller, I. Slaper-Cortenbach, F. Marini, D. Krause, R. Deans, A. Keating, D. Prockop, E. Horwitz, Minimal criteria for defining multipotent mesenchymal stromal cells. The International Society for Cellular Therapy position statement. *Cytotherapy* **8**, 315–317 (2006).

45. N. S. Piuzzi, Z. B. Hussain, J. Chahla, M. E. Cinque, G. Moatshe, V. P. Mantripragada, G. F. Muschler, R. F. LaPrade, Variability in the preparation, reporting, and use of bone marrow aspirate concentrate in musculoskeletal disorders: A systematic review of the clinical orthopaedic literature. *J. Bone Joint Surg. Am.* **100**, 517–525 (2018).

46. E. Kwee, E. E. Herderick, T. Adams, J. Dunn, R. Germanowski, F. Krakosh, C. Boehm, J. Monnich, K. Powell, G. Muschler, Integrated colony imaging, analysis, and selection device for regenerative medicine. *SLAS Technol.* **22**, 217–223 (2017).

47. A. K. Majors, C. A. Boehm, H. Nitto, R. J. Midura, G. F. Muschler, Characterization of human bone marrow stromal cells with respect to osteoblastic differentiation. *J. Orthop. Res.* **15**, 546–557 (1997).

48. G. F. Muschler, C. Boehm, K. Easley, Aspiration to obtain osteoblast progenitor cells from human bone marrow: The influence of aspiration volume. *J. Bone Joint Surg. Am.* **79**, 1699–1709 (1997).

49. G. F. Muschler, H. Nitto, C. A. Boehm, K. A. Easley, Age- and gender-related changes in the cellularity of human bone marrow and the prevalence of osteoblastic progenitors. *J. Orthop. Res.* **19**, 117–125 (2001).

50. M. F. Pittenger, A. M. Mackay, S. C. Beck, R. K. Jaiswal, R. Douglas, J. D. Mosca, M. A. Moorman, D. W. Simonetti, S. Craig, D. R. Marshak, Multilineage potential of adult human mesenchymal stem cells. *Science* **284**, 143–147 (1999).

51. A. J. Engler, S. Sen, H. L. Sweeney, D. E. Discher, Matrix elasticity directs stem cell lineage specification. *Cell* **126**, 677–689 (2006).

52. B. Sacchetti, A. Funari, C. Remoli, G. Giannicola, G. Kogler, S. Liedtke, G. Cossu, M. Serafini, M. Sampaolesi, E. Tagliafico, E. Tenedini, I. Saggio, P. G. Robey, M. Riminucci, P. Bianco, No identical "Mesenchymal Stem Cells" at different times and sites: Human committed progenitors of distinct origin and differentiation potential are incorporated as adventitial cells in microvessels. *Stem Cell Rep.* **6**, 897–913 (2016).

53. M. Crisan, S. Yap, L. Casteilla, C. W. Chen, M. Corselli, T. S. Park, G. Andriolo, B. Sun, B. Zheng, L. Zhang, C. Norotte, P. N. Teng, J. Traas, R. Schugar, B. M. Deasy, S. Badylak, H. J. Buhring, J. P. Giacobino, L. Lazzari, J. Huard, B. Peault, A perivascular origin for mesenchymal stem cells in multiple human organs. *Cell Stem Cell* **3**, 301–313 (2008).

54. R. McBeath, D. M. Pirone, C. M. Nelson, K. Bhadriraju, C. S. Chen, Cell shape, cytoskeletal tension, and RhoA regulate stem cell lineage commitment. *Dev. Cell* **6**, 483–495 (2004).

55. L. Szabo (Washington Post, Washington Post, 2019), vol. 2022.

56. P. Jayaram, U. Ikpeama, J. B. Rothenberg, G. A. Malanga, Bone marrow-derived and adipose-derived mesenchymal stem cell therapy in primary knee osteoarthritis: A narrative review. *PM R* **11**, 177–191 (2019).

57. Y. B. Park, C. W. Ha, J. H. Rhim, H. J. Lee, Stem cell therapy for articular cartilage repair: Review of the entity of cell populations used and the result of the clinical application of each entity. *Am. J. Sports Med.* **46**, 2540–2552 (2018).

58. D. H. Lee, C. G. Kong, Y. W. Shin, S. Ahmed, A. A. Shetty, M. S. Moon, S. J. Kim, Which is better for articular cartilage regeneration, cultured stem cells or concentrated stromal cells? *Ann. Transl. Med.* **8**, 836 (2020).

59. P. Bourin, B. A. Bunnell, L. Casteilla, M. Dominici, A. J. Katz, K. L. March, H. Redl, J. P. Rubin, K. Yoshimura, J. M. Gimble, Stromal cells from the adipose tissue-derived stromal vascular fraction and culture expanded adipose tissue-derived stromal/stem cells: A joint statement of the International Federation for Adipose Therapeutics and Science (IFATS) and the International Society for Cellular Therapy (ISCT). *Cytotherapy* **15**, 641–648 (2013).

60. A. Cossarizza, H. D. Chang, A. Radbruch, A. Acs, D. Adam, S. Adam-Klages, W. W. Agace, N. Aghaeepour, M. Akdis, M. Allez, L. N. Almeida, G. Alvisi, G. Anderson, I. Andra, F. Annunziato, A. Anselmo, P. Bacher, C. T. Baldari, S. Bari, V. Barnaba, J. Barros-Martins, L. Battistini, W. Bauer, S. Baumgart, N. Baumgarth, D. Baumjohann, B. Baying, M. Bebawy, B. Becher, W. Beisker, V. Benes, R. Beyaert, A. Blanco, D. A. Boardman, C. Bogdan, J. G. Borger, G. Borsellino, P. E. Boulais, J. A. Bradford, D. Brenner, R. R. Brinkman, A. E. S. Brooks, D. H. Busch, M. Buscher, T. P. Bushnell, F. Calzetti, G. Cameron, I. Cammarata, X. Cao, S. L. Cardell, S. Casola, M. A. Cassatella, A. Cavani, A. Celada, L. Chatenoud, P. K. Chattopadhyay, S. Chow, E. Christakou, L. Cicin-Sain, M. Clerici, F. S. Colombo, L. Cook, A. Cooke, A. M. Cooper, A. J. Corbett, A. Cosma, L. Cosmi, P. G. Coulie, A. Cumano, L. Cvetkovic, V. D. Dang, C. Dang-Heine, M. S. Davey, D. Davies, S. De Biasi, G. Del Zotto, G. V. Dela Cruz, M. Delacher, S. Della Bella, P. Dellabona, G. Deniz, M. Dessing, J. P. Di Santo, A. Diefenbach, F. Dieli, A. Dolf, T. Dorner, R. J. Dress, D. Dudziak, M. Dustin, C. A. Dutertre, F. Ebner, S. B. G. Eckle, M. Edinger, P. Eede, G. R. A. Ehrhardt, M. Eich, P. Engel, B. Engelhardt, A. Erdei, C.

Esser, B. Everts, M. Evrard, C. S. Falk, T. A. Fehniger, M. Felipo-Benavent, H. Ferry, M. Feuerer, A. Filby, K. Filkor, S. Fillatreau, M. Follo, I. Forster, J. Foster, G. A. Foulds, B. Frehse, P. S. Frenette, S. Frischbutter, W. Fritzsche, D. W. Galbraith, A. Gangaev, N. Garbi, B. Gaudilliere, R. T. Gazzinelli, J. Geginat, W. Gerner, N. A. Gherardin, K. Ghoreschi, L. Gibellini, F. Ginhoux, K. Goda, D. I. Godfrey, C. Goettlinger, J. M. Gonzalez-Navajas, C. S. Goodyear, A. Gori, J. L. Grogan, D. Grummitt, A. Grutzkau, C. Haftmann, J. Hahn, H. Hammad, G. Hammerling, L. Hansmann, G. Hansson, C. M. Harpur, S. Hartmann, A. Hauser, A. E. Hauser, D. L. Haviland, D. Hedley, D. C. Hernandez, G. Herrera, M. Herrmann, C. Hess, T. Hofer, P. Hoffmann, K. Hogquist, T. Holland, T. Hollt, R. Holmdahl, P. Hombrink, J. P. Houston, B. F. Hoyer, B. Huang, F. P. Huang, J. E. Huber, J. Huehn, M. Hundemer, C. A. Hunter, W. Y. K. Hwang, A. Iannone, F. Ingelfinger, S. M. Ivison, H. M. Jack, P. K. Jani, B. Javega, S. Jonjic, T. Kaiser, T. Kalina, T. Kamradt, S. H. E. Kaufmann, B. Keller, S. L. C. Ketelaars, A. Khalilnezhad, S. Khan, J. Kisielow, P. Klenerman, J. Knopf, H. F. Koay, K. Kobow, J. K. Kolls, W. T. Kong, M. Kopf, T. Korn, K. Kriegsmann, H. Kristyanto, T. Kroneis, A. Krueger, J. Kuhne, C. Kukat, D. Kunkel, H. Kunze-Schumacher, T. Kurosaki, C. Kurts, P. Kvistborg, I. Kwok, J. Landry, O. Lantz, P. Lanuti, F. LaRosa, A. Lehuen, S. LeibundGut-Landmann, M. D. Leipold, L. Y. T. Leung, M. K. Levings, A. C. Lino, F. Liotta, V. Litwin, Y. Liu, H. G. Ljunggren, M. Lohoff, G. Lombardi, L. Lopez, M. Lopez-Botet, A. E. Lovett-Racke, E. Lubberts, H. Luche, B. Ludewig, E. Lugli, S. Lunemann, H. T. Maecker, L. Maggi, O. Maguire, F. Mair, K. H. Mair, A. Mantovani, R. A. Manz, A. J. Marshall, A. Martinez-Romero, G. Martrus, I. Marventano, W. Maslinski, G. Matarese, A. V. Mattioli, C. Maueroder, A. Mazzoni, J. McCluskey, M. McGrath, H. M. McGuire, I. B. McInnes, H. E. Mei, F. Melchers, S. Melzer, D. Mielenz, S. D. Miller, K. H. G. Mills, H. Minderman, J. Mjosberg, J. Moore, B. Moran, L. Moretta, T. R. Mosmann, S. Muller, G. Multhoff, L. E. Munoz, C. Munz, T. Nakayama, M. Nasi, K. Neumann, L. G. Ng, A. Niedobitek, S. Nourshargh, G. Nunez, J. E. O'Connor, A. Ochel, A. Oja, D. Ordonez, A. Orfao, E. Orlowski-Oliver, W. Ouyang, A. Oxenius, R. Palankar, I. Panse, K. Pattanapanyasat, M. Paulsen, D. Pavlinic, L. Penter, P. Peterson, C. Peth, J. Petriz, F. Piancone, W. F. Pickl, S. Piconese, M. Pinti, A. G. Pockley, M. J. Podolska, Z. Poon, K. Pracht, I. Prinz, C. E. M. Pucillo, S. A. Quataert, L. Quatrini, K. M. Quinn, H. Radbruch, T. Radstake, S. Rahmig, H. P. Rahn, B. Rajwa, G. Ravichandran, Y. Raz, J. A. Rebhahn, D. Recktenwald, D. Reimer, C. Reis e Sousa, E. B. M. Remmerswaal, L. Richter, L. G. Rico, A. Riddell, A. M.

Rieger, J. P. Robinson, C. Romagnani, A. Rubartelli, J. Ruland, A. Saalmuller, Y. Saeys, T. Saito, S. Sakaguchi, F. Sala-de-Oyanguren, Y. Samstag, S. Sanderson, I. Sandrock, A. Santoni, R. B. Sanz, M. Saresella, C. Sautes-Fridman, B. Sawitzki, L. Schadt, A. Scheffold, H. U. Scherer, M. Schiemann, F. A. Schildberg, E. Schimisky, A. Schlitzer, J. Schlosser, S. Schmid, S. Schmitt, K. Schober, D. Schraivogel, W. Schuh, T. Schuler, R. Schulte, A. R. Schulz, S. R. Schulz, C. Scotta, D. Scott-Algara, D. P. Sester, T. V. Shankey, B. Silva-Santos, A. K. Simon, K. M. Sitnik, S. Sozzani, D. E. Speiser, J. Spidlen, A. Stahlberg, A. M. Stall, N. Stanley, R. Stark, C. Stehle, T. Steinmetz, H. Stockinger, Y. Takahama, K. Takeda, L. Tan, A. Tarnok, G. Tiegs, G. Toldi, J. Tornack, E. Traggiai, M. Trebak, T. I. M. Tree, J. Trotter, J. Trowsdale, M. Tsoumakidou, H. Ulrich, S. Urbanczyk, W. van de Veen, M. van den Broek, E. van der Pol, S. Van Gassen, G. Van Isterdael, R. A. W. van Lier, M. Veldhoen, S. Vento-Asturias, P. Vieira, D. Voehringer, H. D. Volk, A. von Borstel, K. von Volkmann, A. Waisman, R. V. Walker, P. K. Wallace, S. A. Wang, X. M. Wang, M. D. Ward, K. A. Ward-Hartstonge, K. Warnatz, G. Warnes, S. Warth, C. Waskow, J. V. Watson, C. Watzl, L. Wegener, T. Weisenburger, A. Wiedemann, J. Wienands, A. Wilharm, R. J. Wilkinson, G. Willimsky, J. B. Wing, R. Winkelmann, T. H. Winkler, O. F. Wirz, A. Wong, P. Wurst, J. H. M. Yang, J. Yang, M. Yazdanbakhsh, L. Yu, A. Yue, H. Zhang, Y. Zhao, S. M. Ziegler, C. Zielinski, J. Zimmermann, A. Zychlinsky, Guidelines for the use of flow cytometry and cell sorting in immunological studies (second edition). *Eur. J. Immunol.* **49**, 1457–1973 (2019).

61. A. Butler, P. Hoffman, P. Smibert, E. Papalexi, R. Satija, Integrating single-cell transcriptomic data across different conditions, technologies, and species. *Nat. Biotechnol.* **36**, 411–420 (2018).

62. C. K. F. Chan, G. S. Gulati, R. Sinha, J. V. Tompkins, M. Lopez, A. C. Carter, R. C. Ransom, A. Reinisch, T. Wearda, M. Murphy, R. E. Brewer, L. S. Koepke, O. Marecic, A. Manjunath, E. Y. Seo, T. Leavitt, W. J. Lu, A. Nguyen, S. D. Conley, A. Salhotra, T. H. Ambrosi, M. R. Borrelli, T. Siebel, K. Chan, K. Schallmoser, J. Seita, D. Sahoo, H. Goodnough, J. Bishop, M. Gardner, R. Majeti, D. C. Wan, S. Goodman, I. L. Weissman, H. Y. Chang, M. T. Longaker, Identification of the human skeletal stem cell. *Cell* **175**, 43–56 e21 (2018).

63. A. I. Caplan, New MSC: MSCs as pericytes are Sentinels and gatekeepers. *J. Orthop. Res.* **35**, 1151–1159 (2017).

64. J. M. Gimble, A. J. Katz, B. A. Bunnell, Adipose-derived stem cells for regenerative medicine. *Circ. Res.* **100**, 1249–1260 (2007).

65. P. C. Baer, H. Geiger, Adipose-derived mesenchymal stromal/stem cells: Tissue localization, characterization, and heterogeneity. *Stem Cells Int.* **2012**, 812693 (2012).

66. C. Gui, J. Parson, G. A. Meyer, Harnessing adipose stem cell diversity in regenerative medicine. *APL Bioeng.* **5**, 021501 (2021).

67. K. Yoshimura, T. Shigeura, D. Matsumoto, T. Sato, Y. Takaki, E. Aiba-Kojima, K. Sato, K. Inoue, T. Nagase, I. Koshima, K. Gonda, Characterization of freshly isolated and cultured cells derived from the fatty and fluid portions of liposuction aspirates. *J. Cell. Physiol.* **208**, 64–76 (2006).

68. A. Miranville, C. Heeschen, C. Sengenes, C. A. Curat, R. Busse, A. Bouloumie, Improvement of postnatal neovascularization by human adipose tissue-derived stem cells. *Circulation* **110**, 349–355 (2004).

69. C. Sengenes, K. Lolmede, A. Zakaroff-Girard, R. Busse, A. Bouloumie, Preadipocytes in the human subcutaneous adipose tissue display distinct features from the adult mesenchymal and hematopoietic stem cells. *J. Cell. Physiol.* **205**, 114–122 (2005).

70. M. F. Pittenger, D. E. Discher, B. M. Peault, D. G. Phinney, J. M. Hare, A. I. Caplan, Mesenchymal stem cell perspective: Cell biology to clinical progress. *NPJ Regen. Med.* **4**, 22 (2019).

71. C. H. Jo, J. W. Chai, E. C. Jeong, S. Oh, J. S. Shin, H. Shim, K. S. Yoon, Intra-articular Injection of mesenchymal stem cells for the treatment of osteoarthritis of the knee: A 2-year follow-up study. *Am. J. Sports Med.* **45**, 2774–2783 (2017).

72. W. Zhou, J. Lin, K. Zhao, K. Jin, Q. He, Y. Hu, G. Feng, Y. Cai, C. Xia, H. Liu, W. Shen, X. Hu, H. Ouyang, Single-cell profiles and clinically useful properties of human mesenchymal stem cells of adipose and bone marrow origin. *Am. J. Sports Med.* **47**, 1722–1733 (2019).

73. R. Ghazanfari, D. Zacharaki, H. Li, H. Ching Lim, S. Soneji, S. Scheding, Human primary bone marrow mesenchymal stromal cells and their in vitro progenies display distinct transcriptional profile signatures. *Sci. Rep.* **7**, 10338 (2017).

74. S. Pinho, J. Lacombe, M. Hanoun, T. Mizoguchi, I. Bruns, Y. Kunisaki, P. S. Frenette, PDGFRα and CD51 mark human nestin+ sphere-forming mesenchymal stem cells capable of hematopoietic progenitor cell expansion. *J. Exp. Med.* **210**, 1351–1367 (2013).

75. P. van Galen, V. Hovestadt, M. H. Wadsworth Ii, T. K. Hughes, G. K. Griffin, S. Battaglia, J. A. Verga, J. Stephansky, T. J. Pastika, J. Lombardi Story, G. S. Pinkus, O. Pozdnyakova, I. Galinsky, R. M. Stone, T. A. Graubert, A. K. Shalek, J. C. Aster, A. A. Lane, B. E. Bernstein, Single-cell RNA-seq reveals AML hierarchies relevant to disease progression and immunity. *Cell* **176**, 1265–1281.e24 (2019).

76. S. B. Hay, K. Ferchen, K. Chetal, H. L. Grimes, N. Salomonis, The human cell atlas bone marrow single-cell interactive web portal. *Exp. Hematol.* **68**, 51–61 (2018).

77. P. A. Szabo, H. M. Levitin, M. Miron, M. E. Snyder, T. Senda, J. Yuan, Y. L. Cheng, E. C. Bush, P. Dogra, P. Thapa, D. L. Farber, P. A. Sims, Single-cell transcriptomics of human T cells reveals tissue and activation signatures in health and disease. *Nat. Commun.* **10**, 4706 (2019).

78. C. Yang, J. R. Siebert, R. Burns, Z. J. Gerbec, B. Bonacci, A. Rymaszewski, M. Rau, M. J. Riese, S. Rao, K. S. Carlson, J. M. Routes, J. W. Verbsky, M. S. Thakar, S. Malarkannan, Heterogeneity of human bone marrow and blood natural killer cells defined by single-cell transcriptome. *Nat. Commun.* **10**, 3931 (2019).

79. A. C. Villani, R. Satija, G. Reynolds, S. Sarkizova, K. Shekhar, J. Fletcher, M. Griesbeck, A. Butler, S. Zheng, S. Lazo, L. Jardine, D. Dixon, E. Stephenson, E. Nilsson, I. Grundberg, D. McDonald, A. Filby, W. Li, P. L. De Jager, O. Rozenblatt-Rosen, A. A. Lane, M. Haniffa, A. Regev, N. Hacohen, Single-cell RNA-seq reveals new types of human blood dendritic cells, monocytes, and progenitors. *Science* **356**, eaah4573 (2017).

80. C. G. Ziegler, R. Van Sloun, S. Gonzalez, K. E. Whitney, N. N. DePhillipo, M. I. Kennedy, G. J. Dornan, T. A. Evans, J. Huard, R. F. LaPrade, Characterization of growth factors, cytokines, and chemokines in bone marrow concentrate and platelet-rich plasma: A prospective analysis. *Am. J. Sports Med.* **47**, 2174–2187 (2019).

81. P. Kamat, F. S. Frueh, M. McLuckie, N. Sanchez-Macedo, P. Wolint, N. Lindenblatt, J. A. Plock, M. Calcagni, J. Buschmann, Adipose tissue and the vascularization of biomaterials: Stem cells, microvascular fragments and nanofat-a review. *Cytotherapy* **22**, 400–411 (2020).

82. A. Subramanian, P. Tamayo, V. K. Mootha, S. Mukherjee, B. L. Ebert, M. A. Gillette, A. Paulovich, S. L. Pomeroy, T. R. Golub, E. S. Lander, J. P. Mesirov, Gene set enrichment analysis: A knowledge-based approach for interpreting genome-wide expression profiles. *Proc. Natl. Acad. Sci. U.S.A.* **102**, 15545–15550 (2005).

83. A. I. Caplan, J. E. Dennis, Mesenchymal stem cells as trophic mediators. *J. Cell. Biochem.* **98**, 1076–1084 (2006).

84. S. Meirelles Lda, A. M. Fontes, D. T. Covas, A. I. Caplan, Mechanisms involved in the therapeutic properties of mesenchymal stem cells. *Cytokine Growth Factor Rev.* **20**, 419–427 (2009).

85. J. Chavez, N. A. Shah, S. Ruoss, R. E. Cuomo, S. R. Ward, T. K. Mackey, Online marketing practices of regenerative medicine clinics in US-Mexico border region: A web surveillance study. *Stem Cell. Res. Ther.* **12**, 189 (2021).

86. S. Rodeo, Stem Cells 101. *Am. J. Sports Med.* **49**, 1417–1420 (2021).

87. J. Vijay, M. F. Gauthier, R. L. Biswell, D. A. Louiselle, J. J. Johnston, W. A. Cheung, B. Belden, A. Pramatarova, L. Biertho, M. Gibson, M. M. Simon, H. Djambazian, A. Staffa, G. Bourque, A. Laitinen, J. Nystedt, M. C. Vohl, J. D. Fraser, T. Pastinen, A. Tchernof, E. Grundberg, Single-cell analysis of human adipose tissue identifies depot and disease specific cell types. *Nat. Metab.* **2**, 97–109 (2020).

88. A. C. Zannettino, S. Paton, A. Arthur, F. Khor, S. Itescu, J. M. Gimble, S. Gronthos, Multipotential human adipose-derived stromal stem cells exhibit a perivascular phenotype in vitro and in vivo. *J. Cell. Physiol.* **214**, 413–421 (2008).

89. J. Galipeau, L. Sensebe, Mesenchymal stromal cells: Clinical challenges and therapeutic opportunities. *Cell Stem Cell* **22**, 824–833 (2018).

90. S. Viswanathan, Y. Shi, J. Galipeau, M. Krampera, K. Leblanc, I. Martin, J. Nolta, D. G. Phinney, L. Sensebe, Mesenchymal stem versus stromal cells: International Society for Cell & Gene Therapy (ISCT®) Mesenchymal Stromal Cell committee position statement on nomenclature. *Cytotherapy* **21**, 1019–1024 (2019).

91. Food and Drug Administration (21 July 2020).

92. B. O. Zhou, R. Yue, M. M. Murphy, J. G. Peyer, S. J. Morrison, Leptin-receptor-expressing mesenchymal stromal cells represent the main source of bone formed by adult bone marrow. *Cell Stem Cell* **15**, 154–168 (2014).

93. X. Gao, M. M. Murphy, J. G. Peyer, Y. Ni, M. Yang, Y. Zhang, J. Guo, N. Kara, C. Embree, A. Tasdogan, J. M. Ubellacker, G. M. Crane, S. Fang, Z. Zhao, B. Shen, S. J. Morrison, Leptin receptor$^+$ cells promote bone marrow innervation and regeneration by synthesizing nerve growth factor. *Nat. Cell Biol.* **25**, 1746–1757 (2023).

94. N. Kara, Y. Xue, Z. Zhao, M. M. Murphy, S. Comazzetto, A. Lesser, L. Du, S. J. Morrison, Endothelial and Leptin Receptor$^+$ cells promote the maintenance of stem cells and hematopoiesis in early postnatal murine bone marrow. *Dev. Cell* **58**, 348–360.e6 (2023).

95. A. Paganelli, L. Benassi, E. Rossi, C. Magnoni, Extracellular matrix deposition by adipose-derived stem cells and fibroblasts: A comparative study. *Arch. Dermatol. Res.* **312**, 295–299 (2020).

96. R. A. Denu, S. Nemcek, D. D. Bloom, A. D. Goodrich, J. Kim, D. F. Mosher, P. Hematti, Fibroblasts and mesenchymal stromal/stem cells are phenotypically indistinguishable. *Acta Haematol.* **136**, 85–97 (2016).

97. M. Soundararajan, S. Kannan, Fibroblasts and mesenchymal stem cells: Two sides of the same coin? *J. Cell. Physiol.* **233**, 9099–9109 (2018).

98. S. Debnath, A. R. Yallowitz, J. McCormick, S. Lalani, T. Zhang, R. Xu, N. Li, Y. Liu, Y. S. Yang, M. Eiseman, J. H. Shim, M. Hameed, J. H. Healey, M. P. Bostrom, D. A. Landau, M. B. Greenblatt, Discovery of a periosteal stem cell mediating intramembranous bone formation. *Nature* **562**, 133–139 (2018).

99. M. B. Murphy, K. Moncivais, A. I. Caplan, Mesenchymal stem cells: Environmentally responsive therapeutics for regenerative medicine. *Exp. Mol. Med.* **45**, e54 (2013).

100. R. Jaeschke, J. Singer, G. H. Guyatt, Measurement of health status. *Control. Clin. Trials* **10**, 407–415 (1989).

101. S. Ruoss, S. T. Ball, S. N. Dorn, J. N. Parekh, A. J. Whitehead, A. J. Engler, S. R. Ward, Acetabular bone marrow aspiration during total hip arthroplasty. *J. Am. Acad. Orthop. Surg.* **29**, e815–e819 (2021).

102. G. Cattoretti, R. Schiro, A. Orazi, D. Soligo, M. P. Colombo, Bone marrow stroma in humans: Anti-nerve growth factor receptor antibodies selectively stain reticular cells in vivo and in vitro. *Blood* **81**, 1726–1738 (1993).

103. E. Jones, D. McGonagle, Human bone marrow mesenchymal stem cells in vivo. *Rheumatology (Oxford)* **47**, 126–131 (2008).

104. Y. Kfoury, D. T. Scadden, Mesenchymal cell contributions to the stem cell niche. *Cell Stem Cell* **16**, 239–253 (2015).

105. D. Zemmour, R. Zilionis, E. Kiner, A. M. Klein, D. Mathis, C. Benoist, Single-cell gene expression reveals a landscape of regulatory T cell phenotypes shaped by the TCR. *Nat. Immunol.* **19**, 291–301 (2018).

106. L. Arruvito, F. Payaslian, P. Baz, A. Podhorzer, A. Billordo, J. Pandolfi, G. Semeniuk, E. Arribalzaga, L. Fainboim, Identification and clinical relevance of naturally occurring human $CD8^{+}HLA^{-}DR^{+}$ regulatory T cells. *J. Immunol.* **193**, 4469–4476 (2014).

107. D. P. Saraiva, A. Jacinto, P. Borralho, S. Braga, M. G. Cabral, HLA-DR in cytotoxic T lymphocytes predicts breast cancer patients' response to neoadjuvant chemotherapy. *Front. Immunol.* **9**, 2605 (2018).

108. D. Pellin, M. Loperfido, C. Baricordi, S. L. Wolock, A. Montepeloso, O. K. Weinberg, A. Biffi, A. M. Klein, L. Biasco, A comprehensive single cell transcriptional landscape of human hematopoietic progenitors. *Nat. Commun.* **10**, 2395 (2019).

109. P. Davizon-Castillo, J. W. Rowley, M. T. Rondina, Megakaryocyte and platelet transcriptomics for discoveries in human health and disease. *Arterioscler. Thromb. Vasc. Biol.* **40**, 1432–1440 (2020).

110. J. E. Wither, S. D. Prokopec, B. Noamani, N. H. Chang, D. Bonilla, Z. Touma, C. Avila-Casado, H. N. Reich, J. Scholey, P. R. Fortin, P. C. Boutros, C. Landolt-Marticorena, Identification of a neutrophil-related gene expression signature that is enriched in adult systemic lupus erythematosus patients with active nephritis: Clinical/pathologic associations and etiologic mechanisms. *PLOS ONE* **13**, e0196117 (2018).

111. C. B. Read, J. L. Kuijper, S. A. Hjorth, M. D. Heipel, X. Tang, A. J. Fleetwood, J. L. Dantzler, S. N. Grell, J. Kastrup, C. Wang, C. S. Brandt, A. J. Hansen, N. R. Wagtmann, W. Xu, V. W. Stennicke, Cutting edge: Identification of neutrophil PGLYRP1 as a ligand for TREM-1. *J. Immunol.* **194**, 1417–1421 (2015).

112. M. Cundall, Y. Sun, C. Miranda, J. B. Trudeau, S. Barnes, S. E. Wenzel, Neutrophil-derived matrix metalloproteinase-9 is increased in severe asthma and poorly inhibited by glucocorticoids. *J. Allergy Clin. Immunol.* **112**, 1064–1071 (2003).

113. H. F. Rosenberg, Eosinophil-derived neurotoxin/RNase 2: Connecting the past, the present and the future. *Curr. Pharm. Biotechnol.* **9**, 135–140 (2008).

114. F. K. Hamey, W. W. Y. Lau, I. Kucinski, X. Wang, E. Diamanti, N. K. Wilson, B. Gottgens, J. S. Dahlin, Single-cell molecular profiling provides a high-resolution map of basophil and mast cell development. *Allergy* **76**, 1731–1742 (2021).

115. A. Ramesh, R. D. Schubert, A. L. Greenfield, R. Dandekar, R. Loudermilk, J. J. Sabatino, Jr., M. T. Koelzer, E. B. Tran, K. Koshal, K. Kim, A. K. Probstel, D. Banerji; University of California, San Francisco MS-EPIC Team, C. Y. Guo, A. J. Green, R. M. Bove, J. L. DeRisi, J. M. Gelfand, B. A. C. Cree, S. S. Zamvil, S. E. Baranzini, S. L. Hauser, M. R. Wilson, A pathogenic and clonally expanded B cell transcriptome in active multiple sclerosis. *Proc. Natl. Acad. Sci. U.S.A.* **117**, 22932–22943 (2020).

116. S. V. Kozyrev, A. K. Abelson, J. Wojcik, A. Zaghlool, M. V. Linga Reddy, E. Sanchez, I. Gunnarsson, E. Svenungsson, G. Sturfelt, A. Jonsen, L. Truedsson, B. A. Pons-Estel, T. Witte, S. D'Alfonso, N. Barizzone, M. G. Danieli, C. Gutierrez, A. Suarez, P. Junker, H. Laustrup, M. F. Gonzalez-Escribano, J. Martin, H. Abderrahim, M. E. Alarcon-Riquelme, Functional variants in the B-cell gene BANK1 are associated with systemic lupus erythematosus. *Nat. Genet.* **40**, 211–216 (2008).

117. C. N. Morrell, A. A. Aggrey, L. M. Chapman, K. L. Modjeski, Emerging roles for platelets as immune and inflammatory cells. *Blood* **123**, 2759–2767 (2014).

118. F. O. Martinez, S. Gordon, The M1 and M2 paradigm of macrophage activation: Time for reassessment. *F1000Prime Rep* **6**, 13 (2014).

119. H. Y. Lim, S. Y. Lim, C. K. Tan, C. H. Thiam, C. C. Goh, D. Carbajo, S. H. S. Chew, P. See, S. Chakarov, X. N. Wang, L. H. Lim, L. A. Johnson, J. Lum, C. Y. Fong, A. Bongso, A. Biswas, C. Goh, M. Evrard, K. P. Yeo, R. Basu, J. K. Wang, Y. Tan, R. Jain, S. Tikoo, C. Choong, W. Weninger, M. Poidinger, E. R. Stanley, M. Collin, N. S. Tan, L. G. Ng, D. G. Jackson, F. Ginhoux, V. Angeli, Hyaluronan receptor LYVE-1-expressing macrophages maintain arterial tone through hyaluronan-mediated regulation of smooth muscle cell collagen. *Immunity* **49**, 1191 (2018).

120. A. Woodfin, M. B. Voisin, S. Nourshargh, PECAM-1: A multi-functional molecule in inflammation and vascular biology. *Arterioscler. Thromb. Vasc. Biol.* **27**, 2514–2523 (2007).

121. T. Torisu, K. Torisu, I. H. Lee, J. Liu, D. Malide, C. A. Combs, X. S. Wu, Rovira, II, M. M. Fergusson, R. Weigert, P. S. Connelly, M. P. Daniels, M. Komatsu, L. Cao, T. Finkel, Autophagy regulates endothelial cell processing, maturation and secretion of von Willebrand factor. *Nat. Med.* **19**, 1281–1287 (2013).

122. W. Feng, L. Chen, P. K. Nguyen, S. M. Wu, G. Li, Single cell analysis of endothelial cells identified organ-specific molecular signatures and heart-specific cell populations and molecular features. *Front. Cardiovasc. Med.* **6**, 165 (2019).

123. L. Sauteur, A. Krudewig, L. Herwig, N. Ehrenfeuchter, A. Lenard, M. Affolter, H. G. Belting, Cdh5/VE-cadherin promotes endothelial cell interface elongation via cortical actin polymerization during angiogenic sprouting. *Cell Rep.* **9**, 504–513 (2014).

124. L. Muhl, G. Genove, S. Leptidis, J. Liu, L. He, G. Mocci, Y. Sun, S. Gustafsson, B. Buyandelger, I. V. Chivukula, A. Segerstolpe, E. Raschperger, E. M. Hansson, J. L. M. Bjorkegren, X. R. Peng, M. Vanlandewijck, U. Lendahl, C. Betsholtz, Single-cell analysis uncovers fibroblast heterogeneity and criteria for fibroblast and mural cell identification and discrimination. *Nat. Commun.* **11**, 3953 (2020).

125. A. N. Shami, X. Zheng, S. K. Munyoki, Q. Ma, G. L. Manske, C. D. Green, M. Sukhwani, K. E. Orwig, J. Z. Li, S. S. Hammoud, Single-cell RNA sequencing of human, macaque, and mouse testes uncovers conserved and divergent features of mammalian spermatogenesis. *Dev. Cell* **54**, 529–547.e12 (2020).

126. D. C. Guo, H. Pannu, V. Tran-Fadulu, C. L. Papke, R. K. Yu, N. Avidan, S. Bourgeois, A. L. Estrera, H. J. Safi, E. Sparks, D. Amor, L. Ades, V. McConnell, C. E. Willoughby, D. Abuelo, M. Willing, R. A. Lewis, D. H. Kim, S. Scherer, P. P. Tung, C. Ahn, L. M. Buja, C. S. Raman, S. S. Shete, D. M. Milewicz, Mutations in smooth muscle alpha-actin (ACTA2) lead to thoracic aortic aneurysms and dissections. *Nat. Genet.* **39**, 1488–1493 (2007).

127. Z. Wang, D. Z. Wang, G. C. Pipes, E. N. Olson, Myocardin is a master regulator of smooth muscle gene expression. *Proc. Natl. Acad. Sci. U.S.A.* **100**, 7129–7134 (2003).

128. R. Zhu, O. Gires, L. Zhu, J. Liu, J. Li, H. Yang, G. Ju, J. Huang, W. Ge, Y. Chen, Z. Lu, H. Wang, TSPAN8 promotes cancer cell stemness via activation of sonic Hedgehog signaling. *Nat. Commun.* **10**, 2863 (2019).

129. A. Horn, K. Palumbo, C. Cordazzo, C. Dees, A. Akhmetshina, M. Tomcik, P. Zerr, J. Avouac, J. Gusinde, J. Zwerina, H. Roudaut, E. Traiffort, M. Ruat, O. Distler, G. Schett, J. H. Distler, Hedgehog signaling controls fibroblast activation and tissue fibrosis in systemic sclerosis. *Arthritis Rheum.* **64**, 2724–2733 (2012).

130. G. Untergasser, R. Gander, C. Lilg, G. Lepperdinger, E. Plas, P. Berger, Profiling molecular targets of TGF-beta1 in prostate fibroblast-to-myofibroblast transdifferentiation. *Mech. Ageing Dev.* **126**, 59–69 (2005).

131. S. G. Chong, S. Sato, M. Kolb, J. Gauldie, Fibrocytes and fibroblasts-Where are we now. *Int. J. Biochem. Cell Biol.* **116**, 105595 (2019).

132. R. A. Reilkoff, R. Bucala, E. L. Herzog, Fibrocytes: Emerging effector cells in chronic inflammation. *Nat. Rev. Immunol.* **11**, 427–435 (2011).

133. M. Chandran, S. A. Phillips, T. Ciaraldi, R. R. Henry, Adiponectin: More than just another fat cell hormone? *Diabetes Care* **26**, 2442–2450 (2003).

134. J. M. Moreno-Navarrete, F. Ortega, M. Serrano, J. I. Rodriguez-Hermosa, W. Ricart, G. Mingrone, J. M. Fernandez-Real, CIDEC/FSP27 and PLIN1 gene expression run in parallel to mitochondrial genes in human adipose tissue, both increasing after weight loss. *Int. J. Obes. (Lond)* **38**, 865–872 (2014).

135. T. Stuart, A. Butler, P. Hoffman, C. Hafemeister, E. Papalexi, W. M. Mauck, 3rd, Y. Hao, M. Stoeckius, P. Smibert, R. Satija, Comprehensive integration of single-cell data. *Cell* **177**, 1888–1902.e21 (2019).

136. S. Tyanova, T. Temu, P. Sinitcyn, A. Carlson, M. Y. Hein, T. Geiger, M. Mann, J. Cox, The Perseus computational platform for comprehensive analysis of (prote)$_{omics}$ data. *Nat. Methods* **13**, 731–740 (2016).

137. Y. Benjamini, Y. Hochberg, Controlling the false discovery rate: A practical and powerful approach to multiple testing. *J. R. Stat. Soc. B. Methodol.* **57**, 289–300 (1995).

138. Y. Perez-Riverol, J. Bai, C. Bandla, D. Garcia-Seisdedos, S. Hewapathirana, S. Kamatchinathan, D. J. Kundu, A. Prakash, A. Frericks-Zipper, M. Eisenacher, M. Walzer, S. Wang, A. Brazma, J. A. Vizcaino, The PRIDE database resources in 2022: A hub for mass spectrometry-based proteomics evidences. *Nucleic Acids Res.* **50**, D543–D552 (2022).

139. C. Tripodo, R. Porcasi, C. Guarnotta, S. Ingrao, V. Campisi, A. M. Florena, V. Franco, C1q production by bone marrow stromal cells. *Scand. J. Immunol.* **65**, 308–309 (2007).

140. M. A. Cassatella, N. K. Ostberg, N. Tamassia, O. Soehnlein, Biological roles of neutrophil-derived granule proteins and cytokines. *Trends Immunol.* **40**, 648–664 (2019).

141. J. T. Butcher, T. Johnson, J. Beers, L. Columbus, B. E. Isakson, Hemoglobin α in the blood vessel wall. *Free Radic. Biol. Med.* **73**, 136–142 (2014).
